# Comparative efficacy and safety of warfarin care bundles and novel oral anticoagulants in patients with atrial fibrillation: a systematic review and network meta-analysis

**DOI:** 10.1038/s41598-019-57370-2

**Published:** 2020-01-20

**Authors:** Siok Shen Ng, Nai Ming Lai, Surakit Nathisuwan, Nowrozy Kamar Jahan, Piyameth Dilokthornsakul, Khachen Kongpakwattana, William Hollingworth, Nathorn Chaiyakunapruk

**Affiliations:** 1 School of Pharmacy, Monash University Malaysia, Bandar Sunway, Malaysia; 2 Jeffrey Cheah School of Medicine and Health Sciences, Monash University Malaysia, Bandar Sunway, Malaysia; 3 Department of Pharmacy, Hospital Melaka, Melaka, Malaysia; 4 School of Medicine, Taylor’s University, Selangor, Malaysia; 5 Clinical Pharmacy Division, Department of Pharmacy, Faculty of Pharmacy, Mahidol University, Bangkok, Thailand; 6 Center of Pharmaceutical Outcomes Research (CPOR), Department of Pharmacy Practice, Faculty of Pharmaceutical Sciences, Naresuan University, Phitsanulok, Thailand; 7 Department of Population Health Sciences, Bristol Medical School, University of Bristol, Bristol, BS8 1NU UK; 8 College of Pharmacy, University of Utah, Salt Lake City, Utah USA; 9 Asian Centre for Evidence Synthesis in Population, Implementation and Clinical Outcomes (PICO), Health and Well-being Cluster, Global Asia in the 21st Century (GA21) Platform, Monash University Malaysia, Bandar Sunway, Selangor Malaysia

**Keywords:** Cardiology, Atrial fibrillation

## Abstract

Warfarin care bundles (e.g. genotype-guided warfarin dosing, patient’s self-testing [PST] or patient’s self-management [PSM] and left atrial appendage closure) are based on the concept of combining several interventions to improve anticoagulation care. NOACs are also introduced for stroke prevention in atrial fibrillation (SPAF). However, these interventions have not been compared in head-to-head trials yet. We did a network meta-analysis based on a systematic review of randomized controlled trials comparing anticoagulant interventions for SPAF. Studies comparing these interventions in adults, whether administered alone or as care bundles were included in the analyses. The primary efficacy outcome was stroke and the primary safety outcome was major bleeding. Thirty-seven studies, involving 100,142 patients were assessed. Compared to usual care, PSM significantly reduced the risk of stroke (risk ratio [RR] 0.24, 95% CI 0.08–0.68). For major bleeding, edoxaban 60 mg (0.80, 0.71–0.90), edoxaban 30 mg (0.48, 0.42–0.56), and dabigatran 110 mg (0.81, 0.71–0.94) significantly reduced the risk of major bleeding compared with usual warfarin care. Cluster rank plot incorporating stroke and major bleeding outcomes indicates that some warfarin care bundles perform as well as NOACs. Both interventions are therefore viable options to be considered for SPAF. Additional studies including head-to-head trials and cost-effectiveness evaluation are still warranted.

## Introduction

For many years, warfarin has been the only effective oral anticoagulant for stroke prevention in patients with atrial fibrillation (SPAF)^1^. Due to its complex pharmacodynamic and pharmacokinetic profile, constant monitoring of anticoagulation effect through international normalized ratio (INR) is required to ensure optimal level of anticoagulation. The need for frequent monitoring may result in physical, psychological, social and financial consequences for the patient and the healthcare team^2^. Due to the perceived risks and inconvenience, warfarin remains underused^3^.

The concept of using more than one intervention together to improve patient care is called “a care bundle”^4^. This care bundle concept has been shown to be effective in improving patient outcomes in various disease models. Since several interventions have been shown to improve quality of anticoagulation control and outcomes, it is therefore logical to apply this care bundle concept to warfarin therapy. Examples of warfarin care bundles are the employment of genotype guidance into warfarin dosing, patient’s self-monitoring using point-of-care devices and left atrial appendage closure (LAAC). These pragmatic warfarin care bundles have the potential in improving patient’s convenience and the overall quality of warfarin care by resulting in fewer stroke and bleeding complications^2,5^.

Recently, novel oral anticoagulants (NOACs) have been introduced. These agents have fixed dosing and predictable pharmacokinetics, which eliminates the need for routine monitoring^6^. Although NOACs deliver on the promise of convenience, uncertainties remain surrounding their uses^7^. Without routine anticoagulation test, patient’s adherence could not be assessed. In some circumstances, the degree of anticoagulation may need to be assessed such as during severe haemorrhages or emergent surgery. Furthermore, safety data of NOACs in certain populations are still limited such as those with severe renal insufficiency. NOACs and their reversal agents have high acquisition costs, which can limit their accesses especially in low-income countries.

With the growing anticoagulant armamentarium, it is becoming more challenging to compare the efficacy and safety of these interventions for SPAF. Unfortunately, there are no head-to-head trials yet, comparing NOACs with each other or with warfarin care bundles. The degree of benefit of NOACs compared with warfarin depends on the patient-time in therapeutic range (TTR)^8^. To date, there is limited evidence to support the superiority of NOACs in preventing stroke over well-controlled warfarin therapy (TTR ≥ 65%) except for dabigatran 150 mg and apixaban^9–12^. Therefore, for anticoagulation centres with TTR < 65%, the possible choices are either switching to NOACs or investing additional resources to adopt warfarin care bundles. Each intervention has different clinical implications for patients, clinicians and decision-makers to consider. Hence, this study aimed to compare anticoagulant interventions including warfarin care bundles and NOACs for SPAF, by performing a comprehensive systematic review and network meta-analysis.

## Methods

This study was registered with PROSPERO (CRD42018100321) and was reported according to the Preferred Reporting Items for Systematic Reviews and Meta-Analyses (PRISMA)^13^.

### Data sources and searches

We searched Embase (S1 Appendix), Medline, Cochrane Library and ClinicalTrials.gov from inception to November 23^rd^, 2017. We included randomized controlled trials (RCTs) that compared anticoagulant interventions for SPAF in adults, whether administered alone or as care bundles. No language restriction was applied.

### Study selection

Trials with mixed population (e.g. AF, venous thromboembolism and etc) were included in this review if more than 50% of AF patients were represented. This is to ensure that all interventions were incorporated into our network since most trials investigating warfarin care bundles were conducted in a mixed population. Only trials investigating interventions with approved dosing regimen or indication for SPAF were included. Trials with participants only eligible for parenteral anticoagulation or with an INR target outside 2–3 range were excluded. Furthermore, trials assessing patients undergoing catheter ablation, cardioversion, or recent surgery such as hip or knee arthroplasty were excluded. Reference lists of relevant studies were also screened.

### Data extraction and quality assessment

Three investigators (SSN, PD and KK) independently screened the titles and abstracts of retrieved citations to identify potentially relevant studies. Full articles were evaluated if a decision could not be made based on the titles and abstracts. When studies have compared more than two interventions, only interventions meeting the pre-specified criteria were included in the review if at least two interventions remained in the study.

The following data was extracted: study characteristics, patients’ characteristics, outcomes, and other relevant findings. The Cochrane Collaboration’s risk of bias (ROB) tool^14^ was used to assess risk of bias. Data extraction and risk of bias assessments were carried out by one reviewer (SSN) and cross-checked by two other reviewers (PD and KK). Any discrepancies were resolved by consensus or by arbitration to a third reviewer (NC) where necessary.

### Type of interventions

Interventions included were antiplatelet (e.g. aspirin of any doses, clopidogrel) and anticoagulant therapy (e.g. warfarin and NOACs). Warfarin was further categorized, either as usual warfarin care or as care bundles. Usual warfarin care consisted of warfarin therapy alone and may have been delivered either in hospitals, primary care or anticoagulation clinics. Anticoagulation clinics were included in the usual warfarin care because most RCTs were multicentre trials, whereby some patients in the warfarin arms may have been monitored in anticoagulation clinics while some in primary care by general physician. As a result, categorizing anticoagulation clinics as a standalone intervention is not feasible and was included as part of the usual warfarin care. Warfarin care bundles were the combination of several interventions performed collectively to improve the quality of warfarin care such as genotype-guided warfarin dosing, patient’s self-testing (PST) or patient’s self-management (PSM) of warfarin or LAAC procedure (e.g. insertion of Watchman device) with temporary warfarin use. These anticoagulant interventions are described in detail in S2 Appendix.

This review focused on four NOACs; one direct thrombin inhibitor; dabigatran and three factor Xa inhibitors; apixaban, rivaroxaban and edoxaban. Other NOACs were excluded and reasons for exclusions are listed in S2 Appendix. Dabigatran and edoxaban were analysed as two separate doses (150 mg or 110 mg for dabigatran; 60 mg or 30 mg for edoxaban) as patients were equally randomized to both doses in the main trials^10,11^. However, rivaroxaban and apixaban were not studied as separate doses due to the absence of equal randomization to respective doses. Patients were predominantly treated with either rivaroxaban 20 mg or apixaban 5 mg in the respective trials^12,15^ and were only adjusted to the lower dose if old age, low body weight or deterioration of renal function.

### Outcomes

The primary efficacy outcomes were stroke or systemic embolism and all-cause mortality while the primary safety outcome was major bleeding. Major bleeding was defined according to Bleeding Academic Research Consortium (BARC) type 3–5^16^ and “compatible definitions” if they could be standardized based on BARC type 3–5 criteria (details of compatibility criteria in S4 Appendix).

Secondary outcomes were ischemic stroke, clinically relevant non-major bleeding (CRNMB), intracranial bleeding (including haemorrhagic stroke, intraparenchymal, subdural, epidural and subarachnoid haemorrhages), gastrointestinal bleeding and myocardial infarction. The risk-benefit balance of anticoagulant interventions was also investigated by incorporating efficacy and safety outcomes using two-dimensional plot and clustering methods to rank these interventions.

### Quality of evidence

The quality of evidence from direct and indirect comparisons were assessed by using GRADEpro GDT software online version (GRADE Working Group, McMaster University, Hamilton, ON, Canada)^17^. There were four levels of quality of evidence: high, moderate, low and very low^18,19^.

### Data synthesis and statistical analysis

The relative intervention effects (risk ratio [RR]) along with 95% confidence interval (CI) were estimated for individual studies. Pairwise meta-analysis was used to pool RRs using random-effects model^20^. Heterogeneity was assessed using Cochrane Q test and I^2^ statistics^21^. A network meta-analysis with consistency model was conducted to compare all interventions using direct and indirect evidence^22,23^. Usual warfarin care was used as the common comparator in the network model. Network inconsistency was evaluated using global inconsistency test by fitting design-by-treatment in the inconsistency model^24^. If inconsistency was detected, we then used loop-specific and node-splitting methods to identify the source of inconsistency. The comparison-adjusted funnel plots were used to analyse publication bias^25^. To rank the intervention hierarchy in the network meta-analysis, surface under the cumulative ranking curves (SUCRA) were estimated^26^.

Sensitivity analysis was performed on different major bleeding definitions. Additionally, we also performed sensitivity analyses by excluding studies conducted prior to year 2006. This is to reduce the heterogeneity in usual warfarin care definition, by assuming that most countries have adopted anticoagulation clinics as part of their warfarin care management from year 2006 onwards^27^. Other sensitivity analyses were the omission of trials with mixed population, small trials (<25^th^ percentile), trials with serious-to-critical risk of bias. All analyses were done in Stata Version 14.0 using self-programmed Stata routines for network meta-analysis^25^. A p-value of less than 0.05 was considered statistically significant.

## Results

### Characteristics of the included studies

We identified 3,008 records, of which 189 potentially eligible articles were reviewed in full text. Of these articles, 152 were excluded, due to the lack of reporting on the outcomes of interest (n = 19), compared irrelevant interventions (n = 31) or population not of interest (n = 49), being non-RCTs or unrelated subgroup analyses (n = 14), and other reasons (n = 21), leaving 37 studies for inclusion in our review. The PRISMA flow diagram demonstrating process of electronic searching is presented in S1 Appendix.

A total of 37 studies, involving 100,142 patients^9–12,15,28–68^ were assessed in our network meta-analysis. The mean age of patients was 69.9 ± 5.7 years. Mean TTR for usual warfarin care was 61.1 ± 8.9% while the mean TTR for warfarin care bundles was 68.9 ± 5.8%.

Eight studies examined warfarin care bundles: two studies^48,67^ on PST, three studies^49,51,52^ on PSM, two studies^65,66^ on the insertion of Watchman device and one study^33^ on genotype-guided warfarin dosing. Fifteen studies^9–12,15,34–43^ explored one of the following NOACs: dabigatran^10,34,36^, rivaroxaban^12,37–39,41^, apixaban^9,15,40^ and edoxaban^11,35,42,43^. The remaining 14 studies^47,50,53–64^ compared warfarin with either antiplatelet therapy or placebo/control. Twenty-three studies^9–12,15,34–38,40,42,43,47,49,51,52,54,57,62,64–66^ were industry-sponsored, including all those that examined NOACs except one study^39^. Sponsor details were not reported in two studies^41,50^. Other characteristics of the included studies are summarized in S3 Appendix.

### Risk of bias of included studies

The risks of bias among included studies are presented in S5 Appendix. Most studies were judged to be at low or unclear risk of bias for sequence generation and allocation concealment. However, high risk of bias was found in the blinding of participants and staffs in 26 studies^10,33,34,36,37,40–43,49–64,67^ as most studies were conducted as open-label. Majority of studies were judged to be at low or unclear risk of bias for blinding of outcome assessment, incomplete outcome data and selective reporting.

### Efficacy and safety results

**Figure 1:**
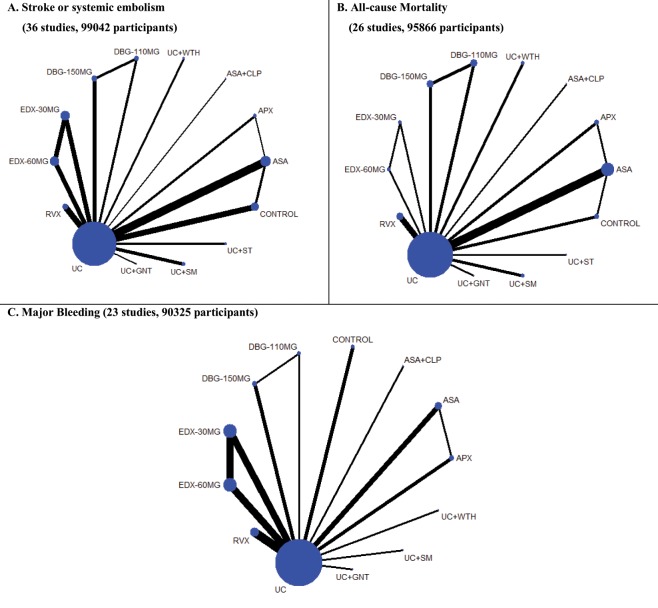
Network plots of eligible comparisons for primary efficacy and safety outcomes. (**A**) Stroke or systemic embolism. (**B**) All-cause mortality. (**C**) Major bleeding. Line thickness is proportional to the number of patients that contributed to the comparisons. Abbreviations: APX, apixaban; ASA, aspirin; CLP, clopidogrel; DBG-110MG, dabigatran 110 mg; DBG-150MG, dabigatran 150 mg; EDX-30MG, edoxaban 30 mg; EDX-60MG, edoxaban 60 mg; GNT, genotype-guided warfarin dosing; RVX, rivaroxaban; SM: self-management of warfarin; ST: self-testing of warfarin; WTH: Watchman device.

A total of 14 interventions were included in the network: the direct comparisons for primary outcomes are shown in Fig. 1. Network maps for secondary outcomes are presented in S6 Appendix. Treatment effects estimated using direct meta-analysis are presented in S8 Appendix, without evidence of statistical heterogeneity, except in two pairwise comparisons (usual warfarin care vs. aspirin for stroke or systemic embolism outcome and usual warfarin care vs rivaroxaban for gastrointestinal bleeding outcome). Comparisons among all interventions, with usual warfarin care as the reference treatment for all outcomes are presented in S9 Appendix.

**Figure 2:**
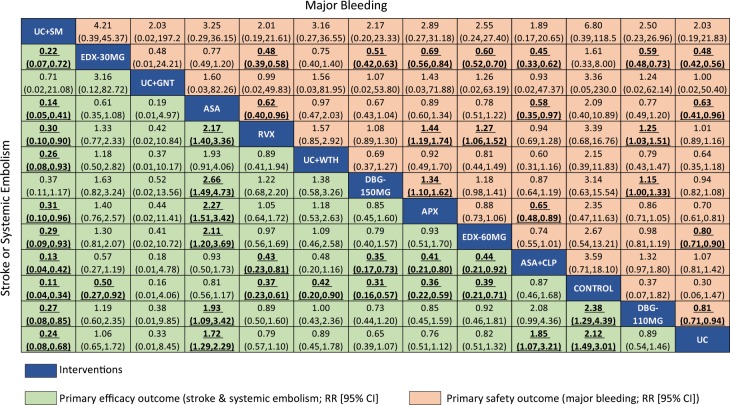
Network meta-analysis of primary efficacy (stroke or systemic embolism) and safety (major bleeding) outcomes. Interventions are ordered by ranking for stroke or systemic embolism. Results are the RRs (95% CI) from the network meta-analysis between the column-defining interventions and row-defining interventions. Comparisons should be read from left to right. Numbers in bold represent statistically significant results. UC + ST intervention was omitted as major bleeding outcome was not reported by the studies included in the analysis. Abbreviations: APX, apixaban; ASA, aspirin; CLP, clopidogrel; DBG-110MG, dabigatran 110 mg; DBG-150MG, dabigatran 150 mg; EDX-30MG, edoxaban 30 mg; EDX-60MG, edoxaban 60 mg; GNT, genotype-guided warfarin dosing; RVX, rivaroxaban; SM: self-management of warfarin; WTH: Watchman device.

All warfarin care bundles, apixaban, dabigatran 110 mg, dabigatran 150 mg, edoxaban 60 mg and rivaroxaban reduced the risk of stroke or systemic embolism when compared with usual warfarin care, as shown in Table 1 and Fig. 2. However, only PSM of warfarin care bundle showed significant reduction, with 76% risk reduction in stroke or systemic embolism (RR: 0.24, 95% CI 0.08–0.68). Conversely, antiplatelet therapy was associated with a significant increase in risk of stroke; RR 1.72 (95% CI 1.29–2.29) for single and 1.85 (1.07–3.21) for dual antiplatelet therapy, compared with usual warfarin care. Comparing among NOACS, the risk of stroke was higher with apixaban, rivaroxaban and edoxaban 60 mg when compared with dabigatran 150 mg, although these differences did not reach statistical significance.

**Table 1:** Network meta-analysis results of stroke, mortality, ischemic stroke and myocardial infarction outcomes in patients with AF.

	Primary Efficacy Outcomes		Secondary Outcomes	
	Stroke or SE	All-cause Mortality	Ischemic Stroke	Myocardial Infarction
**Comparisons with usual warfarin care**				
**Antiplatelet**				
Aspirin	**1.72 (1.29,2.29)**	1.07 (0.92,1.24)	**2.65 (1.89,3.71)**	1.04 (0.68,1.59)
Aspirin + Clopidogrel	**1.85 (1.07,3.21)**	1.00 (0.80,1.23)	**2.12 (1.47,3.05)**	1.55 (0.92,2.61)
**Warfarin Care Bundles**				
Genotype-guided warfarin dosing	0.33 (0.01,8.45)	2.51 (0.49,12.81)	N/A	N/A
PST	0.99 (0.51,1.93)	0.96 (0.78,1.19)	N/A	1.49 (0.83,2.70)
PSM	**0.24 (0.08,0.68)**	0.42 (0.17,1.04)	N/A	N/A
Watchman Device	0.89 (0.45,1.78)	**0.49 (0.28,0.86)**	1.36 (0.69,2.69)	N/A
**NOACs**				
Apixaban	0.76 (0.51,1.12)	**0.89 (0.80,0.98)**	0.93 (0.76,1.14)	0.88 (0.67,1.15)
Dabigatran 110 mg	0.89 (0.54,1.46)	0.92 (0.81,1.04)	1.12 (0.90,1.40)	1.36 (0.99,1.88)
Dabigatran 150 mg	0.65 (0.39,1.07)	0.89 (0.79,1.01)	**0.77 (0.61,0.99)**	**1.40 (1.01,1.92)**
Edoxaban 30 mg	1.06 (0.65,1.72)	**0.88 (0.80,0.96)**	**1.42 (1.20,1.67)**	1.21 (0.97,1.50)
Edoxaban 60 mg	0.82 (0.51,1.32)	0.92 (0.84,1.01)	1.00 (0.84,1.20)	0.95 (0.75,1.20)
Rivaroxaban	0.79 (0.57,1.10)	0.85 (0.71,1.01)	0.87 (0.72,1.05)	0.82 (0.63,1.06)
**Placebo (Control)**	**2.12 (1.49,3.01)**	1.11 (0.80,1.56)	**2.86 (1.83,4.49)**	1.00 (0.14,7.00)
**Comparisons among recommended doses of NOACs**				
Dabigatran 150 mg vs. Apixaban	0.85 (0.45,1.60)	1.00 (0.86,1.18)	0.83 (0.61,1.14)	**1.59 (1.05,2.41)**
Edoxaban 60 mg vs. Apixaban	1.08 (0.59,1.98)	1.04 (0.91,1.19)	1.08 (0.83,1.41)	1.08 (0.76,1.54)
Rivaroxaban vs. Apixaban	1.05 (0.64,1.72)	0.95 (0.78,1.17)	0.94 (0.71,1.24)	0.93 (0.64,1.35)
Edoxaban 60 mg vs. Dabigatran 150 mg	1.26 (0.64,2.50)	1.03 (0.89,1.21)	1.30 (0.96,1.76)	0.68 (0.46,1.01)
Rivaroxaban vs. Dabigatran 150 mg	1.22 (0.68,2.20)	0.95 (0.76,1.18)	1.13 (0.83,1.53)	**0.59 (0.39,0.88)**
Rivaroxaban vs. Edoxaban 60 mg	0.97 (0.56,1.69)	0.92 (0.75,1.12)	0.87 (0.67,1.13)	0.86 (0.61,1.22)
**Comparisons between NOACs with warfarin care bundles**				
Apixaban vs. Genotype-guided warfarin dosing	2.27 (0.09,58.54)	0.35 (0.07,1.81)	N/A	N/A
Dabigatran 150 mg vs. Genotype-guided warfarin dosing	1.93 (0.07,50.71)	0.35 (0.07,1.82)	N/A	N/A
Edoxaban 60 mg vs. Genotype-guided warfarin dosing	2.27 (0.09,58.54)	0.37 (0.07,1.88)	N/A	N/A
Rivaroxaban vs. Genotype-guided warfarin dosing	2.37 (0.09,60.75)	0.34 (0.07,1.73)	N/A	N/A
Apixaban vs. PST	0.76 (0.35,1.65)	0.92 (0.73,1.16)	N/A	0.59 (0.31,1.13)
Dabigatran 150 mg vs. PST	0.65 (0.28,1.50)	0.93 (0.72,1.18)	N/A	0.94 (0.48,1.83)
Edoxaban 60 mg vs. PST	0.82 (0.36,1.87)	0.96 (0.76,1.20)	N/A	0.64 (0.34,1.21)
Rivaroxaban vs. PST	0.80 (0.38,1.67)	0.88 (0.67,1.16)	N/A	0.55 (0.29,1.05)
Apixaban vs. PSM	**3.20 (1.04,9.88)**	2.12 (0.85,5.28)	N/A	N/A
Dabigatran 150 mg vs. PSM	2.74 (0.85,8.79)	2.12 (0.85,5.32)	N/A	N/A
Edoxaban 60 mg vs. PSM	**3.45 (1.08,11.03)**	2.20 (0.88,5.48)	N/A	N/A
Rivaroxaban vs. PSM	**3.35 (1.11,10.14)**	2.01 (0.80,5.09)	N/A	N/A
Apixaban vs. Watchman Device	0.85 (0.38,1.90)	**1.81 (1.03,3.19)**	0.68 (0.34,1.39)	N/A
Dabigatran 150 mg vs. Watchman Device	0.72 (0.31,1.71)	**1.81 (1.02,3.21)**	0.57 (0.28,1.18)	N/A
Edoxaban 60 mg vs. Watchman Device	0.91 (0.39,2.16)	**1.88 (1.07,3.30)**	0.74 (0.36,1.50)	N/A
Rivaroxaban vs. Watchman Device	0.89 (0.41,1.94)	1.72 (0.96,3.09)	0.64 (0.32,1.30)	N/A

PST: patient’s self-testing of warfarin; PSM: patient’s self-management of warfarin; SE: systemic embolism CRNMB: clinically relevant non-major bleeding; PST: patient’s self-testing of warfarin; PSM: patient’s self-management of warfarin; WTH: Watchman device. * Numbers in bold represent statistically significant results.

Three interventions significantly reduced the risk of all-cause mortality when compared with usual warfarin care: insertion of Watchman device (0.49, 0.28–0.86); apixaban (0.89, 0.80–0.98 and edoxaban 30 mg (0.88, 0.80–0.96). There was little evidence that the risk of all-cause mortality differed among NOACs. Our meta-analysis observed that there was a potential for an increased risk of myocardial infarction associated with the use of dabigatran 150 mg (1,40, 1.01–1.92) when compared with usual warfarin care. However, the increased trend may not be conclusive as the lower limit of the CI fell just above the null value 1.0, with a possible increased risk of myocardial infarction as high as 1% only.

For safety outcomes (Table 2), apixaban (0.70, 0.61–0.81), dabigatran 110 mg (0.81, 0.71–0.94), edoxaban 30 mg (0.48, 0.42–0.56), and edoxaban 60 mg (0.80, 0.71–0.90) significantly reduced the risk of major bleeding compared with usual warfarin care. The evidence for warfarin care bundles, especially genotype-guided warfarin dosing and PSM intervention in major bleeding outcome is generally weak due to the wide confidence intervals, corresponding to the small sample sizes of included trials. Among NOACs, the major bleeding risk with dabigatran 150 mg (1.34, 1.10–1.62) and rivaroxaban (1.44, 1.91–1.74) were significantly higher than apixaban.

**Table 2:** Network meta-analysis results of bleeding outcomes in patients with atrial fibrillation.

	Primary Safety Outcomes		Secondary Outcomes	
	Major Bleeding	Intracranial Bleeding	CRNMB	GI Bleeding
**Comparison with usual warfarin care**				
**Antiplatelet**				
Aspirin	**0.63 (0.41,0.96)**	0.65 (0.34,1.25)	**0.60 (0.44,0.83)**	0.48 (0.07,3.28)
Aspirin + Clopidogrel	1.07 (0.81,1.42)	0.52 (0.23,1.17)	N/A	N/A
**Warfarin Care Bundles**				
Genotype-guided warfarin dosing	1.00 (0.02,50.40)	N/A	N/A	0.74 (0.12,4.51)
PST	N/A	1.37 (0.51,3.64)	N/A	N/A
PSM	2.03 (0.19,21.83)	1.00 (0.14,7.29)	N/A	N/A
Watchman Device	0.64 (0.35,1.18)	**0.22 (0.06,0.76)**	N/A	N/A
**NOACs**				
Apixaban	**0.70 (0.61,0.81)**	**0.44 (0.28,0.70)**	**0.68 (0.59,0.79)**	0.62 (0.13,2.86)
Dabigatran 110 mg	**0.81 (0.71,0.94)**	**0.31 (0.18,0.55)**	0.51 (0.11,2.34)	1.11 (0.21,5.87)
Dabigatran 150 mg	0.94 (0.82,1.08)	**0.41 (0.24,0.70)**	1.13 (0.51,2.51)	1.50 (0.29,7.93)
Edoxaban 30 mg	**0.48 (0.42,0.56)**	**0.31 (0.19,0.52)**	**0.70 (0.65,0.75)**	0.68 (0.13,3.58)
Edoxaban 60 mg	**0.80 (0.71,0.90)**	**0.48 (0.30,0.78)**	**0.87 (0.82,0.94)**	1.22 (0.23,6.41)
Rivaroxaban	1.01 (0.89,1.16)	0.76 (0.53,1.08)	1.04 (0.97,1.11)	1.37 (0.42,4.43)
**Placebo (Control)**	0.30 (0.06,1.47)	0.57 (0.19,1.71)	0.33 (0.03,3.16)	0.23 (0.02,2.20)
**Comparisons among recommended doses of NOACs**				
Dabigatran 150 mg vs. Apixaban	**1.34 (1.10,1.62)**	0.93 (0.46,1.86)	1.65 (0.73,3.72)	2.42 (0.25,23.12)
Edoxaban 60 mg vs. Apixaban	1.14 (0.94,1.37)	1.09 (0.57,2.08)	**1.28 (1.09,1.51)**	1.97 (0.21,18.72)
Rivaroxaban vs. Apixaban	**1.44 (1.19,1.74)**	1.72 (0.96,3.07)	**1.52 (1.29,1.79)**	2.20 (0.30,16.04)
Edoxaban 60 mg vs. Dabigatran 150 mg	0.85 (0.71,1.02)	1.17 (0.57,2.40)	0.77 (0.35,1.73)	0.81 (0.08,8.50)
Rivaroxaban vs. Dabigatran 150 mg	1.08 (0.89,1.30)	1.85 (0.98,3.52)	0.92 (0.41,2.05)	0.91 (0.12,6.97)
Rivaroxaban vs. Edoxaban 60 mg	**1.27 (1.06,1.52)**	1.58 (0.86,2.89)	**1.19 (1.08,1.31)**	1.12 (0.15,8.55)
**Comparisons between NOACs with warfarin care bundles**				
Apixaban vs. Genotype-guided warfarin dosing	0.70 (0.01,35.20)	N/A	N/A	0.84 (0.08,9.02)
Dabigatran 150 mg vs. Genotype-guided warfarin dosing	0.93 (0.02,47.03)	N/A	N/A	2.04 (0.17,23.88)
Edoxaban 60 mg vs. Genotype-guided warfarin dosing	0.80 (0.02,40.01)	N/A	N/A	1.66 (0.14,19.33)
Rivaroxaban vs. Genotype-guided warfarin dosing	1.01 (0.02,50.75)	N/A	N/A	1.86 (0.21,16.10)
Apixaban vs. PST	N/A	**0.32 (0.11,0.95)**	N/A	N/A
Dabigatran 150 mg vs. PST	N/A	**0.30 (0.10,0.91)**	N/A	N/A
Edoxaban 60 mg vs. PST	N/A	0.35 (0.12,1.05)	N/A	N/A
Rivaroxaban vs. PST	N/A	0.56 (0.20,1.58)	N/A	N/A
Apixaban vs. PSM	0.35 (0.03,3.72)	0.44 (0.06,3.42)	N/A	N/A
Dabigatran 150 mg vs. PSM	0.46 (0.04,4.97)	0.41 (0.05,3.22)	N/A	N/A
Edoxaban 60 mg vs. PSM	0.39 (0.04,4.23)	0.48 (0.06,3.73)	N/A	N/A
Rivaroxaban vs. PSM	0.50 (0.05,5.36)	0.76 (0.10,5.75)	N/A	N/A
Apixaban vs. Watchman Device	1.09 (0.59,2.03)	2.01 (0.54,7.44)	N/A	N/A
Dabigatran 150 mg vs. Watchman Device	1.46 (0.79,2.71)	1.86 (0.48,7.11)	N/A	N/A
Edoxaban 60 mg vs. Watchman Device	1.24 (0.67,2.30)	2.18 (0.58,8.14)	N/A	N/A
Rivaroxaban vs. Watchman Device	1.57 (0.85,2.92)	3.45 (0.95,12.46)	N/A	N/A

PST: patient’s self-testing of warfarin; PSM: patient’s self-management of warfarin; SE: systemic embolism CRNMB: clinically relevant non-major bleeding; PST: patient’s self-testing of warfarin; PSM: patient’s self-management of warfarin; WTH: Watchman device. * Numbers in bold represent statistically significant results.

The risk of intracranial bleeding was significantly lower, with more than 50% relative risk reduction for all NOACs except rivaroxaban when compared with usual warfarin care. For gastrointestinal bleeding, the risk was higher with dabigatran 110 mg (1.11, 0.21–5.87), dabigatran 150 mg (1.50, 0.29–7.93), edoxaban 60 mg (1.22, 0.23–6.41) and rivaroxaban (1.37, 0.42–4.43) compared with usual warfarin care. On the contrary, the risk of CRNMB for single antiplatelet therapy (0.60, 0.44–0.83), apixaban (0.68, 0.59–0.79), edoxaban 30 mg (0.70, 0.65–0.75) and edoxaban 60 mg (0.87, 0.82–0.94) was significantly lower than usual warfarin care.

**Figure 3:**
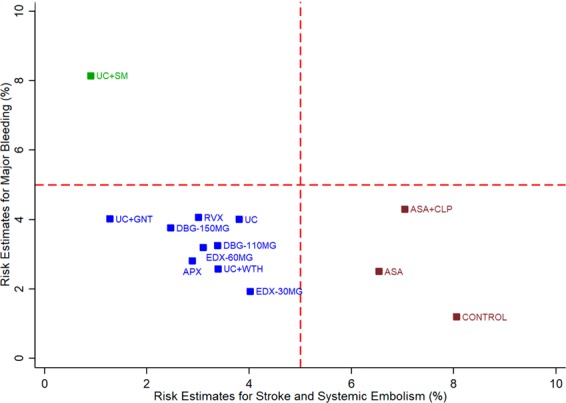
Cluster rank plot of risk estimates for stroke or systemic embolism and major bleeding. The risk estimates plot of patient’s INR self-testing intervention is omitted because the included studies for this intervention in network meta-analyses did not report on major bleeding outcome. The dashed line represents the different quadrants of the risk estimates. Abbreviations: APX, apixaban; ASA, aspirin; CLP, clopidogrel; DBG-110MG, dabigatran 110 mg; DBG-150MG, dabigatran 150 mg; EDX-30MG, edoxaban 30 mg; EDX-60MG, edoxaban 60 mg; GNT, genotype-guided warfarin dosing; RVX, rivaroxaban; SM: self-management of warfarin; WTH: Watchman device.

The cluster rank plot (Fig. 3) shows that warfarin care bundles except for PSM were clustered in the same quadrant (lower risk of stroke and major bleeding) as NOACs when balancing both the efficacy and safety outcomes. A global inconsistency test was performed and suggested no evidence of inconsistency for all outcomes (S7 Appendix). Therefore, the pooled estimates of all outcomes were based on consistency model. Comparison-adjusted funnel plots show no evidence of asymmetry (S13 Appendix).

In our sensitivity analysis (S12 Appendix), when studies conducted before year 2006 were excluded, only the RR of PSM intervention for stroke and all-cause mortality outcome increased while the findings for other interventions remain the same. The findings were also generally robust when studies with mixed population were excluded. Moreover, findings for major bleeding outcomes were also robust without significant changes in the treatment hierarchies when major bleeding definition was matched against ISTH definition instead of BARC 3–5 criteria.

The quality of direct evidence for all outcomes was generally rated as very low to moderate in most comparisons. When GRADE was applied to our network meta-analysis evidence, mortality and major bleeding outcome showed better rating on quality of evidence than for direct evidence in several comparisons. More details of the quality of evidence are presented in the S15 Appendix.

## Discussions

To our knowledge, this is the first study that comprehensively compared efficacy and safety of all available anticoagulant interventions. Our analysis help provide new important information by evaluating all interventions in one single network model. This information therefore provides clinicians and policy makers with a broad view of the whole landscape of SPAF interventions.

Our results confirm that NOACs have similar efficacy to usual warfarin for stroke prevention but offer some advantages through reduction in bleeding risk. Nevertheless, some of the warfarin care bundles including genotype-guided warfarin dosing, and insertion of Watchman device, (LAAC) performed well in comparison to NOACs based on the cluster rank plot, which incorporate both efficacy and safety into one aggregate. Although there were no differences in primary stroke or major bleeding outcomes for LAAC procedure when compared with usual warfarin care, it is important to note that these are broad non-specific outcomes which includes numerous other outcomes such as ischemic stroke or intraparenchymal, subdural, epidural or subarachnoid haemorrhage, which may mask the beneficial effect of LAAC on haemorrhagic stroke and intracranial bleeding, respectively. Intracranial bleeding or haemorrhagic stroke is often associated with poor prognosis and high case fatality rates. By reducing the incidence of these events, the benefits of LAAC may result in lower overall mortality as reflected in our study findings. Moreover, the mortality reduction benefits of LAAC might be associated with the ability to discontinue long-term anticoagulation which is often associated with high intracranial bleeding risk. Therefore, substituting the use of long-term anticoagulation with other alternatives such as LAAC procedure may reduce mortality and provide favourable long-term survival benefits.

Conversely, PSM performed extremely well in stroke risk reduction but with increased risk of major bleeding. The risk of bleeding seen with PSM may need to be interpreted with caution since most trials of PSM were performed in mixed population where bleeding rates may be higher than that of AF patients. For PST, there appears to be no additional benefit on stroke reduction while most trials did not report on the rates of major bleeding. Therefore, we were unable to evaluate PST using cluster rank plot.

It is important to highlight that our findings show that warfarin care bundles are associated with improved quality of anticoagulation control compared with usual warfarin care (TTR of 68.9% vs. 61.1%, respectively). Since the efficacy and safety of warfarin is dependent on the quality of anticoagulation control, it is therefore plausible that warfarin performance can be as good as NOACs if TTR is high. Our study showed that warfarin care bundles achieved a mean TTR of 68.9%. As a result, this bundle care may offer a promising alternative instead of switching to NOACs since comparable benefits can be achieved at a possible lower cost. Nevertheless, we caution readers to consider this result definitive. The quality of evidence for majority warfarin care bundles except for the insertion of Watchman device, was generally rated as very low to low quality due to imprecision, presence of mixed population and wide confidence interval of pooled risk estimates. If possible, this finding should be confirmed in a large, randomized, controlled trial and other types of high-quality researches in the future.

Despite not being definitive, this finding may be an important information for low-income countries where there is still limited accessibility to NOACs in healthcare reimbursement scheme. Nonetheless, additional information such as cost-effectiveness and technical implementation considerations (e.g. expertise, training, laboratory, facilities) need to be taken into consideration when formulating national or institutional policy decisions.

Our findings also strengthen current recommendation that oral anticoagulants, such as warfarin or NOACs, are far superior to single or dual antiplatelet therapy in preventing stroke in AF patients. Thus, antiplatelet therapy may no longer have a role for stroke prevention in AF patients as risks without considerable benefits is a poor trade-off.

### Strength and weakness of this study

The strength of our study includes a comprehensive analysis of NOACs and warfarin care bundles in a single network. Previous meta-analyses^69–72^ compared NOACs with usual warfarin care only, without addressing warfarin care bundles and this may potentially favour NOACs. The main results in our NMA are also presented by simultaneous clustered ranking of efficacy and safety outcomes, allowing us to explore the intervention that has the best balance of both benefits and risks.

Our review has several limitations. Our analyses were restricted by the modest amount of data in the included studies. Only a few studies reported outcomes such as gastrointestinal bleeding, intracranial bleeding and myocardial infarction and most had few or zero events. Such missing information may encumber a thorough comparison of all interventions for each individual outcome and results for these outcomes should be interpreted with caution. Second, trials with mixed population (provided they included more than 50% of AF patients) were included in this review. As such, readers should be prudent when interpreting the findings as heterogeneity in patients’ characteristics, thromboembolic risk as well as haemorrhagic risk may exist among the study populations. Moreover, LAAC is also an invasive procedure which is usually reserved for the frail population with higher bleeding and thromboembolic risk.7 Third, there is also notable heterogeneity on the types of AF (e.g. persistent, paroxysmal or permanent). However, the number of studies that addressed the types of AF were small and hence, subgroup analyses could not be conducted to account for this heterogeneity. Fourth, the heterogeneity of major bleeding is another concern due to diverse classification of bleeding events (e.g. major, life-threatening and minor) among trials in this research field. To minimize heterogeneity in our study, the definition of major bleeding reported in each study was matched against standardized bleeding end-point definition adopted by BARC^16^. BARC definition was selected because it is the most updated bleeding definition and has been validated against other bleeding definitions^73,74^. We included studies with reported bleeding definition that was compatible to BARC bleeding type 3–5 criteria into our quantitative analysis. We also performed sensitivity analysis on different major bleeding definition (e.g. ISTH) to assess the robustness of our conclusions. Fifth, we did not analyse apixaban and rivaroxaban separately as high or low doses due to lack of randomization of both doses in the main trials^9,12^. Finally, follow-up duration in several studies, especially those investigating warfarin care bundles were relatively too short to draw definitive conclusion for long-term mortality outcome.

## Conclusions

In summary, our analysis suggests that NOACs appear to be at least equivalent to usual warfarin care for SPAF and some NOACs carry a reduced risk of bleeding. However, the favourable benefits of NOACs decreases when compared to warfarin care bundles. Warfarin care bundles improve the quality of warfarin control as expressed by high TTR value (≥65%). Warfarin care bundles such as PSM and insertion of Watchman device offer the highest level of efficacy in terms of stroke and mortality reduction, respectively. These findings should be considered during the decision-making process, on either adoption of NOACs into the healthcare system or investment in improving warfarin therapy by adopting care bundles. However, more trials comparing these care bundles with NOACs are needed in the future to overcome the need for indirect comparisons and to better understand the role of these interventions in clinical setting.

## Supplementary information

Supplementary Appendix.

PRISMA Checklist.

## Data Availability

All data generated or analysed during this study are included in this published article (and its Supplementary Information files).

## References

[1] Risk factors for stroke and efficacy of antithrombotic therapy in atrial fibrillation. Analysis of pooled data from five randomized controlled trials. *Archives of internal medicine* **154**, 1449–1457 (1994). 8018000

[2] Gadisseur AP, Kaptein AA, Breukink-Engbers WG, van der Meer FJ, Rosendaal FR (2004). Patient self-management of oral anticoagulant care vs. management by specialized anticoagulation clinics: positive effects on quality of life. Journal of thrombosis and haemostasis: JTH.

[3] Ogilvie IM, Newton N, Welner SA, Cowell W, Lip GY (2010). Underuse of oral anticoagulants in atrial fibrillation: a systematic review. The American journal of medicine.

[4] Resar, R., Griffin, F. A., Haraden, C. & Nolan, T. W. *Using Care Bundles to Improve Health Care Quality*., www.IHI.org (2012).

[5] Taborski U, Muller-Berghaus G (1999). State-of-the-art patient self-management for control of oral anticoagulation. Seminars in thrombosis and hemostasis.

[6] Mekaj YH, Mekaj AY, Duci SB, Miftari EI (2015). New oral anticoagulants: their advantages and disadvantages compared with vitamin K antagonists in the prevention and treatment of patients with thromboembolic events. Therapeutics and clinical risk management.

[7] Gulseth MP (2011). Dabigatran etexilate in clinical practice: confronting challenges to improve safety and effectiveness. Pharmacotherapy.

[8] National Insitute for Health and Clinical Excellence. Atrial fibrillation: the management of atrial fibrillation (CG180), https://www.nice.org.uk/guidance/cg180 (2014). 25340239

[9] Connolly SJ (2011). Apixaban in patients with atrial fibrillation. The New England journal of medicine.

[10] Connolly SJ (2009). Dabigatran versus warfarin in patients with atrial fibrillation. The New England journal of medicine.

[11] Giugliano RP (2013). Edoxaban versus warfarin in patients with atrial fibrillation. The New England journal of medicine.

[12] Patel MR (2011). Rivaroxaban versus warfarin in nonvalvular atrial fibrillation. The New England journal of medicine.

[13] Hutton B (2015). The PRISMA extension statement for reporting of systematic reviews incorporating network meta-analyses of health care interventions: checklist and explanations. Annals of internal medicine.

[14] Higgins JP (2011). The Cochrane Collaboration’s tool for assessing risk of bias in randomised trials. BMJ (Clinical research ed.).

[15] Granger CB (2011). Apixaban versus warfarin in patients with atrial fibrillation. The New England journal of medicine.

[16] Mehran R (2011). Standardized bleeding definitions for cardiovascular clinical trials: a consensus report from the Bleeding Academic Research Consortium. Circulation.

[17] GRADEpro GDT. https://gradepro.org/ (accessed Jun 9, 2018).

[18] Balshem H (2011). GRADE guidelines: 3. Rating the quality of evidence. Journal of clinical epidemiology.

[19] Puhan MA (2014). A GRADE Working Group approach for rating the quality of treatment effect estimates from network meta-analysis. BMJ (Clinical research ed.).

[20] DerSimonian R, Laird N (1986). Meta-analysis in clinical trials. Controlled clinical trials.

[21] Higgins JP, Thompson SG, Deeks JJ, Altman DG (2003). Measuring inconsistency in meta-analyses. BMJ (Clinical research ed.).

[22] Caldwell DM, Ades AE, Higgins JP (2005). Simultaneous comparison of multiple treatments: combining direct and indirect evidence. BMJ (Clinical research ed.).

[23] Lu G, Ades AE (2004). Combination of direct and indirect evidence in mixed treatment comparisons. Statistics in medicine.

[24] Dias S, Welton NJ, Caldwell DM, Ades AE (2010). Checking consistency in mixed treatment comparison meta-analysis. Statistics in medicine.

[25] Chaimani A, Higgins JP, Mavridis D, Spyridonos P, Salanti G (2013). Graphical tools for network meta-analysis in STATA. PloS one.

[26] Salanti G, Ades AE, Ioannidis JP (2011). Graphical methods and numerical summaries for presenting results from multiple-treatment meta-analysis: an overview and tutorial. Journal of clinical epidemiology.

[27] Pengo V, Pegoraro C, Cucchini U, Iliceto S (2006). Worldwide management of oral anticoagulant therapy: the ISAM study. Journal of thrombosis and thrombolysis.

[28] Burmester JK (2011). A randomized controlled trial of genotype-based Coumadin initiation. Genetics in medicine: official journal of the American College of Medical Genetics.

[29] Caraco Y, Blotnick S, Muszkat M (2008). CYP2C9 genotype-guided warfarin prescribing enhances the efficacy and safety of anticoagulation: a prospective randomized controlled study. Clinical pharmacology and therapeutics.

[30] Hillman MA (2005). A prospective, randomized pilot trial of model-based warfarin dose initiation using CYP2C9 genotype and clinical data. Clinical medicine & research.

[31] Jonas DE (2013). Impact of genotype-guided dosing on anticoagulation visits for adults starting warfarin: a randomized controlled trial. Pharmacogenomics.

[32] Kimmel SE (2013). A pharmacogenetic versus a clinical algorithm for warfarin dosing. The New England journal of medicine.

[33] Pirmohamed M (2013). A randomized trial of genotype-guided dosing of warfarin. The New England journal of medicine.

[34] (https://ClinicalTrials.gov/show/NCT01136408).

[35] Chung N (2011). Safety of edoxaban, an oral factor Xa inhibitor, in Asian patients with non-valvular atrial fibrillation. Thrombosis and haemostasis.

[36] Ezekowitz MD (2007). Dabigatran with or without concomitant aspirin compared with warfarin alone in patients with nonvalvular atrial fibrillation (PETRO Study). The American journal of cardiology.

[37] Hong KS (2017). Rivaroxaban vs Warfarin Sodium in the Ultra-Early Period After Atrial Fibrillation-Related Mild Ischemic Stroke: A Randomized Clinical Trial. JAMA neurology.

[38] Hori M (2012). Rivaroxaban vs. warfarin in Japanese patients with atrial fibrillation - the J-ROCKET AF study. Circulation journal: official journal of the Japanese Circulation Society.

[39] Mao L, Li C, Li T, Yuan K (2014). Prevention of stroke and systemic embolism with rivaroxaban compared with warfarin in Chinese patients with atrial fibrillation. Vascular.

[40] Ogawa S, Shinohara Y, Kanmuri K (2011). Safety and efficacy of the oral direct factor xa inhibitor apixaban in Japanese patients with non-valvular atrial fibrillation. -The ARISTOTLE-J study. Circulation journal: official journal of the Japanese Circulation Society.

[41] Shosa RI, Ibrahim OM, Setiha ME, Abdelwahab AA (2017). The Efficacy and Safety of Rivaroxaban as an Alternative to Warfarin for the Prevention of Thromboembolism in Patients with Atrial Fibrillation. Int J Pharm Sci Rev Res.

[42] Weitz JI (2010). Randomised, parallel-group, multicentre, multinational phase 2 study comparing edoxaban, an oral factor Xa inhibitor, with warfarin for stroke prevention in patients with atrial fibrillation. Thrombosis and haemostasis.

[43] Yamashita T (2012). Randomized, multicenter, warfarin-controlled phase II study of edoxaban in Japanese patients with non-valvular atrial fibrillation. Circulation journal: official journal of the Japanese Circulation Society.

[44] Beyth RJ, Quinn L, Landefeld CS (2000). A multicomponent intervention to prevent major bleeding complications in older patients receiving warfarin. A randomized, controlled trial. Annals of internal medicine.

[45] Cromheecke ME (2000). Oral anticoagulation self-management and management by a specialist anticoagulation clinic: a randomised cross-over comparison. Lancet (London, England).

[46] Dignan R, Keech AC, Gebski VJ, Mann KP, Hughes CF (2013). Is home warfarin self-management effective? Results of the randomised Self-Management of Anticoagulation Research Trial. International journal of cardiology.

[47] Lavitola Pde L (2010). Warfarin or aspirin in embolism prevention in patients with mitral valvulopathy and atrial fibrillation. Arquivos brasileiros de cardiologia.

[48] Matchar DB (2010). Effect of home testing of international normalized ratio on clinical events. The New England journal of medicine.

[49] Menendez-Jandula B (2005). Comparing self-management of oral anticoagulant therapy with clinic management: a randomized trial. Annals of internal medicine.

[50] Rash A (2007). A randomised controlled trial of warfarin versus aspirin for stroke prevention in octogenarians with atrial fibrillation (WASPO). Age and ageing.

[51] Verret L (2012). Impact of a pharmacist-led warfarin self-management program on quality of life and anticoagulation control: a randomized trial. Pharmacotherapy.

[52] Voller H (2005). Self-management of oral anticoagulation in nonvalvular atrial fibrillation (SMAAF study). Zeitschrift fur Kardiologie.

[53] Stroke Prevention in Atrial Fibrillation Study (1991). Final results. Circulation.

[54] Secondary prevention in non-rheumatic atrial fibrillation after transient ischaemic attack or minor stroke. EAFT (European Atrial Fibrillation Trial) Study Group. *Lancet (London, England)* **342**, 1255–1262 (1993). 7901582

[55] Warfarin versus aspirin for prevention of thromboembolism in atrial fibrillation: Stroke Prevention in Atrial Fibrillation II Study. *Lancet (London, England)* **343**, 687–691 (1994). 7907677

[56] Chen KP (2012). Anticoagulation therapy in Chinese patients with non-valvular atrial fibrillation: a prospective, multi-center, randomized, controlled study. Chinese medical journal.

[57] Connolly S (2006). Clopidogrel plus aspirin versus oral anticoagulation for atrial fibrillation in the Atrial fibrillation Clopidogrel Trial with Irbesartan for prevention of Vascular Events (ACTIVE W): a randomised controlled trial. Lancet (London, England).

[58] Connolly SJ (1991). Canadian Atrial Fibrillation Anticoagulation (CAFA) Study. Journal of the American College of Cardiology.

[59] Gullov AL (1998). Fixed minidose warfarin and aspirin alone and in combination vs adjusted-dose warfarin for stroke prevention in atrial fibrillation: Second Copenhagen Atrial Fibrillation, Aspirin, and Anticoagulation Study. Archives of internal medicine.

[60] Liu X (2014). Warfarin compared with aspirin for older Chinese patients with stable coronary heart diseases and atrial fibrillation complications. International journal of clinical pharmacology and therapeutics.

[61] Mant J (2007). Warfarin versus aspirin for stroke prevention in an elderly community population with atrial fibrillation (the Birmingham Atrial Fibrillation Treatment of the Aged Study, BAFTA): a randomised controlled trial. Lancet (London, England).

[62] Petersen P, Boysen G, Godtfredsen J, Andersen ED, Andersen B (1989). Placebo-controlled, randomised trial of warfarin and aspirin for prevention of thromboembolic complications in chronic atrial fibrillation. The Copenhagen AFASAK study. Lancet (London, England).

[63] Sato H (2006). Low-dose aspirin for prevention of stroke in low-risk patients with atrial fibrillation: Japan Atrial Fibrillation Stroke Trial. Stroke.

[64] Singer DE (1990). The effect of low-dose warfarin on the risk of stroke in patients with nonrheumatic atrial fibrillation. The New England journal of medicine.

[65] Holmes DR (2014). Prospective randomized evaluation of the Watchman Left Atrial Appendage Closure device in patients with atrial fibrillation versus long-term warfarin therapy: the PREVAIL trial. Journal of the American College of Cardiology.

[66] Reddy VY (2014). Percutaneous left atrial appendage closure vs warfarin for atrial fibrillation: a randomized clinical trial. Jama.

[67] Khan TI, Kamali F, Kesteven P, Avery P, Wynne H (2004). The value of education and self-monitoring in the management of warfarin therapy in older patients with unstable control of anticoagulation. British journal of haematology.

[68] Watzke HH, Forberg E, Svolba G, Jimenez-Boj E, Krinninger B (2000). A prospective controlled trial comparing weekly self-testing and self-dosing with the standard management of patients on stable oral anticoagulation. Thrombosis and haemostasis.

[69] Cameron C (2014). Systematic review and network meta-analysis comparing antithrombotic agents for the prevention of stroke and major bleeding in patients with atrial fibrillation. BMJ open.

[70] Lopez-Lopez JA (2017). Oral anticoagulants for prevention of stroke in atrial fibrillation: systematic review, network meta-analysis, and cost effectiveness analysis. BMJ (Clinical research ed.).

[71] Tawfik A (2016). Systematic review and network meta-analysis of stroke prevention treatments in patients with atrial fibrillation. Clinical pharmacology: advances and applications.

[72] Tereshchenko, L. G., Henrikson, C. A., Cigarroa, J. & Steinberg, J. S. Comparative Effectiveness of Interventions for Stroke Prevention in Atrial Fibrillation: A Network Meta-Analysis. *Journal of the American Heart Association* **5**, 10.1161/jaha.116.003206 (2016). 10.1161/JAHA.116.003206 PMC4889191 27207998

[73] Kikkert WJ (2014). The prognostic value of bleeding academic research consortium (BARC)-defined bleeding complications in ST-segment elevation myocardial infarction: a comparison with the TIMI (Thrombolysis In Myocardial Infarction), GUSTO (Global Utilization of Streptokinase and Tissue Plasminogen Activator for Occluded Coronary Arteries), and ISTH (International Society on Thrombosis and Haemostasis) bleeding classifications. Journal of the American College of Cardiology.

[74] Vranckx P (2014). Prospective validation of the Bleeding Academic Research Consortium classification in the all-comer PRODIGY trial. European heart journal.

