# Supplementary material for: Comparative efficacy and safety of warfarin care bundles and novel oral anticoagulants in patients with atrial fibrillation: a systematic review and network meta-analysis
Source: Sci Rep. 2020 Jan 20;10:662. doi: 10.1038/s41598-019-57370-2 (PMC6971267; doi:10.1038/s41598-019-57370-2)
Supplement: Supplementary file 1 — Supplementary Appendix. [file 41598_2019_57370_MOESM1_ESM.pdf]

Supplementary Appendix

Comparative efficacy and safety of warfarin care bundles and novel oral anticoagulants in patients with atrial fibrillation: a systematic review and network meta-analysis

Siok Shen Ng[1-3], Nai Ming Lai[4], Surakit Nathisuwan[5], Nowrozy Kamar Jahan[2], Piyameth Dilokthornsakul[6], Khachen Kongpakwattana[1,2], William Hollingworth[7], Nathorn Chaiyakunapruk[1,8,9]

[1]*School of Pharmacy, Monash University Malaysia, Bandar Sunway, Malaysia;*

[2]*Jeffrey Cheah School of Medicine and Health Sciences, Monash University Malaysia, Bandar Sunway, Malaysia;*

[3]*Department of Pharmacy, Hospital Melaka, Malaysia;*

[4]*School of Medicine, Taylor's University Malaysia, Selangor, Malaysia;*

[5]*Clinical Pharmacy Division, Department of Pharmacy, Faculty of Pharmacy, Mahidol University, Bangkok, Thailand;*

[6]*Center of Pharmaceutical Outcomes Research (CPOR), Department of Pharmacy Practice, Faculty of Pharmaceutical Sciences, Naresuan University, Phitsanulok, Thailand;*

[7]*Department of Population Health Sciences, Bristol Medical School, University of Bristol, Bristol, BS8 1NU, UK;*

[8] *College of Pharmacy, University of Utah, Salt Lake City, Utah, USA;*

[9]*Asian Centre for Evidence Synthesis in Population, Implementation and Clinical Outcomes (PICO), Health and Well-being Cluster, Global Asia in the 21st Century (GA21) Platform, Monash University Malaysia, Bandar Sunway, Selangor, Malaysia*

Correspondence to:

Nathorn Chaiyakunapruk, PharmD, PhD

College of Pharmacy,

University of Utah,

Salt Lake City,

Utah, USA

nathorn.chaiyakunapruk@utah.edu

Table of Contents

S1 Appendix: 5

eTable 1.1. Embase Search Strategy 5

eFigure 1.1. PRISMA Flow Diagram 6

S2 Appendix: Interventions 7

eTable 2.1: General Characteristics of Interventions 7

eFigure 2.1: Graphic Representation of Anticoagulant Interventions for Stroke Prevention in Patients with Atrial Fibrillation 8

eTable 2.2: Detailed Description of interventions 9

S3 Appendix: Characteristics of Included Studies 11

eTable 3.1: Descriptions of Included Studies 11

eTable 3.2: Descriptions of Participants of Included Studies 14

eTable 3.5: Details of Reported Concurrent Medications Received in the Included Studies 17

S4 Appendix: Matching of Major Bleeding Definitions 19

eTable 4.1: Major Bleeding Definitions of Each Study and Compatibility with BARC 3-5, TIMI Major, GUSTO severe and ISTH Major Bleeding Definitions 20

S5 Appendix: Risk of Bias Assessment 24

eFigure 5.1: Risk of Bias Graph: review authors' judgements about each risk of bias item presented as percentages for studies including in network analyses (37 studies) 24

eTable 5.1: List of interventions examined by included studies for stroke prevention in AF patients 24

eFigure 5.2: Risk of Bias Summary: review authors' judgements about each risk of bias item for each included study 25

S6 Appendix: Network Treatment of Comparison 27

eFigure 6.1: Network Map of Treatment Comparisons for Secondary Outcomes 27

S7 Appendix: Assessment of Inconsistency 28

eTable 7.1: Evaluation of the global inconsistency in network using the 'design-by-treatment' interaction model for each outcome. 28

S8 Appendix: Results of meta-analyses of direct comparisons of intervention options 29

eTable 8.1: Pairwise meta-analysis risk ratio (and 95% CI) for all dichotomous outcomes 29

S9 Appendix: Results of Network Meta-analysis 35

eTable 9.1: Results of network meta-analysis of interventions options on stroke or systemic embolism 36

eFigure 9.1: Network estimated risk ratios (95% confidence intervals) of intervention options on stroke or systemic embolism 37

eTable 9.2: Results of network meta-analysis of interventions options on major bleeding 38

eFigure 9.2: Network estimated risk ratios (95% confidence intervals) of intervention options on major bleeding 39

eTable 9.3: Results of network meta-analysis of interventions options on all-cause mortality ... 40
eFigure 9.3: Network estimated risk ratios (95% confidence intervals) of intervention options on all-cause mortality ... 41
eTable 9.4: Results of network meta-analysis of interventions options on ischemic stroke ... 42
eFigure 9.4: Network estimated risk ratios (95% confidence intervals) of intervention options on ischemic stroke ... 43
eTable 9.5: Results of network meta-analysis of interventions options on clinically relevant non-major bleeding ... 44
eFigure 9.5: Network estimated risk ratios (95% confidence intervals) of intervention options on clinically relevant non-major bleeding ... 45
eTable 9.6: Results of network meta-analysis of interventions options on intracranial bleeding ... 46
eFigure 9.6: Network estimated risk ratios (95% confidence intervals) of intervention options on intracranial bleeding ... 47
eTable 9.7: Results of network meta-analysis of interventions options on gastrointestinal bleeding ... 48
eFigure 9.7: Network estimated risk ratios (95% confidence intervals) of intervention options on gastrointestinal bleeding ... 49
eTable 9.8: Results of network meta-analysis of interventions options on myocardial infarction ... 50
eFigure 9.8: Network estimated risk ratios (95% confidence intervals) of intervention options on myocardial infarction ... 51
S10 Appendix: Treatment ranking and surface under the cumulative ranking curves (SUCRA) 52
eFigure 10.1: SUCRA ranking curve for stroke or systemic embolism ... 52
eFigure 10.2: SUCRA ranking curve for major bleeding ... 53
eFigure 10.3: SUCRA ranking curve for all-cause mortality ... 54
eFigure 10.4: SUCRA ranking curve for ischemic stroke ... 55
eFigure 10.5: SUCRA ranking curve for clinically relevant non-major bleeding ... 56
eFigure 10.6: SUCRA ranking curve for intracranial bleeding ... 57
eFigure 10.7: SUCRA ranking curve for gastrointestinal bleeding ... 58
eFigure 10.8 SUCRA ranking curve for myocardial infarction ... 59
S11 Appendix: Cluster rank plot ... 59
eFigure 11.1: Cluster rank incorporating risk estimates of stroke or systemic embolism (efficacy) vs. major bleeding (safety) ... 60
S12 Appendix: Sensitivity Analyses ... 61
eTable 12.1: Sensitivity analyses for the risk of stroke or systemic embolism with intervention options of different study characteristics ... 61
eTable 12.2: Sensitivity analyses for the risk of major bleeding with interventions options with different major bleeding definitions ... 62

eTable 12.3: Sensitivity analyses for the risk of major bleeding with intervention options of different study characteristics 63
eTable 12.4: Sensitivity analyses for the risk of all-cause mortality with intervention options of different study characteristics 64
S13 Appendix: Adjusted funnel plots 65
eFigure 13.1: Comparison-adjusted funnel plot for the network of stroke or systemic embolism in all comparisons 65
eFigure 13.2: Comparison-adjusted funnel plot for the network of major bleeding in all comparisons 66
eFigure 13.3: Comparison-adjusted funnel plot for the network of all-cause mortality in all comparisons 67
eFigure 13.4: Comparison-adjusted funnel plot for the network of ischemic stroke in all comparisons 68
eFigure 13.5: Comparison-adjusted funnel plot for the network of clinically relevant non-major bleeding in all comparisons 69
eFigure 13.6: Comparison-adjusted funnel plot for the network of intracranial bleeding in all comparisons 70
eFigure 13.7: Comparison-adjusted funnel plot for the network of gastrointestinal bleeding in all comparisons 71
eFigure 13.8: Comparison-adjusted funnel plot for the network of myocardial infarction in all comparisons 72
S14 Appendix: Assessment of small study effects by Egger's test in each outcome 73
S15 Appendix: Evaluation of the quality of evidence using GRADE framework for all outcomes 76
References of included studies 94

S1 Appendix:

eTable 1.1. Embase Search Strategy

#1	atrial fibrillation (121926)
#2	Anticoagulant (80605)
#3	Warfarin (80605)
#4	'self-care'/exp OR 'self-evaluation'/exp OR 'self-monitoring'/exp OR 'point of care testing'/exp (106392)
#5	Pharmacogenetic OR 'vkorc1 gene'/exp OR 'cytochrome p450 2c9'/exp OR 'vitamin K epoxide reductase'/exp (14302)
#6	'ambulatory care'/exp OR 'international normalized ratio'/exp OR 'clinics'/exp OR 'pharmacist'/exp OR 'nurse'/exp (277186)
#7	#4 OR #5 OR #6 (389724)
#8	#3 AND #7 (13765)
#9	'dabigatran'/de OR 'apixaban'/de OR 'edoxaban'/de OR 'rivaroxaban'/de (9477)
#10	'left atrial appendage closure device'/exp OR 'heart atrium appendage'/exp OR 'prostheses and orthoses'/exp (332818)
#11	#8 OR #9 OR #10 (352953)
#12	#1 AND #11 (15202)
#13	#13 AND ('clinical trials'/de OR 'controlled clinical trial'/de OR 'multicenter study'/de OR 'randomized controlled trial'/de) (1627)

eFigure 1.1. PRISMA Flow Diagram

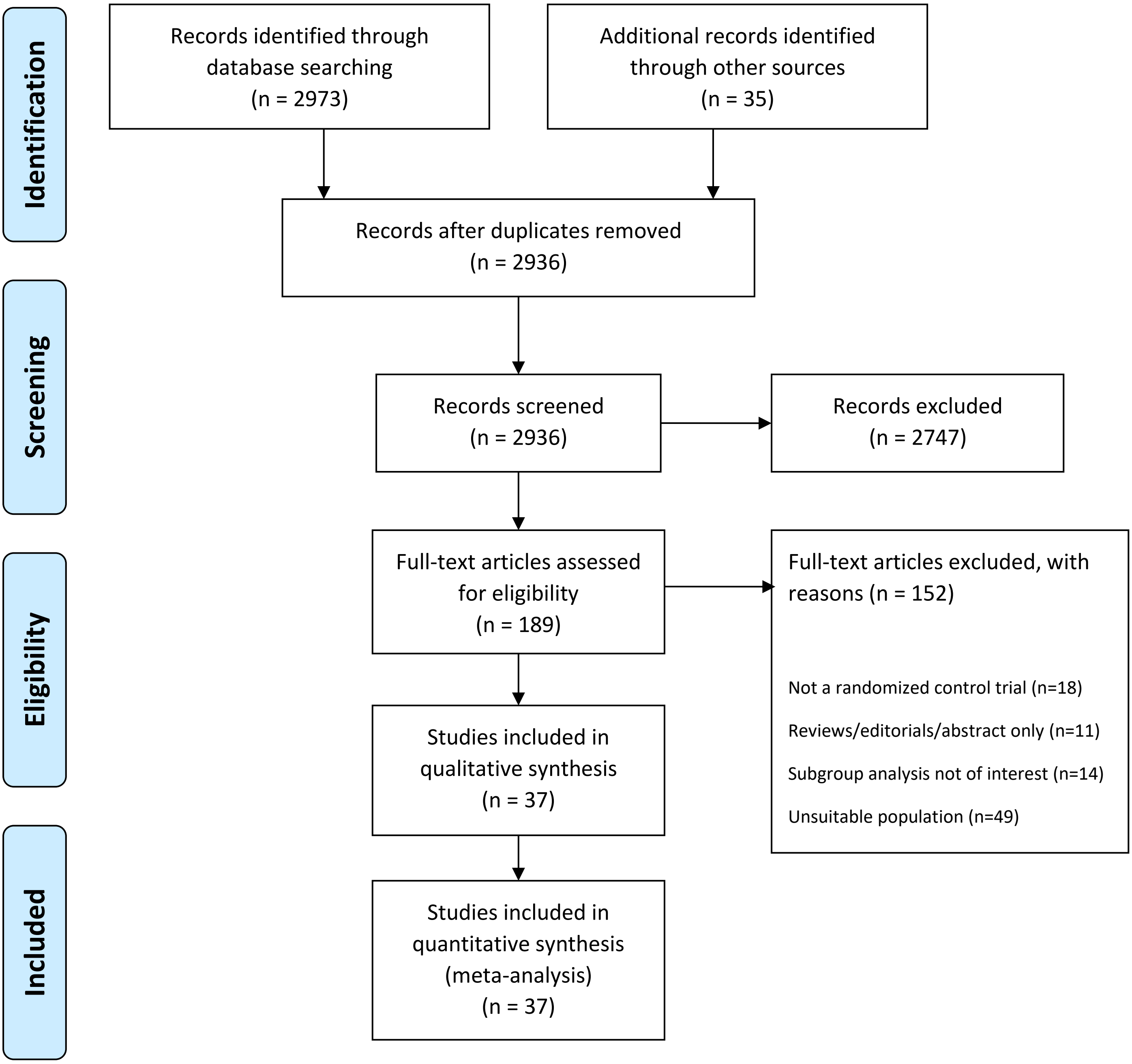

S2 Appendix: Interventions

eTable 2.1: General Characteristics of Interventions

Intervention Abbreviations	General characteristics
APX	Apixaban 5mg BD or 2.5mg BD
ASA	Aspirin of any doses
CONTROL	Placebo/Control/No treatment
CLP	Clopidogrel 75mg OD
DBG-110MG	Dabigatran 110mg BD
DBG-150MG	Dabigatran 150mg BD
EDX-30MG	Edoxaban 30mg OD
EDX-60MG	Edoxaban 60mg OD
GNT	Genotype-guided warfarin dosing
RVX	Rivaroxaban 20mg OD or 15mg OD
SM	Patient’s self-management of warfarin
ST	Patient’s self-testing of warfarin
WTH	Watchman Device insertion with temporary warfarin use

Abbreviations: APX, apixaban; ASA, aspirin; CLP, clopidogrel; DBG-110MG, dabigatran 110mg; DBG-150MG, dabigatran 150mg; EDX-30MG, edoxaban 30mg; EDX-60MG, edoxaban 60mg; GNT, genotype-guided warfarin dosing; INR, international normalized ratio; RVX, rivaroxaban; SM: self-management of warfarin; ST: self-testing of warfarin; WTH: Watchman device; BD, twice daily; OD, once daily

eFigure 2.1: Graphic Representation of Anticoagulant Interventions for Stroke Prevention in Patients with Atrial Fibrillation

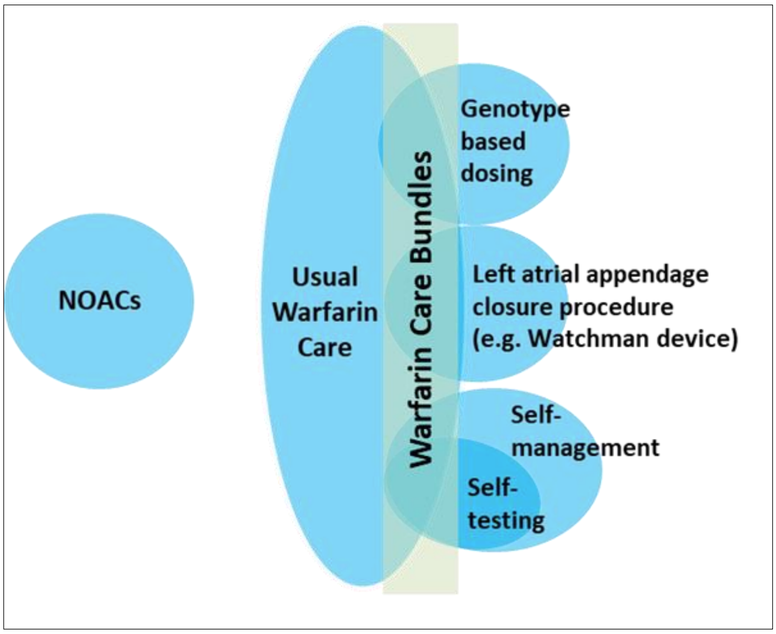

- **Usual warfarin care:** Warfarin therapy alone without any supplementary interventions but may have been delivered either in hospitals, primary care or anticoagulation clinics.

- **Warfarin Care Bundles:** A structured way of improving the process of warfarin care by performing several interventions (up to five) collectively to have aggregated beneficial effects on patient's outcomes

- **NOACs:** novel oral anticoagulants (direct thrombin inhibitors: dabigatran and factor Xa inhibitors: rivaroxaban, apixaban and edoxaban)

- Other NOACs were excluded: betrixaban because the indication for stroke prevention is still not approved; darexaban (YM50) and AZD0837 because they were discontinued, otamixaban (INN) due to its parenteral administration; letaxaban (TAK-442), LY517717 and eribaxaban (PD0348292) as no further information was available on clinical development; and ximelagatran as it was withdrawn from the market due to hepatoxicity

eTable 2.2: Detailed Description of interventions

	Anticoagulant Interventions	Descriptions
**Reference Therapy**	Usual warfarin care	Patients are routinely managed either in hospitals, primary care or in anticoagulation clinics by a range of healthcare professionals including physicians, nurses and clinical pharmacists.<br>Anticoagulation clinics were included in the usual warfarin care because in most multicenter trials, some patients in warfarin arms were monitored in anticoagulation clinics and some were monitored in primary care by a general physician. As a result, categorizing anticoagulation clinics as a standalone intervention is not feasible and was included as part of the usual warfarin care
**Warfarin care bundles***	Genotype-guided warfarin dosing	Patients receive CYP2C9 and VKORC1 genotyping. Based on their genotype status, they are then initiated on an individualized dosing regimen with the aim of achieving the correct maintenance dose more rapidly than usual warfarin care.[1]
	Patient's self-testing of warfarin	Patients are trained to use the point-of-care devices. They conduct their own international normalized ratio (INR) test using approved portable coagulometers in their own home with their test results managed by either physicians or pharmacists in the anticoagulation clinics through trans-telephonic monitoring. The anticoagulation clinic visits for this strategy would be less frequent than usual warfarin care.[2]
	Patient's self-management of warfarin	Patients are trained to use the point-of-care devices and in self-adjusting their own warfarin dosage. They conduct their own INR test using approved portable coagulometers in their own home and self-adjust their warfarin dosage according to the predefined protocol. The anticoagulation clinic visits for this strategy would be less frequent than usual warfarin care.[2]
	LAAC procedure (Watchman device) with temporary warfarin use	The Watchman device is a self-expanding device composed of a nickel-titanium frame and permeable polyester fabric covering. It is implanted via percutaneous delivery into the patient's left atrial appendage (LAA). Following the successful device implantation, patients will receive temporary warfarin and aspirin for 45 days, aspirin plus clopidogrel from 46 days to 6 months and aspirin alone thereafter.[3,4]

**NOACs**	Dabigatran, 150mg twice daily	NOACs offer potential advantages such as rapid onset and offset of action, fewer drug and food interactions, and predictable pharmacokinetics which allows the administration of fixed doses without the need for routine anticoagulation monitoring.
	Rivaroxaban, 20mg once daily	
	Apixaban, 5mg twice daily	
	Edoxaban, 60mg once daily	

*Warfarin care bundles are the combination of several interventions performed collectively to improve the quality of conventional warfarin care.

Abbreviations: LAAC, left atrial appendage closure; INR, international normalized ratio; NOACs, novel oral anticoagulants;

S3 Appendix: Characteristics of Included Studies

eTable 3.1: Descriptions of Included Studies

Study name/ First author	Year	Type of study	Location of trial (country)	Sponsor	n	Types of AF	Follow-up Period (months)	Age eligibility (years)	% AF patients	Time of outcome assessment (months)	Reporting Pattern*
Matchar DB[5]	2010	Multicentre	North America	Non-ISR	2922	NS	24 - 57	NA	82	24 - 57	1
Khan TI[6]	2004	Single centre	Europe	Non-ISR	79	NS	6	≥65	100	6	2
SMAAF[7]	2005	Single centre	Europe	ISR	202	NVAF	448 - 483	NA	100	448 - 483	3
Menendez-Jandula B[8]	2005	Single centre	Europe	ISR	737	NS	11.8 (median)	≥18	50	11.8 (median)	2
Verret L [9]	2012	Single centre	North America	ISR	117	NS	4	18-75	51	4	3
Pirmohamed M[10]	2013	Multicentre	Europe	Non-ISR	455	NS	3	≥18	72.1	3	3
ACTIVE-W[11]	2006	Multicentre	North America<br>South America<br>Europe<br>Asia<br>Oceania<br>Africa	ISR	6706	NVAF	15.4 (median)	≥55	100	15.4 (median)	1
Liu X[12]	2014	Single centre	Asia	Non-ISR	101	Persistent or Permanent NVAF	24	≥80	100	1, 3, 6, 12, 18, 24	2
SPAF II[13]	1994	Multicentre	North America	Non-ISR	1100	NVAF	37.2 (age ≤ 75)	≥60	100	27.6 (mean)	3
EAFT[14]	1993	Multicentre	Europe<br>Asia	ISR	439	Chronic or paroxysmal NVAF	27.6 (mean)	≥25	100	27.6 (mean)	1
WASPO[15]	2007	Single centre	Europe	NA	75	Permanent NVAF	12	80-90	100	12	2
Lavitola PL[16]	2010	Single centre	South America	ISR	229	NS	57 (mean)	≥18	100	57 (mean)	3
BAFTA[17]	2007	Multicentre	Europe	Non-ISR	973	NVAF or atrial flutter	32.4 (mean)	≥75	100	32.4 (mean)	1
AFASAK[18]	1989	Multicentre	Europe	ISR	1007	Chronic NVAF	24	≥18	100	24	3
AFASAK 2[19]	1998	Single centre	Europe	Non-ISR	339	Chronic NVAF	42	≥18	100	42	3
BAATAF[20]	1990	Multicentre	North America	ISR	420	Persistent or permanent NVAF	27.6 (mean)	NA	100	27.6 (mean)	3

Study name/ First author	Year	Type of study	Location of trial (country)	Sponsor	n	Types of AF	Follow-up Period (months)	Age eligibility (years)	% AF patients	Time of outcome assessment (months)	Reporting Pattern*
CAFA[21]	1991	Multicentre	North America	Non-ISR	378	Chronic or paroxysmal NVAF	15.2 (mean)	≥19	100	15.2 (mean)	1
JAST[22]	2006	Multicentre	Asia	Non-ISR	871	NVAF	25.6 (mean)	NA	100	25.6 (mean)	3
Chen KP[23]	2012	Multicentre	Asia	Non-ISR	440	NVAF	15 (mean)	50-80	100	15 (mean)	2
SPAF I[24]	1991	Multicentre	North America	Non-ISR	421	NVAF	15.6 (mean)	NA	100	15.6 (mean)	3
PREVAIL[4]	2014	Multicentre	North America	ISR	407	NVAF	11.8 (mean)	NA	100	11.8 (mean)	3
PROTECT AF[3]	2014	Multicentre	North America<br>Europe	ISR	707	NVAF	45.6 (mean)	≥18	100	45.6 (mean)	3
RELY[25]	2009	Multicentre	North America<br>South America<br>Europe<br>Asia<br>Oceania<br>Africa	ISR	18113	NVAF	24 (mean)	≥18	100	24 (mean)	2
ROCKET[26]	2011	Multicentre	North America<br>South America<br>Europe<br>Asia<br>Oceania<br>Africa	ISR	14264	NVAF	23.6 (median)	≥18	100	23.6 (median)	2
ARISTOTLE J[27]	2011	Multicentre	Asia	ISR	222	NVAF	3	≥20	100	3	2
ENGAGE TIMI-48[28]	2013	Multicentre	North America<br>South America<br>Europe<br>Asia<br>Oceania<br>Africa	ISR	21105	NVAF	34,1 (median)	≥21	100	34.1 (median)	2
ARISTOTLE[29]	2011	Multicentre	North America<br>South America<br>Europe<br>Asia<br>Oceania<br>Africa	ISR	18201	NVAF or atrial flutter	21.6 (median)	NA	100	21.6 (median)	3

Study name/ First author	Year	Type of study	Location of trial (country)	Sponsor	n	Types of AF	Follow-up Period (months)	Age eligibility (years)	% AF patients	Time of outcome assessment (months)	Reporting Pattern*
Weitz JI[30]	2010	Multicentre	North America<br>South America<br>Europe	ISR	719	Persistent NVAF	3	18-85	100	3	2
J-ROCKET[31]	2012	Multicentre	Asia	ISR	1278	NVAF	30	≥20	100	30	2
TRIPLE AXEL[32]	2017	Multicentre	Asia	ISR	183	NVAF	1	≥19	100	1	2
Chung N[33]	2011	Multicentre	Asia	ISR	234	NVAF	3	18-80	100	3	2
PETRO[34]	2007	Multicentre	North America<br>Europe	ISR	236	Paroxysmal, persistent or permanent NVAF	3	NA	100	3	2
Mao L[35]	2014	Single centre	Asia	Non-ISR	353	NVAF	NA	NA	100	NA	2
Yamashita T[36]	2012	Multicentre	Asia	ISR	391	NVAF	3	≥20	100	3	2
AVERROES[37]	2011	Multicentre	North America<br>South America<br>Europe<br>Asia<br>Africa	ISR	5599	NVAF	13.2 (mean)	≥50	100	13.2 (median)	2
Shosha RI[38]	2017	Single centre	Africa	NA	60	NVAF	3	18-60	100	3	2
Boehringer Ingelheim[16]	2014	Multicentre	Japan	ISR	156	Paroxysmal, persistent or permanent NVAF	3	≥20	100	3	2

ISR=industry-sponsored research, NA= not available, NS = not specified, NVAF = non-valvular atrial fibrillation

*1=Number of patients whose first event is of a given type, patients censored thereafter; 2=Number of patients experiencing at least one event of each given type; 3=Total number of events of each type

eTable 3.2: Descriptions of Participants of Included Studies

Study name/ First author	Treatment (n)	Age (year)		Male (%)	HTN (%)	DM (%)	HF (%)	Previous stroke or TIA (%)	DLP (%)	MI (%)	Smokers (%)	TTR (%)	Average CHADS$_2$ Score	CHADS$_2$ Score ≥2 (%)
Matchar DB[5]	UC+ST	66.6	Mean	98	71	9	28	9	NA	NA	NA	66.2	1.94	59.4
	UC	67.4	Mean	98	69	34	30	10	NA	NA	NA	62.4	1.95	61
Khan TI[6]	UC+ST	75	Median	65	NA	NA	NA	NA	NA	NA	7	71.1	NA	NA
	UC	73	Median	48.7	NA	NA	NA	NA	NA	NA	5	63.2	NA	NA
SMAAF[7]	UC+SM	64.6	Mean	71.4	NA	NA	NA	NA	NA	NA	NA	67.8	NA	NA
	UC	64.1	Mean	61.4	NA	NA	NA	NA	NA	NA	NA	58.5	NA	NA
Menendez-Jandula B[8]	UC+SM	65.5	Mean	52	48.6	15.4	NA	NA	NA	NA	NA	60.8	NA	NA
	UC	64.5	Mean	54	42.8	13.6	NA	NA	NA	NA	NA	58.4	NA	NA
Verret L [9]	UC+SM	58.4	Mean	67.2	NA	NA	NA	NA	NA	NA	NA	80	NA	NA
	UC	57	Mean	69.6	NA	NA	NA	NA	NA	NA	NA	75.5	NA	NA
Pirmohamed M[10]	UC+GNT	67.8	Mean	64.2	NA	NA	NA	NA	NA	NA	10.3	67.4	NA	NA
	UC	66.9	Mean	57.9	NA	NA	NA	NA	NA	NA	12.8	60.3	NA	NA
ACTIVE-W[11]	UC	70.4	Mean	66	82	21	31	15	NA	18	NA	63.8	2.0	NA
	ASA+CLP	70,2	Mean	67	83	21	30	15	NA	17	NA	NA	2.0	NA
Liu X[12]	UC	84.8	Median	60.8	39.2	21.6	21.6	NA	NA	15	25.5	NA	2.9*	NA
	ASA	84.4	Median	60	38	22.	20.0	NA	NA	14	24	NA	2.8*	NA
SPAF II[13]	UC	70	Mean	NA	NA	NA	NA	NA	NA	NA	NA	NA	NA	NA
	ASA	70	Mean	NA	NA	NA	NA	NA	NA	NA	NA	NA	NA	NA
EAFT[14]	UC	71	Mean	55	19.1	5.3	3.6	12	5.3	7	NA	NA	NA	NA
	CONTROL	70	Mean	58	19.1	6.5	4.7	14.9	3.3	10	NA	NA	NA	NA
WASPO[15]	UC	83.5	Median	55	49	3	NA	NA	NA	NA	NA	69.2	NA	NA
	ASA	82.6	Median	54	46	5	NA	NA	NA	NA	NA	NA	NA	NA
Lavitola PL[16]	UC	NS	NS	20.2	NA	12.6	NA	NA	11.8	NA	NA	51.3	NA	NA
	ASA	NS	NS	22.7	NA	12.7	NA	NA	9.1	NA	NA	NA	NA	NA
BAFTA[17]	UC	81.5	Mean	55	53	14	20	13	NA	10	NA	67	NA	NA
	ASA	81.5	Mean	54	55	13	19	12	NA	12	NA	NA	NA	NA
AFASAK[18]	UC	72.8	Median	53	32	7	50	6	NA	8	40	73	NA	NA
	ASA	75.1	Median	55	33	8	54	5.1	NA	7	37	NA	NA	NA
	CONTROL	74.6	Median	54	31	10	51	6.3	NA	8	35	NA	NA	NA
AFASAK 2[19]	UC	73.2	Mean	57	47	14	70	8	NA	8	31	73	NA	NA
	ASA	73.1	Mean	65	43	10	70	8	NA	7	38	NA	NA	NA
BAATAF[20]	UC	68.5	Mean	75	51	14	24	3	NA	10	7	NA	NA	NA
	CONTROL	67.5	Mean	70	51	16	28	3	NA	16	10	NA	NA	NA
CAFA[21]	UC	68	Mean	75.9	43.3	13.9	23.5	3.2	NA	15	NA	43.7	NA	NA

Study name/ First author	Treatment (n)	Age (year)		Male (%)	HTN (%)	DM (%)	HF (%)	Previous stroke or TIA (%)	DLP (%)	MI (%)	Smokers (%)	TTR (%)	Average $CHADS_2$ Score	$CHADS_2$ Score ≥2 (%)
	CONTROL	67.4	Mean	73.3	34	10	20.4	4.2	NA	12	NA	NA	NA	NA
JAST[22]	ASA	65.5	Mean	36.6	36.6	12.7	8.3	2.6	23.9	NA	32.8	NA	NA	NA
	CONTROL	64.8	Mean	40.4	40.4	15.3	10.1	2.5	21.2	NA	27.9	NA	NA	NA
Chen KP[23]	UC	66.8	Mean	59	59	12.1	61.5	20.9	15.9	5.4	NA	51.2	NA	NA
	ASA	67.6	Mean	66.2	66.2	14.9	65.6	15.4	17.4	3	NA	NA	NA	NA
SPAF I[24]	UC	65	Mean	74	49	12	14	8.0	NA	10	13	NA	NA	NA
	CONTROL	66	Mean	70	55	19	19	8.0	NA	6	13	NA	NA	NA
PREVAIL[4]	UC+WTH	74	Mean	67.7	88.5	33.8	23.4	27.5	NA	NA	NA	NA	2.6	92.2
	UC	74.9	Mean	74.6	97.1	29.7	23.2	28.3	NA	NA	NA	68	2.6	91.3
PROTECT AF[3]	UC+WTH	71.7	Mean	70.4	89.6	24.4	26.8	17.7	NA	NA	NA	NA	2.2	66.3
	UC	72.7	Mean	70.1	90.2	29.5	27	20.1	NA	NA	NA	70	2.3	73
RELY[25]	DBG-150MG	71.5	Mean	63.2	78.9	23.1	31.8	20.3	NA	16.9	NA	NA	2.2	67.8
	DBG-110MG	71.4	Mean	64.3	78.8	23.4	32.2	19.9	NA	16.8	NA	NA	2.1	67.4
	UC	71.6	Mean	63.3	78.9	23.4	31.9	19.8	NA	16.1	NA	64	2.1	69.1
ROCKET[26]	RVX	73	Median	60.3	90.3	40.4	62.6	54.9	NA	16.6	NA	NA	3.48	100
	UC	73	Median	60.3	90.8	39.5	62.3	54.6	NA	18	NA	55	3.46	100
ARISTOTLE J[27]	APX	69.7	Mean	83.8	82.4	25	0.7	28.4	NA	NA	NA	NA	1.9	60.1
	UC	71.7	Mean	81.1	85.1	20.3	2.7	27	NA	NA	NA	NA	1.9	50
ENGAGE TIMI-48[28]	EDX-60MG	72	Median	62.1	93.7	36.4	58.2	28.1	NA	NA	NA	NA	2.8	NA
	EDX-30MG	72	Median	61.2	93.5	36.2	56.6	28.5	NA	NA	NA	NA	2.8	NA
	UC	72	Median	62.5	93.6	35.8	57.5	28.3	NA	NA	NA	64.9	2.8	NA
ARISTOTLE[29]	APX	70	Median	64.5	87.3	25.0	35.5	19.2	NA	14.5	NA	NA	2.1	66
	UC	70	Median	65	87.6	24.9	35.4	19.7	NA	13.9	NA	62.2	2.1	66
Weitz JI[30]	EDX-60MG	64.9	Mean	66.2	NA	NA	NA	NA	NA	NA	NA	NA	NA	100
	EDX-30MG	65.2	Mean	59.6	NA	NA	NA	NA	NA	NA	NA	NA	NA	100
	UC	66	Mean	60.4	NA	NA	NA	NA	NA	NA	NA	49.7	NA	100
J-ROCKET[31]	RVX	71	Mean	82.9	79.5	39	41.3	63.8	NA	7	NA	NA	3.27	100
	UC	71.2	Mean	78.2	79.5	37.1	40.2	63.4	NA	8.3	NA	65	3.22	100
TRIPLE AXEL[32]	RVX	70.2	Mean	57.9	68.4	25.3	NA	100	16.8	NA	NA	NA	2.7	71.6
	UC	70.6	Mean	59.1	59.1	11.4	NA	100	20.5	NA	NA	46.6	2.3	71.6
Chung N[33]	EDX-60MG	65.9	Mean	68.8	73.8	27.5	31.3	23.8	NA	NA	NA	NA	1.9	53.8
	EDX-30MG	64.9	Mean	64.6	70.9	38	22.8	26.6	NA	NA	NA	NA	2.0	58.2
	UC	64.5	Mean	62.7	69.3	22.7	32.0	22.7	NA	NA	NA	45.1	1.8	46.7
PETRO[34]	DBG-150MG	70	Mean	81.3	71	27	31.3	17.5	NA	NA	72.3	NA	NA	NA

Study name/ First author	Treatment (n)	Age (year)		Male (%)	HTN (%)	DM (%)	HF (%)	Previous stroke or TIA (%)	DLP (%)	MI (%)	Smokers (%)	TTR (%)	Average $CHADS_2$ Score	$CHADS_2$ Score ≥2 (%)
	UC	69	Mean	84.3	70	21.4	34.3	18.6	NA	NA	75.7	57.2	NA	NA
Mao L[35]	RVX	75	Median	61	90.4	41.8	60.5	49.7	NA	16.9	NA	NA	3.39	100
	UC	75	Median	62.5	91.5	39.8	61.4	48.9	NA	17.6	NA	NA	3.41	100
Yamashita T[36]	EDX-60MG	68.4	Mean	81	74	21	24	30	NA	NA	18	NA	2.1	NA
	EDX-30MG	68.8	Mean	81.7	75	18	24	23	NA	NA	18	NA	1.9	NA
	UC	70	Mean	82.9	71	31	33	30	NA	NA	16	75.7	2.2	NA
AVERROES[37]	APX	70	Mean	59	86	19	40	14	NA	NA	NA	NA	2.0	64.2
	ASA	54	Mean	58	87	20	38	13	NA	NA	NA	NA	2.1	63.3
Shosha RI[38]	RVX	55	Mean	60	53.3	13.3	36.6	26.6	NA	NA	NA	NA	NA	40
	UC	68.3	Mean	30	40	26.6	30	10	NA	NA	NA	55	NA	33.3
Boehringer Ingelheim[16]	DBG-150MG	68.3	Mean	91.4	NA	NA	NA	NA	NA	NA	NA	NA	NA	NA
	DBG-110MG	69.9	Mean	78.3	NA	NA	NA	NA	NA	NA	NA	NA	NA	NA
	UC	67.4	Mean	91.9	NA	NA	NA	NA	NA	NA	NA	NA	NA	NA

APX= apixaban; ASA = aspirin; ASA+CLP = aspirin + clopidogrel; DBG-110mg=dabigatran 110mg; DBG-150MG=dabigatran 150mg; EDX-30MG=edoxaban 30mg; EDX-60MG=edoxaban 60mg; RVX=rivaroxaban; UC=usual care warfarin; GNT = genotype-guided warfarin dosing; SM=patient's self-management of warfarin; ST=patient's self-testing of warfarin; WTH=watchman device; HTN=hypertension; DM=diabetes melitus; HF=heart failure; TIA=transient ischemic attack; MI=myocardial infarction; TTR=time in therapeutic range; $CHADS_2$ = congestive heart failure, hypertension, age ≥75 years, diabetes and previous stroke; NA=not available

eTable 3.5: Details of Reported Concurrent Medications Received in the Included Studies

Study name/ First author	Treatment (n)	ACEi/ARBs (%)	Beta-blockers (%)	Amiodarone (%)	Statins (%)	Digoxin or digitalis preparations (%)	Verapamil or diltiazem (%)
Matchar DB[5]	UC+ST	NA	NA	8	NA	NA	NA
	UC	NA	NA	8	NA	NA	NA
Khan TI[6]	UC+ST	NA	NA	NA	NA	NA	NA
	UC	NA	NA	NA	NA	NA	NA
SMAAF[7]	UC+SM	NA	NA	NA	NA	NA	NA
	UC	NA	NA	NA	NA	NA	NA
Menendez-Jandula B[8]	UC+SM	NA	NA	NA	NA	NA	NA
	UC	NA	NA	NA	NA	NA	NA
Verret L [9]	UC+SM	NA	NA	NA	NA	NA	NA
	UC	NA	NA	NA	NA	NA	NA
Pirmohamed M[10]	UC+GNT	NA	NA	NA	NA	NA	NA
	UC	NA	NA	NA	NA	NA	NA
ACTIVE-W[11]	UC	15	56	NA	37	37	NA
	ASA+CLP	15	58	NA	38	37	NA
Liu X[12]	UC	78.4	72.5	15.7	58.9	13.8	NA
	ASA	84	70	14	62	16	NA
SPAF II[13]	UC	NA	NA	NA	NA	NA	NA
	ASA	NA	NA	NA	NA	NA	NA
EAFT[14]	UC	NA	NA	NA	NA	NA	NA
	CONTROL	NA	NA	NA	NA	NA	NA
WASPO[15]	UC	NA	NA	NA	NA	NA	NA
	ASA	NA	NA	NA	NA	NA	NA
Lavitola PL[16]	UC	NA	NA	NA	NA	NA	NA
	ASA	NA	NA	NA	NA	NA	NA
BAFTA[17]	UC	NA	NA	NA	NA	NA	NA
	ASA	NA	NA	NA	NA	NA	NA
AFASAK[18]	UC	NA	NA	NA	NA	NA	NA
	ASA	NA	NA	NA	NA	NA	NA
	CONTROL	NA	NA	NA	NA	NA	NA
AFASAK 2[19]	UC	NA	NA	NA	NA	NA	NA
	ASA	NA	NA	NA	NA	NA	NA
BAATAF[20]	UC	NA	NA	NA	NA	NA	NA
	CONTROL	NA	NA	NA	NA	NA	NA
CAFA[21]	UC	NA	NA	NA	NA	NA	NA
	CONTROL	NA	NA	NA	NA	NA	NA
JAST[22]	ASA	NA	NA	NA	NA	NA	NA
	CONTROL	NA	NA	NA	NA	NA	NA
Chen KP[23]	UC	NA	NA	NA	NA	NA	NA
	ASA	NA	NA	NA4	NA	NA	NA
SPAF I[24]	UC	NA	NA	NA	NA	NA	NA
	CONTROL	NA	NA	NA	NA	NA	NA
PREVAIL[4]	UC+WTH	NA	NA	NA	NA	NA	NA
	UC	NA	NA	NA	NA	NA	NA
PROTECT AF[3]	UC+WTH	NA	NA	NA	NA	NA	NA
	UC	NA	NA	NA	NA	NA	NA
RELY[25]	DBG-150MG	66.7	63.7	10.9	43.9	NA	NA
	DBG-110MG	66.3	62.9	10.4	44.9	NA	NA
	UC	65.5	61.8	10.7	44.4	NA	NA
ROCKET[26]	RVX	NA	65.1	NA	42.96	38.78	NA
	UC	NA	65.7	NA	43.19	38.85	NA
ARISTOTLE J[27]	APX	NA	NA	NA	NA	NA	NA
	UC	NA	NA	NA	NA	NA	NA
ENGAGE TIMI-48[28]	EDX-60MG	NA	NA	12.3	NA	29.5	NA
	EDX-30MG	NA	NA	11.4	NA	29.5	NA
	UC	NA	NA	11.8	NA	30.9	NA
ARISTOTLE[29]	APX	70.9	63.6	11.1	45	32	30.1
	UC	70.1	62.6	11.5	45.1	32.1	31.1

Study name/ First author	Treatment (n)	ACEi/ARBs (%)	Beta-blockers (%)	Amiodarone (%)	Statins (%)	Digoxin or digitalis preparations (%)	Verapamil or diltiazem (%)
Weitz JI[30]	EDX-60MG	NA	NA	NA	NA	NA	NA
	EDX-30MG	NA	NA	NA	NA	NA	NA
	UC	NA	NA	NA	NA	NA	NA
J-ROCKET[31]	RVX	NA	NA	NA	NA	NA	NA
	UC	NA	NA	NA	NA	NA	NA
TRIPLE AXEL[32]	RVX	NA	NA	NA	NA	NA	NA
	UC	NA	NA	NA	NA	NA	NA
Chung N[33]	EDX-60MG	NA	NA	NA	NA	NA	NA
	EDX-30MG	NA	NA	NA	NA	NA	NA
	UC	NA	NA	NA	NA	NA	NA
PETRO[34]	DBG-150MG	69.8	73	5.4	60	45	18.7
	UC	81.4	70	8.5	53	45.7	20
Mao L[35]	RVX	NA	NA	NA	NA	NA	NA
	UC	NA	NA	NA	NA	NA	NA
Yamashita T[36]	EDX-60MG	NA	NA	NA	NA	NA	NA
	EDX-30MG	NA	NA	NA	NA	NA	NA
	UC	NA	NA	NA	NA	NA	NA
AVERROES[37]	APX	64	56	11	31	29	9
	ASA	64	55	12	31	27	9
Shosha RI[38]	RVX	NA	NA	NA	NA	NA	NA
	UC	NA	NA	NA	NA	NA	NA
Boehringer Ingelheim[16]	DBG-150MG	NA	NA	NA	NA	NA	NA
	DBG-110MG	NA	NA	NA	NA	NA	NA
	UC	NA	NA	NA	NA	NA	NA

APX= apixaban; ASA = aspirin; ASA+CLP = aspirin + clopidogrel; DBG-110mg=dabigatran 110mg; DBG-150MG=dabigatran 150mg; EDX-30MG=edoxaban 30mg; EDX-60MG=edoxaban 60mg; RVX=rivaroxaban; UC=usual care warfarin; GNT = genotype-guided warfarin dosing; SM=patient's self-management of warfarin; ST=patient's self-testing of warfarin; WTH=watchman device; ACEi/ARBs=angiotensin-converting enzyme inhibitors/angiotensin receptor blockers

S4 Appendix: Matching of Major Bleeding Definitions

In order to minimize the heterogeneity on the definitions of major bleeding across the included studies, major bleeding events were matched against the standardized bleeding end-point definitions adopted by the Bleeding Academic Research Consortium (BARC).[39] Major bleeding outcome was collected based on BARC3-5 criteria because this bleeding definition was standardized to include both clinical and laboratory information. Furthermore, BARC definition was the most updated bleeding definition.

We directly collected major bleeding outcome from any studies reported outcome as BARC definition. If not, the compatibility criteria were considered. The compatible definitions must be standardized based on BARC 3-5 criteria and they must not contain any lower severity of bleeding. For instance, ISTH major bleeding definition (fatal bleeding, symptomatic bleeding in a critical area or organ such as intracranial, reduction of haemoglobin ≥2g/dL or transfusion of two or more units of whole blood) could be categorized into BARC type 3A (reduction of haemoglobin 3 to <5g/Dl or transfusion with overt bleeding), type 3B (reduction of haemoglobin ≥5g/dL), type 3c (intracranial haemorrhage), and type 5 (fatal bleeding). Therefore, ISTH major bleeding was a compatible definition and ISTH major bleeding outcomes could be collected into our analysis. In contrast, a non-official definition such as 'intracranial bleeding and/or requiring blood transfusion or surgical intervention and/or hospitalization' could not be categorized as compatible definition due to the inclusion of hospitalization criteria which was unclear in severity and might not be compatible with BARC 3-5.

eTable 4.1: Major Bleeding Definitions of Each Study and Compatibility with BARC 3-5, TIMI Major, GUSTO severe and ISTH Major Bleeding Definitions

Study name/First Author	Definition of major bleeding	Details in case of non-official bleeding definition	Compatible with BARC 3-5	Compatible with TIMI major	Compatible with GUSTO severe	Compatible with ISTH major
ACTIVE-W[11]	Non-official definition	Any bleeding requiring transfusion of at least two units of red blood cells or equivalent of whole blood, or which was severe	BARC 3A, 3B	Incompatible due to transfusion criteria	Incompatible due to transfusion criteria	Reported as ISTH
AFASAK[18]	Non-official definition	Bleeding requiring medical intervention	Incompatible due to medical intervention criteria	Incompatible due to medical intervention criteria	Incompatible due to medical intervention criteria	Incompatible due to medical intervention criteria
AFASAK 2[19]	Non-official definition	Fatal, life-threatening or potentially life-threatening, requiring surgical treatment or blood transfusion	Incompatible due to surgical treatment criteria	Incompatible due transfusion and surgical treatment criteria	Incompatible due transfusion and surgical treatment criteria	Incompatible due to surgical treatment criteria
ARISTOTLE[29]	ISTH		BARC 3A, 3B, 3C, 5	Incompatible due to hemoglobin and transfusion criteria	Incompatible due to hemoglobin and transfusion criteria	Reported as ISTH
ARISTOTLE J[27]	ISTH		BARC 3A, 3B, 3C, 5	Incompatible due to hemoglobin and transfusion criteria	Incompatible due to hemoglobin and transfusion criteria	Reported as ISTH
AVERROES[37]	Non-official definition	Clinically overt bleeding accompanied by one or more of the following: a decrease in the hemoglobin level of 2 g/dL or more over a 24-hour period, transfusion of 2 or more units of packed red cells, bleeding at a critical site (intracranial, intraspinal, intraocular, pericardial, intraarticular, intramuscular with compartment syndrome, or retroperitoneal), or fatal bleeding	BARC 3A, 3B, 3C, 5	Incompatible due to hemoglobin and transfusion criteria	Incompatible due to hemoglobin and transfusion criteria	Compatible
BAATAF[20]	Non-official definition	Intracranial bleeding, fatal bleeding, or bleeding leading to the transfusions of 4 units of blood within 48 hours	BARC 3A, 3C, 5	Incompatible due to transfusion criteria	Incompatible due to transfusion criteria	Incompatible due to transfusion criteria
BAFTA[17]	Non-official definition	Intracranial hemorrhage or fatal hemorrhage, or one that resulted in the need for transfusion or surgery	Incompatible due to surgery criteria	Incompatible due to transfusion criteria	Incompatible due transfusion criteria	Incompatible due surgery criteria
Boehringer Ingelheim[16]	Non-official definition	Any bleed fulfilling one of the following conditions: Fatal or life-threatening, retroperitoneal, intracranial, intraocular, or intraspinal bleeding (verified by objective testing, bleeding requiring surgical treatment, clinically overt bleeding leading to a transfusion of 4.5 units (equal to 2 units in EU/US) or more, clinically overt bleeding leading to a fall in hemoglobin of at least 2 g/dL	Incompatible due to surgical treatment criteria	Incompatible due to hemoglobin, transfusion and surgical treatment criteria	Incompatible due to hemoglobin and transfusion criteria	Incompatible due to surgical treatment criteria
CAFA[21]	Non-official definition	Any bleeding episode associated with a 2g/dL decrease in serum hemoglobin or requiring a blood transfusion or bleeding into a sensitive location such as the pericardium or retina	BARC 3A, 3B, 3C	Incompatible due to hemoglobin and transfusion criteria	Incompatible due to hemoglobin and transfusion criteria	Compatible
Chen KP[23]	Non-official definition	Intracranial bleeding, fatal bleeding, or bleeding leading to the transfusion of four or more units of blood	BARC 3A, 3C, 5	Incompatible due to transfusion criteria	Incompatible due to transfusion criteria	Incompatible due to transfusion criteria
Chung N[33]	Non-official definition	Fatal, bleeding associated with ≥2 g/dl drop in hemoglobin, transfusion ≥800 ml of packed red blood cells or whole blood, and bleeding into a critical area or organ (retroperitoneal, intracranial,	BARC 3A, 3B, 3C	Incompatible due to hemoglobin and transfusion criteria	Incompatible due to hemoglobin and transfusion criteria	Compatible

Study name/First Author	Definition of major bleeding	Details in case of non-official bleeding definition	Compatible with BARC 3-5	Compatible with TIMI major	Compatible with GUSTO severe	Compatible with ISTH major
		intraocular, intraspinal, intra-articular or pericardial or intramuscular with compartment syndrome)				
EAFT[14]	Non-official definition	Bleeding that required hospital admission, blood transfusion or surgery, or when they caused a permanent increase in disability	Incompatible due to hospitalization and permanent disability criteria	Incompatible due to hospitalization and permanent disability criteria	Incompatible due to hospitalization and permanent disability criteria	Incompatible due to hospitalization and permanent disability criteria
ENGAGE TIMI 48[28]	ISTH		BARC 3A, 3B, 3C, 5	Incompatible due to hemoglobin and transfusion criteria	Incompatible due to hemoglobin and transfusion criteria	Reported as ISTH
JAST[22]	Non-official definition	Fatal bleeding, bleeding needed for hospital admission for treatment, blood transfusion, or a decrease of hemoglobin concentration 4 g/dL	Incompatible due hospitalization criteria	Incompatible due to hemoglobin, transfusion and hospitalization criteria	Incompatible due to hemoglobin, transfusion and hospitalization criteria	Incompatible due to hospitalization criteria
J-ROCKET[31]	Non-official definition	Clinically overt bleeding that was associated with a fall in hemoglobin ≥20g/L, transfusion of ≥2 units of packed red blood cells or whole blood, or involved a critical site (intracranial, intraspinal, intraocular, pericardial, intra-articular, intramuscular with compartment syndrome, retroperitoneal hemorrhage), or had a fatal outcome	BARC 3A, 3B, 3C, 5	Incompatible due to hemoglobin and transfusion criteria	Incompatible due to hemoglobin and transfusion criteria	Compatible
Khan TI[6]	Non-official definition	Bleeding that was fatal, or was life-threatening, or was potentially life-threatening, or led to severe blood loss, or led to surgical treatment, or led to moderate blood loss that was acute or subacute, and was not explained by trauma or surgery or bleeding that led directly to hospitalization	Incompatible due to surgical treatment and hospitalization criteria	Incompatible due to surgical treatment and hospitalization criteria	Incompatible due to surgical treatment and hospitalization criteria	Incompatible due to surgical treatment and hospitalization criteria
Lavitola PL[16]	X		X	X	X	X
Liu X[12]	Non-official definition	A Hb decrease of at least 2g/dL, a need for at least 2-unit whole blood transfusion, or symptomatic hemorrhage in major locations or organs	BARC 3A, 3B, 3C	Incompatible due to hemoglobin and transfusion criteria	Incompatible due to hemoglobin and transfusion criteria	Compatible
Mao L[35]	Non-official definition	Fatal bleeding, decreased in hemoglobin ≥ 2g/dL, bleeding requiring transfusion or bleeding into a critical area or organ (intracranial, intraocular)	BARC 3A, 3B, 3C	Incompatible due to hemoglobin and transfusion criteria	Incompatible due to hemoglobin and transfusion criteria	Compatible
Matchar DB[5]	Non-official definition	Severe: Retroperitoneal, intracranial, intraspinal, intra-ocular, or pericardial bleeding or any other source of bleeding that results in hemodynamic compromise<br><br>Moderate: Bleeding requiring transfusion of red blood cells or whole blood but does not result in hemodynamic compromise or bleeding associated with a drop in hemoglobin of ≥ 2 g/dL (rather than a drop ≥ 5g/dL) from baseline<br><br>Surgical Bleeding: Patient had surgical procedure, was transfused with blood, and any of the following occurred: (i) during intra	Incompatible due to surgical bleeding criteria	Incompatible due to hemoglobin, transfusion, and surgical bleeding criteria	Incompatible due to hemoglobin, transfusion, and surgical bleeding criteria	Incompatible due to surgical bleeding criteria

Study name/First Author	Definition of major bleeding	Details in case of non-official bleeding definition	Compatible with BARC 3-5	Compatible with TIMI major	Compatible with GUSTO severe	Compatible with ISTH major
		operative period, number of units transfused was more than number specified in OR schedule sent to blood bank prior to surgery, (ii) during post-operative period, number of units transfused was higher than expected by operating physician and met other criteria for major bleed				
Menéndez-Jándula B[8]	Non-official definition	Life-threatening bleeding or bleeding requiring transfusion or hospital admission	Incompatible due to hospitalization criteria	Incompatible due to transfusion and hospitalization criteria	Incompatible due to transfusion and hospitalization criteria	Incompatible due to hospitalization criteria
PETRO[34]	Non-official definition	Fatal or life-threatening retroperitoneal, intracranial, intraocular, or intraspinal bleeding; or bleeding requiring surgery or transfusion of 2 U or associated with a decrease in hemoglobin of 2.0 g/Dl	BARC 3A, 3B, 3C	Incompatible due to hemoglobin and transfusion criteria	Incompatible due to hemoglobin and transfusion criteria	Compatible
Pirmohamed M[10]	ISTH		BARC 3A, 3B, 3C, 5	Incompatible due to hemoglobin and transfusion criteria	Incompatible due to hemoglobin and transfusion criteria	Reported as ISTH
PREVAIL[4]	X		X	X	X	X
PROTECT AF[3]	Non-official definition	Intracranial or bleeding requiring transfusion	BARC 3A, 3C	Incompatible due to transfusion criteria	Incompatible due to transfusion criteria	Compatible
RELY[25]	Non-official definition	A reduction in the hemoglobin level of at least 20 g/L, transfusion of at least 2 units of blood, or symptomatic bleeding in a critical area or organ	BARC 3A, 3B, 3C	Incompatible due to hemoglobin and transfusion criteria	Incompatible due to hemoglobin and transfusion criteria	Compatible
ROCKET[26]	Non-official definition	Clinically overt bleeding associated with any of the following: fatal outcome, involvement of a critical anatomic site (intracranial, spinal, ocular, pericardial, articular, retroperitoneal, or intramuscular with compartment syndrome), fall in hemoglobin concentration >2 g/dL, transfusion of >2 units of whole blood or packed red blood cells, or permanent disability	BARC 3A, 3B, 3C, 5	Incompatible due to hemoglobin and transfusion criteria	Incompatible due to hemoglobin and transfusion criteria	Compatible
Shosha RI[38]	Non-official definition	Clinically overt and associated with any of the following: fatal outcome; involvement of a critical anatomic site (intracranial, spinal, ocular, pericardial, articular, and retroperitoneal, intraparenchymal, intraventricular, and subdural subarachnoid); fall in hemoglobin concentration>2 g/dl; transfusion of>2 U of whole blood; or packed red blood cells.	BARC 3A, 3B, 3C, 5	Incompatible due to hemoglobin and transfusion criteria	Incompatible due to hemoglobin and transfusion criteria	Compatible
SMAAF[7]	Non-official definition	Intracerebral or fatal hemorrhages which required transfusions or inpatient admission for more than 24 hours with or without surgical operation.	Incompatible due to hospitalization criteria	Incompatible due to transfusion and hospitalization criteria	Incompatible due to transfusion and hospitalization criteria	Incompatible due to hospitalization criteria
SPAF I[24]	Non-official definition	Any bleeding that Involved the central nervous system, management requiring hospitalization with transfusion and/or surgery, or permanent residual impairment.	Incompatible due to hospitalization and permanent disability criteria	Incompatible due to transfusion, hospitalization and permanent disability criteria	Incompatible due to transfusion, hospitalization and permanent disability criteria	Incompatible due to hospitalization and permanent disability criteria

Study name/First Author	Definition of major bleeding	Details in case of non-official bleeding definition	Compatible with BARC 3-5	Compatible with TIMI major	Compatible with GUSTO severe	Compatible with ISTH major
SPAF II[13]	Non-official definition	Overt bleeding that was (1) fatal, (2) life-threatening, (3) potentially life threatening, or (4) acute or subacute and led to reoperation or moderate or severe blood loss	BARC 5	Compatible	Compatible	Compatible
Triple AXEL[32]	ISTH		BARC 3A, 3B, 3C, 5	Incompatible due to hemoglobin and transfusion criteria	Incompatible due to hemoglobin and transfusion criteria	Reported as ISTH
Verret L[9]	Non-official definition	Bleeding that required treatment or medical evaluation or life-threatening or fatal	BARC 3A, 3B, 5	Incompatible due to treatment or medical evaluation criteria	Incompatible due to fatal criteria	Compatible
WASPO[15]	Non-official definition	Intracranial hemorrhage, fall in hemoglobin by >2 g/dl, need for blood transfusion	BARC 3A, 3B, 3C	Incompatible due to hemoglobin and transfusion criteria	Incompatible due to hemoglobin and transfusion criteria	Compatible
Weitz JI[30]	ISTH		BARC 3A, 3B, 3C, 5	Incompatible due to hemoglobin and transfusion criteria	Incompatible due to hemoglobin and transfusion criteria	Reported as ISTH
Yamashita T[36]	Non-official definition	Life-threatening bleeding; intracranial, intraspinal, intraocular (excluding subconjunctival), retroperitoneal, intraarticular, or intrapericardial bleeding; clinically overt bleeding accompanied by a decrease in hemoglobin of ≥20 g/L; or bleeding requiring transfusion of ≥4 units of blood (1 unit= approximately 200 ml)	BARC 3A, 3B, 3C	Incompatible due to hemoglobin and transfusion criteria	Incompatible due to hemoglobin and transfusion criteria	Compatible

BARC=Bleeding Academic Research Consortium; TIMI=Thrombolysis in Myocardial Infarction; GUSTO=Global Use of Strategies to Open Occluded Arteries; ISTH=International Society of Thrombosis and Haemostasis

S5 Appendix: Risk of Bias Assessment

eFigure 5.1: Risk of Bias Graph: review authors' judgements about each risk of bias item presented as percentages for studies including in network analyses (37 studies)

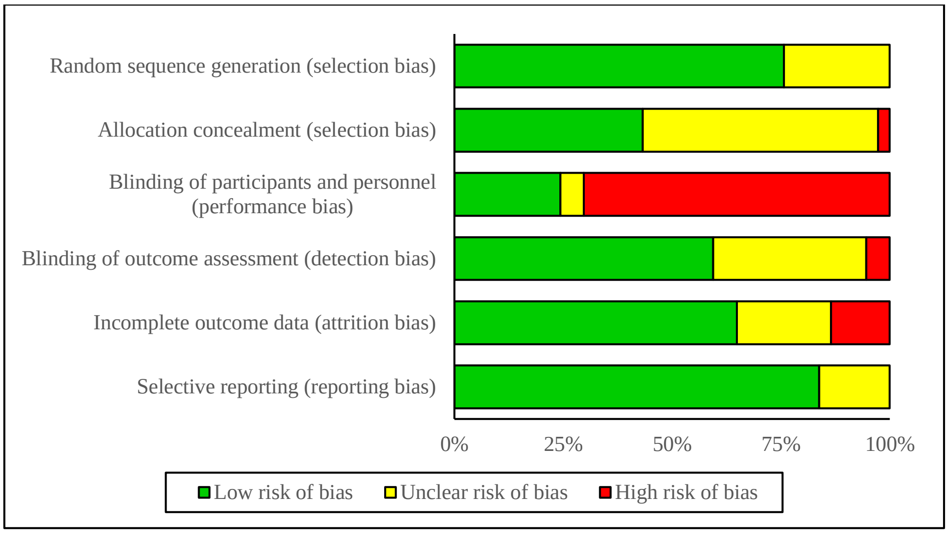

eTable 5.1: List of interventions examined by included studies for stroke prevention in AF patients

Number	Interventions
1	Apixaban
2	Aspirin
3	Aspirin + Clopidogrel
4	Control/Placebo
5	Dabigatran 110mg BD
6	Dabigatran 150mg BD
7	Edoxaban 30mg OD
8	Edoxaban 60mg OD
9	Rivaroxaban OD
10	Usual Warfarin Care
11	Genotype-guided Warfarin Dosing
12	Patient's Self-management of Warfarin
13	Patient's Self-testing of Warfarin
14	Watchman Device Insertion with Temporary Warfarin Use

eFigure 5.2: Risk of Bias Summary: review authors' judgements about each risk of bias item for each included study

Study name/ First Author	Year	Interventions Compared	1. Random sequence generation	2. Allocation concealment	3. Blinding of participants and personnel	4. Blinding of outcome assessment	5. Incomplete outcome data	6. Selective reporting
ACTIVE-W[11]	2006	3, 10	+	+	-	+	+	+
AFASAK[18]	1989	2, 4, 10	+	?	-	?	-	+
AFASAK 2[19]	1998	2, 10	+	?	-	+	?	+
ARISTOTLE[29]	2011	1, 10	+	+	+	+	+	+
ARISTOTLE J[27]	2011	1, 10	+	?	-	+	+	+
AVERROES[37]	2011	1, 2	+	+	+	+	+	+
BAATAF[20]	1990	4, 10	+	?	-	+	?	+
BAFTA[17]	2007	2, 10	+	+	-	+	+	+
Boehringer Ingelheim[16]	2014	5, 6, 10	?	?	-	?	+	+
CAFA[21]	1991	4, 10	?	-	+	?	+	+
Chen KP[23]	2012	2, 10	+	?	?	?	-	+
Chung N[33]	2011	7, 8, 10	+	+	-	+	+	+
EAFT[14]	1993	4, 10	+	+	-	+	-	+
ENGAGE TIMI 48[28]	2013	7, 8, 10	+	+	+	+	+	+
JAST[22]	2006	2, 4	?	?	-	+	+	+
J-ROCKET[31]	2012	9, 10	?	?	+	+	+	+
Khan TI[6]	2004	10, 13	+	?	-	?	+	?
Lavitola PL[16]	2010	4, 10	?	?	-	-	-	+
Liu X[12]	2014	4, 10	?	?	?	?	?	+
Mao L[35]	2014	9, 10	?	?	+	+	?	+
Matchar DB[5]	2010	10, 13	+	+	-	+	+	+
Menéndez-Jándula B[8]	2005	10, 12	+	+	-	+	+	?
PETRO[34]	2007	6, 10	?	?	-	?	+	+
Pirmohamed M[10]	2013	10, 11	+	+	-	?	+	?
PREVAIL[4]	2014	10, 14	+	+	+	?	?	+
PROTECT AF[3]	2014	10, 14	+	?	+	+	-	+
RELY[25]	2009	5, 6, 10	+	+	-	+	+	+
ROCKET[26]	2011	9, 10	+	+	+	+	+	+
Shosha RI[38]	2017	9, 10	?	?	-	?	?	+
SMAAF[7]	2005	10, 12	+	?	-	?	?	?
SPAF I[24]	1991	4, 10	+	?	-	+	+	+
SPAF II[13]	1994	4, 10	+	?	-	+	?	?

TRIPLE AXEL[32]	2017	9, 10	+	+	-	+	+	+
Verret L[9]	2012	10, 12	+	?	-	?	+	?
WASPO[15]	2007	4, 10	+	+	-	-	+	+
Weitz JI[30]	2010	7, 8, 10	+	+	-	?	+	+
Yamashita T[36]	2012	7, 8, 10	+	?	-	+	+	+

\+ Low risk of bias ? Unclear risk of bias - High risk of bias

S6 Appendix: Network Treatment of Comparison

eFigure 6.1: Network Map of Treatment Comparisons for Secondary Outcomes

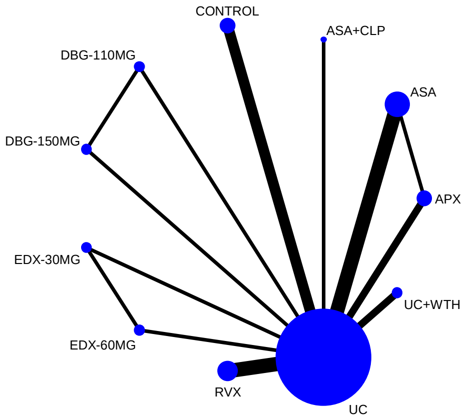

Ischemic Stroke

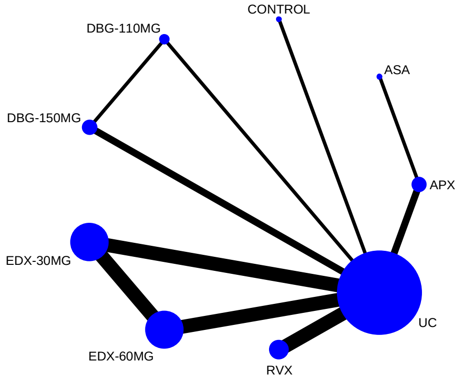

Clinically Relevant Non-Major Bleeding

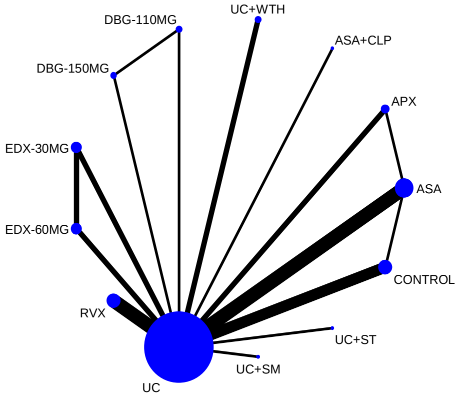

Intracranial Bleeding

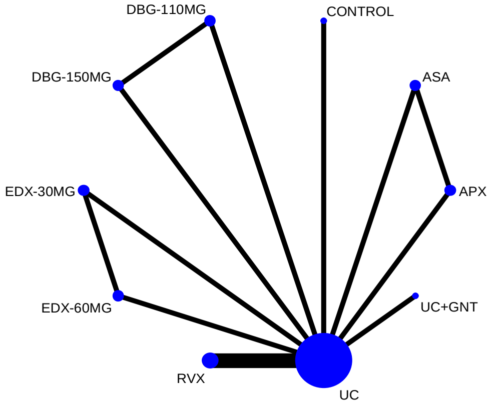

Gastrointestinal Bleeding

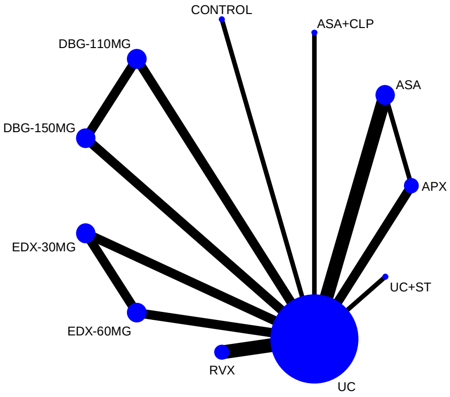

Myocardial Infarction

Abbreviations: APX, apixaban; ASA, aspirin; CLP, clopidogrel; DBG-110MG, dabigatran 110mg; DBG-150MG, dabigatran 150mg; EDX-30MG, edoxaban 30mg; EDX-60MG, edoxaban 60mg; GNT, genotype-guided warfarin dosing; RVX, rivaroxaban; SM: self-management of warfarin; ST: self-testing of warfarin; WTH: Watchman device

S7 Appendix: Assessment of Inconsistency

eTable 7.1: Evaluation of the global inconsistency in network using the 'design-by-treatment' interaction model for each outcome.

Network outcome	Chi-square	P-value test for global inconsistency
**Primary Outcome**		
• Stroke or Systemic Embolism	4.23	0.5169
• All-cause Mortality	0.60	0.7407
• Major Bleeding	0.90	0.6382
**Secondary Outcome**		
• Ischemic stroke	0.34	0.5598
• Clinically relevant non-major bleeding (CRNMB)	0.27	0.6042
• Intracranial bleeding	1.03	0.5956
• Gastrointestinal bleeding	1.10	0.2936
• Myocardial infarction	0	0.9562

S8 Appendix: Results of meta-analyses of direct comparisons of intervention options

eTable 8.1: Pairwise meta-analysis risk ratio (and 95% CI) for all dichotomous outcomes

Comparisons	vs.	Intervention	No. studies	No. of patients	Pairwise meta-analysis risk ratio (95% CI)		Heterogeneity I_2 (variation in RR attributable to heterogeneity)
					Fixed Effect	Random Effect	
**Stroke or Systemic Embolism**							
ASA	vs.	APX	1	5599	0.45 (0.32,0.62)	0.45 (0.32,0.62)	.
UC	vs.	APX	2	18419	0.78 (0.66,0.94)	0.40 (0.05,3.27)	59.20%
CONTROL	vs.	ASA	2	1543	1.05 (0.74,1.47)	1.05 (0.74,1.47)	0.00%
UC	vs.	ASA	7	2828	1.64 (1.27,2.12)	1.69 (1.00,2.83)	65.20%
UC	vs.	ASA+CLP	1	6706	1.85 (1.37,2.51)	1.85 (1.37,2.51)	.
UC	vs.	CONTROL	5	2329	2.69 (1.94,3.74)	2.61 (1.80,3.80)	14.00%
DBG-150MG	vs.	DBG-110MG	1	12195	1.37 (1.10,1.71)	1.37 (1.10,1.71)	.
UC	vs.	DBG-110MG	2	12135	0.91 (0.75,1.11)	0.91 (0.75,1.11)	0.00%
UC	vs.	DBG-150MG	2	12444	0.66 (0.54,0.82)	0.67 (0.54,0.83)	0.00%
EDX-60MG	vs.	EDX-30MG	2	14959	1.31 (1.13,1.52)	1.31 (1.13,1.52)	0.00%
UC	vs.	EDX-30MG	2	14969	1.14 (0.99,1.31)	0.84 (0.26,2.70)	41.50%
UC	vs.	EDX-60MG	2	14970	0.86 (0.74,1.01)	0.81 (0.47,1.40)	10.70%
UC	vs.	RVX	5	16045	0.85 (0.74,0.98)	0.86 (0.75,0.99)	0.00%
UC+GNT	vs.	UC	1	455	2.99 (0.12,72.94)	2.99 (0.12,72.94)	.
UC+SM	vs.	UC	2	1056	4.79 (1.75,13.11)	4.76 (1.74,13.06)	0.00%
UC+ST	vs.	UC	1	3001	1.01 (0.61,1.65)	1.01 (0.61,1.65)	.
UC+WTH	vs.	UC	2	1114	1.11 (0.66,1.87)	0.84 (0.21,3.37)	50.80%

**All-cause Mortality**							
ASA	vs.	APX	1	5599	0.79 (0.62,1.01)	0.79 (0.62,1.01)	.
UC	vs.	APX	1	18419	0.90 (0.81,1.00)	0.90 (0.81,1.00)	.
CONTROL	vs.	ASA	1	871	1.16 (0.48,2.83)	1.16 (0.48,2.83)	.
UC	vs.	ASA	6	3028	1.03 (0.86,1.23)	1.03 (0.86,1.23)	0.00%
UC	vs.	ASA+CLP	1	6706	1.00 (0.80,1.23)	1.00 (0.80,1.23)	.
UC	vs.	CONTROL	2	860	1.15 (0.81,1.65)	1.15 (0.80,1.65)	0.00%
DBG-150MG	vs.	DBG-110MG	1	12195	1.03 (0.91,1.17)	1.03 (0.91,1.17)	.
UC	vs.	DBG-110MG	1	12135	0.92 (0.81,1.04)	0.92 (0.81,1.04)	.
UC	vs.	DBG-150MG	1	12208	0.89 (0.79,1.01)	0.89 (0.79,1.01)	.
EDX-60MG	vs.	EDX-30MG	1	14069	0.95 (0.87,1.05)	0.95 (0.87,1.05)	.
UC	vs.	EDX-30MG	1	14070	0.88 (0.80,0.97)	0.88 (0.80,0.97)	.
UC	vs.	EDX-60MG	1	14071	0.92 (0.84,1.01)	0.92 (0.84,1.01)	.
UC	vs.	RVX	2	15664	0.85 (0.71,1.01)	0.85 (0.71,1.01)	0.00%
UC+GNT	vs.	UC	1	455	0.40 (0.08,2.03)	0.40 (0.08,2.03)	.
UC+SM	vs.	UC	1	854	2.51 (0.98,6.39)	2.51 (0.98,6.39)	.
UC+ST	vs.	UC	1	2922	1.04 (0.84,1.28)	1.04 (0.84,1.28)	.
UC+WTH	vs.	UC	2	1114	1.99 (1.15,3.43)	1.70 (0.62,4.63)	51.70%
**Major Bleeding**							
ASA	vs.	APX	1	5599	1.12 (0.73,1.72)	1.12 (0.73,1.72)	.
UC	vs.	APX	2	18419	0.70 (0.61,0.81)	0.70 (0.61,0.81)	0.00%
UC	vs.	ASA	3	616	0.65 (0.24,1.77)	0.72 (0.12,4.26)	49.50%
UC	vs.	ASA+CLP	1	6706	1.07 (0.81,1.42)	1.07 (0.81,1.42)	.

UC	vs.	CONTROL	2	798	0.28 (0.06,1.36)	0.30 (0.06,1.47)	0.00%
DBG-150MG	vs.	DBG-110MG	1	12091	0.87 (0.75,1.00)	0.87 (0.75,1.00)	.
UC	vs.	DBG-110MG	1	12037	0.81 (0.70,0.94)	0.81 (0.70,0.94)	.
UC	vs.	DBG-150MG	2	12334	0.94 (0.82,1.08)	0.94 (0.82,1.08)	0.00%
EDX-60MG	vs.	EDX-30MG	3	14959	0.60 (0.52,0.70)	0.61 (0.52,0.71)	0.00%
UC	vs.	EDX-30MG	3	14969	0.48 (0.42,0.56)	0.48 (0.42,0.56)	0.00%
UC	vs.	EDX-60MG	4	14970	0.80 (0.71,0.91)	0.80 (0.71,0.90)	0.00%
UC	vs.	RVX	5	16110	1.00 (0.88,1.14)	0.96 (0.67,1.38)	29.60%
UC+GNT	vs.	UC	N/A	455	N/A	N/A	.
UC+SM	vs.	UC	1	117	0.49 (0.05,5.27)	0.49 (0.05,5.27)	.
UC+WTH	vs.	UC	1	707	1.55 (0.85,2.84)	1.55 (0.85,2.84)	.
**Clinically Relevant Non-Major Bleeding**							
ASA	vs.	APX	1	5599	1.14 (0.85,1.52)	1.14 (0.85,1.52)	.
UC	vs.	APX	2	18419	0.68 (0.59,0.79)	0.68 (0.59,0.79)	0.00%
UC	vs.	CONTROL	1	421	0.33 (0.04,3.16)	0.33 (0.04,3.16)	.
DBG-150MG	vs.	DBG-110MG	1	104	0.50 (0.10,2.48)	0.50 (0.10,2.48)	.
UC	vs.	DBG-110MG	1	98	0.45 (0.09,2.22)	0.45 (0.09,2.22)	.
UC	vs.	DBG-150MG	2	346	1.14 (0.52,2.53)	1.13 (0.51,2.51)	0.00%
EDX-60MG	vs.	EDX-30MG	4	14959	0.79 (0.73,0.86)	0.77 (0.57,1.03)	8.40%
UC	vs.	EDX-30MG	4	14969	0.69 (0.64,0.75)	0.70 (0.65,0.75)	0.00%
UC	vs.	EDX-60MG	4	14970	0.88 (0.82,0.94)	0.87 (0.82,0.94)	0.00%
UC	vs.	RVX	4	15927	1.04 (0.97,1.12)	1.04 (0.97,1.11)	0.00%
**Ischemic Stroke**							

ASA	vs.	APX	1	5599	0.37 (0.26,0.55)	0.37 (0.26,0.55)	.
UC	vs.	APX	2	18419	0.91 (0.74,1.12)	0.54 (0.09,3.38)	49.10%
UC	vs.	ASA	4	1853	3.14 (1.87,5.27)	2.99 (1.77,5.06)	0.00%
UC	vs.	ASA+CLP	1	6706	2.12 (1.47,3.05)	2.12 (1.47,3.05)	.
UC	vs.	CONTROL	3	1280	2.97 (1.90,4.64)	2.86 (1.83,4.49)	0.00%
DBG-150MG	vs.	DBG-110MG	1	12091	1.45 (1.14,1.84)	1.45 (1.14,1.84)	.
UC	vs.	DBG-110MG	1	12037	1.12 (0.90,1.40)	1.12 (0.90,1.40)	.
UC	vs.	DBG-150MG	1	12098	0.78 (0.61,0.99)	0.78 (0.61,0.99)	.
EDX-60MG	vs.	EDX-30MG	1	14069	1.41 (1.20,1.66)	1.41 (1.20,1.66)	.
UC	vs.	EDX-30MG	1	14070	1.42 (1.20,1.67)	1.42 (1.20,1.67)	.
UC	vs.	EDX-60MG	1	14071	1.00 (0.84,1.20)	1.00 (0.84,1.20)	.
UC	vs.	RVX	4	15957	0.87 (0.72,1.05)	0.86 (0.69,1.07)	7.00%
UC+WTH	vs.	UC	2	1114	0.72 (0.37,1.43)	0.74 (0.37,1.46)	0.00%
**Intracranial Bleeding**							
ASA	vs.	APX	1	5599	0.84 (0.38,1.87)	0.84 (0.38,1.87)	.
UC	vs.	APX	2	18419	0.42 (0.31,0.58)	0.42 (0.31,0.58)	0.00%
CONTROL	vs.	ASA	1	871	2.09 (0.39,11.35)	2.09 (0.39,11.35)	.
UC	vs.	ASA	4	2082	0.67 (0.27,1.67)	0.68 (0.27,1.70)	0.00%
UC	vs.	ASA+CLP	1	6706	0.52 (0.25,1.07)	0.52 (0.25,1.07)	.
UC	vs.	CONTROL	4	1658	0.83 (0.24,2.85)	0.83 (0.22,3.17)	0.00%
DBG-150MG	vs.	DBG-110MG	1	12091	0.76 (0.46,1.25)	0.76 (0.46,1.25)	.
UC	vs.	DBG-110MG	1	12037	0.31 (0.20,0.48)	0.31 (0.20,0.48)	.

UC	vs.	DBG-150MG	1	12098	0.41 (0.28,0.60)	0.41 (0.28,0.60)	.
EDX-60MG	vs.	EDX-30MG	2	14331	0.66 (0.45,0.98)	0.67 (0.45,0.98)	0.00%
UC	vs.	EDX-30MG	1	14330	0.31 (0.22,0.44)	0.31 (0.22,0.44)	.
UC	vs.	EDX-60MG	2	14331	0.47 (0.35,0.64)	0.58 (0.18,1.87)	22.30%
UC	vs.	RVX	5	16110	0.73 (0.56,0.94)	0.76 (0.53,1.08)	22.20%
UC+SM	vs.	UC	1	737	1.00 (0.14,7.08)	1.00 (0.14,7.08)	.
UC+ST	vs.	UC	1	2922	0.73 (0.30,1.81)	0.73 (0.30,1.81)	.
UC+WTH	vs.	UC	2	1114	4.45 (1.48,13.36)	3.30 (0.44,24.86)	40.80%
**Gastrointestinal Bleeding**							
ASA	vs.	APX	1	5599	0.85 (0.40,1.84)	0.85 (0.40,1.84)	.
UC	vs.	APX	1	18201	0.88 (0.68,1.14)	0.88 (0.68,1.14)	.
UC	vs.	ASA	1	440	0.20 (0.02,1.63)	0.20 (0.02,1.63)	.
UC	vs.	CONTROL	1	439	0.23 (0.05,1.07)	0.23 (0.05,1.07)	.
DBG-150MG	vs.	DBG-110MG	1	12091	0.74 (0.59,0.92)	0.74 (0.59,0.92)	.
UC	vs.	DBG-110MG	1	12037	1.11 (0.87,1.42)	1.11 (0.87,1.42)	.
UC	vs.	DBG-150MG	1	12098	1.50 (1.20,1.89)	1.50 (1.20,1.89)	.
EDX-60MG	vs.	EDX-30MG	1	14069	0.56 (0.45,0.69)	0.56 (0.45,0.69)	.
UC	vs.	EDX-30MG	1	14070	0.68 (0.54,0.85)	0.68 (0.54,0.85)	.
UC	vs.	EDX-60MG	1	14071	1.22 (1.01,1.48)	1.22 (1.01,1.48)	.
UC	vs.	RVX	3	15867	1.43 (1.17,1.74)	1.33 (0.48,3.70)	72.00%
UC+GNT	vs.	UC	1	455	1.36 (0.64,2.89)	1.36 (0.64,2.89)	.
**Myocardial Infarction**							
ASA	vs.	APX	1	5599	0.85 (0.50,1.47)	0.85 (0.50,1.47)	.

UC	vs.	APX	1	18419	0.88 (0.66,1.17)	0.88 (0.66,1.17)	.
UC	vs.	ASA	3	1413	1.06 (0.59,1.90)	1.06 (0.59,1.90)	0.00%
UC	vs.	ASA+CLP	1	6706	1.55 (0.92,2.61)	1.55 (0.92,2.61)	.
UC	vs.	CONTROL	1	421	1.00 (0.14,7.00)	1.00 (0.14,7.00)	.
DBG-150MG	vs.	DBG-110MG	1	12195	0.98 (0.73,1.31)	0.98 (0.73,1.31)	.
UC	vs.	DBG-110MG	1	12135	1.37 (0.99,1.89)	1.37 (0.99,1.89)	.
UC	vs.	DBG-150MG	1	12208	1.40 (1.02,1.93)	1.40 (1.02,1.93)	.
EDX-60MG	vs.	EDX-30MG	2	14538	1.27 (1.01,1.58)	1.27 (1.01,1.58)	0.00%
UC	vs.	EDX-30MG	2	14555	1.21 (0.97,1.51)	1.21 (0.97,1.51)	0.00%
UC	vs.	EDX-60MG	2	14555	0.96 (0.76,1.21)	1.13 (0.40,3.21)	20.10%
UC	vs.	RVX	3	15481	0.82 (0.64,1.06)	0.82 (0.63,1.06)	0.00%
UC+ST	vs.	UC	1	2922	0.67 (0.37,1.21)	0.67 (0.37,1.21)	.

Abbreviations: APX, apixaban; ASA, aspirin; CLP, clopidogrel; DBG-110MG, dabigatran 110mg; DBG-150MG, dabigatran 150mg; EDX-30MG, edoxaban 30mg; EDX-60MG, edoxaban 60mg; GNT, genotype-guided warfarin dosing; RVX, rivaroxaban; SM: self-management of warfarin; ST: self-testing of warfarin; WTH: Watchman device

S9 Appendix: Results of Network Meta-analysis

Intervention options are in order of their efficacy or safety ranking. Estimates are presented as risk ratios (RR) and 95% confidence intervals. Treatments are ordered by rankings for each outcome.

Comparisons between interventions should be read from column to row for each outcome (row intervention is reference). Risk ratios less than 1 favour the column-defining treatment. To obtain risks ratios for comparisons in the opposite direction, reciprocals should be taken. Significant results are in bold and underlined. Blue-colour box represents that unfavourable outcome was decreased. In contrast, red-colour box represents that unfavourable outcome was increased.

eTable 9.1: Results of network meta-analysis of interventions options on stroke or systemic embolism

The following table shows the effect sizes (risk ratio) and the rank order (SUCRA ranks) compared to usual warfarin care (UC).

Interventions	Risk ratio	95% Confidence interval	p-value	SUCRA rank
UC+SM	**0.24**	**(0.08, 0.68)**	**0.007**	1
EDX-30MG	1.06	(0.65, 1.72)	0.820	2
UC+GNT	0.33	(0.13, 8.45)	0.506	3
ASA	**1.72**	**(1.29, 2.29)**	**0.000**	4
UC	reference			5
RVX	0.79	(0.57, 1.10)	0.166	6
UC+WTH	0.89	(0.45, 1.78)	0.748	7
DBG-150MG	0.65	(0.39, 1.07)	0.089	8
UC+ST	0.99	(0.51, 1.93)	0.986	9
APX	0.76	(0.51, 1.12)	0.169	10
EDX-60MG	0.82	(0.51, 1.32)	0.410	11
ASA+CLP	**1.85**	**(1.07, 3.21)**	**0.028**	12
CONTROL	**2.12**	**(1.49, 3.01)**	**0.000**	13
DBG-110MG	0.89	(0.54, 1.46)	0.647	14
**Number of RCTs**	**36**			

Abbreviations: APX, apixaban; ASA, aspirin; CLP, clopidogrel; DBG-110MG, dabigatran 110mg; DBG-150MG, dabigatran 150mg; EDX-30MG, edoxaban 30mg; EDX-60MG, edoxaban 60mg; GNT, genotype-guided warfarin dosing; RVX, rivaroxaban; SM: self-management of warfarin; ST: self-testing of warfarin; WTH: Watchman device

eFigure 9.1: Network estimated risk ratios (95% confidence intervals) of intervention options on stroke or systemic embolism

**UC+SM**													
0.22 (0.07,0.72)	**EDX-30MG**												
0.71 (0.02,21.08)	3.16 (0.12,82.72)	**UC+GNT**											
0.14 (0.05,0.41)	0.61 (0.35,1.08)	0.19 (0.01,4.97)	**ASA**										
0.24 (0.08,0.68)	1.06 (0.65,1.72)	0.33 (0.01,8.45)	1.72 (1.29,2.29)	**UC**									
0.30 (0.10,0.90)	1.33 (0.77,2.33)	0.42 (0.02,10.84)	2.17 (1.40,3.36)	1.26 (0.91,1.75)	**RVX**								
0.26 (0.08,0.93)	1.18 (0.50,2.82)	0.37 (0.01,10.17)	1.93 (0.91,4.06)	1.12 (0.56,2.23)	0.89 (0.41,1.94)	**UC+WTH**							
0.37 (0.11,1.17)	1.63 (0.82,3.24)	0.52 (0.02,13.56)	2.66 (1.49,4.73)	1.54 (0.94,2.55)	1.22 (0.68,2.20)	1.38 (0.58,3.26)	**DBG-150MG**						
0.24 (0.07,0.83)	1.06 (0.47,2.42)	0.34 (0.01,9.09)	1.73 (0.84,3.57)	1.01 (0.52,1.95)	0.80 (0.38,1.67)	0.90 (0.35,2.34)	0.65 (0.28,1.50)	**UC+ST**					
0.31 (0.10,0.96)	1.40 (0.76,2.57)	0.44 (0.02,11.41)	2.27 (1.51,3.42)	1.32 (0.89,1.95)	1.05 (0.64,1.72)	1.18 (0.53,2.63)	0.85 (0.45,1.60)	1.31 (0.61,2.84)	**APX**				
0.29 (0.09,0.93)	1.30 (0.81,2.07)	0.41 (0.02,10.72)	2.11 (1.20,3.69)	1.22 (0.76,1.98)	0.97 (0.56,1.69)	1.09 (0.46,2.58)	0.79 (0.40,1.57)	1.22 (0.54,2.76)	0.93 (0.51,1.70)	**EDX-60MG**			
0.13 (0.04,0.42)	0.57 (0.27,1.19)	0.18 (0.01,4.78)	0.93 (0.50,1.73)	0.54 (0.31,0.94)	**0.43 (0.23,0.81)**	0.48 (0.20,1.16)	0.35 (0.17,0.73)	0.54 (0.23,1.27)	0.41 (0.21,0.80)	0.44 (0.21,0.92)	**ASA+CLP**		
0.11 (0.04,0.34)	0.50 (0.27,0.92)	0.16 (0.01,4.06)	0.81 (0.56,1.17)	0.47 (0.33,0.67)	**0.37 (0.23,0.61)**	0.42 (0.20,0.90)	0.31 (0.16,0.57)	0.47 (0.22,0.99)	0.36 (0.22,0.59)	0.39 (0.21,0.71)	0.87 (0.46,1.68)	**CONTROL**	
0.27 (0.08,0.85)	1.19 (0.60,2.35)	0.38 (0.01,9.85)	1.93 (1.09,3.42)	1.12 (0.68,1.84)	0.89 (0.50,1.60)	1.00 (0.43,2.36)	0.73 (0.44,1.20)	1.12 (0.49,2.56)	0.85 (0.45,1.59)	0.92 (0.46,1.81)	2.08 (0.99,4.36)	2.38 (1.29,4.39)	**DBG-110MG**

*** Treatments are ordered by SUCRA rank. Comparisons between treatments should be read from column to row for each outcome (row treatment is reference).**

Abbreviations: APX, apixaban; ASA, aspirin; CLP, clopidogrel; DBG-110MG, dabigatran 110mg; DBG-150MG, dabigatran 150mg; EDX-30MG, edoxaban 30mg; EDX-60MG, edoxaban 60mg; GNT, genotype-guided warfarin dosing; RVX, rivaroxaban; SM: self-management of warfarin; ST: self-testing of warfarin; WTH: Watchman device

eTable 9.2: Results of network meta-analysis of interventions options on major bleeding

The following table shows the effect sizes (risk ratio) and the rank order (SUCRA ranks) compared to usual warfarin care (UC).

Interventions	Risk ratio	95% Confidence interval	p-value	SUCRA rank
EDX-60MG	**0.80**	**(0.71, 0.90)**	**0.000**	1
DBG-110MG	**0.81**	**(0.71, 0.94)**	**0.004**	2
ASA+CLP	1.07	(0.81, 1.42)	0.612	3
ASA	**0.63**	**(0.41, 0.96)**	**0.030**	4
UC+WTH	0.64	(0.35, 1.18)	0.153	5
RVX	1.01	(0.89, 1.16)	0.840	6
DBG-150MG	0.94	(0.82, 1.08)	0.367	7
UC+GNT	1.00	(0.02, 50.40)	0.998	8
EDX-30MG	**0.48**	**(0.42, 0.56)**	**0.000**	9
APX	**0.70**	**(0.61, 0.81)**	**0.000**	10
UC+SM	2.03	(0.19, 21.83)	0.557	11
UC	reference			12
CONTROL	0.30	(0.06, 1.47)	0.138	13
**Number of RCTs**	**23**			

Abbreviations: APX, apixaban; ASA, aspirin; CLP, clopidogrel; DBG-110MG, dabigatran 110mg; DBG-150MG, dabigatran 150mg; EDX-30MG, edoxaban 30mg; EDX-60MG, edoxaban 60mg; GNT, genotype-guided warfarin dosing; RVX, rivaroxaban; SM: self-management of warfarin; ST: self-testing of warfarin; WTH: Watchman device

eFigure 9.2: Network estimated risk ratios (95% confidence intervals) of intervention options on major bleeding

**EDX-60MG**												
0.98 (0.81,1.19)	**DBG-110MG**											
0.74 (0.55,1.01)	0.76 (0.55,1.03)	**ASA+CLP**										
1.28 (0.82,1.98)	1.30 (0.83,2.03)	**1.71 (1.03,2.84)**	**ASA**									
1.24 (0.67,2.30)	1.26 (0.68,2.35)	1.67 (0.86,3.24)	0.97 (0.47,2.03)	**UC+WTH**								
**0.79 (0.66,0.94)**	**0.80 (0.66,0.97)**	1.06 (0.78,1.44)	**0.62 (0.40,0.96)**	0.64 (0.34,1.18)	**RVX**							
0.85 (0.71,1.02)	0.87 (0.75,1.00)	1.14 (0.84,1.56)	0.67 (0.43,1.04)	0.69 (0.37,1.27)	1.08 (0.89,1.30)	**DBG-150MG**						
0.80 (0.02,40.01)	0.81 (0.02,40.74)	1.07 (0.02,54.21)	0.62 (0.01,32.03)	0.64 (0.01,33.70)	1.01 (0.02,50.75)	0.93 (0.02,47.03)	**UC+GNT**					
**1.65 (1.42,1.92)**	**1.68 (1.37,2.06)**	**2.22 (1.62,3.04)**	1.30 (0.83,2.03)	1.33 (0.72,2.48)	**2.10 (1.72,2.55)**	**1.94 (1.59,2.37)**	2.08 (0.04,104.57)	**EDX-30MG**				
1.14 (0.94,1.37)	1.16 (0.95,1.41)	**1.53 (1.12,2.08)**	0.89 (0.60,1.34)	0.92 (0.49,1.70)	**1.44 (1.19,1.74)**	**1.34 (1.10,1.62)**	1.43 (0.03,71.88)	**0.69 (0.56,0.84)**	**APX**			
0.39 (0.04,4.23)	0.40 (0.04,4.31)	0.53 (0.05,5.76)	0.31 (0.03,3.43)	0.32 (0.03,3.66)	0.50 (0.05,5.36)	0.46 (0.04,4.97)	0.49 (0.01,48.07)	0.24 (0.02,2.56)	0.35 (0.03,3.72)	**UC+SM**		
**0.80 (0.71,0.90)**	**0.81 (0.71,0.94)**	1.07 (0.81,1.42)	**0.63 (0.41,0.96)**	0.64 (0.35,1.18)	1.01 (0.89,1.16)	0.94 (0.82,1.08)	1.00 (0.02,50.40)	**0.48 (0.42,0.56)**	**0.70 (0.61,0.81)**	2.03 (0.19,21.83)	**UC**	
2.67 (0.54,13.21)	2.72 (0.55,13.46)	3.59 (0.71,18.10)	2.09 (0.40,10.89)	2.15 (0.39,11.83)	3.39 (0.68,16.76)	3.14 (0.63,15.54)	3.36 (0.05,230.02)	1.61 (0.33,8.00)	2.35 (0.47,11.63)	6.80 (0.39,118.51)	3.34 (0.68,16.45)	**CONTROL**

*** Treatments are ordered by SUCRA rank. Comparisons between treatments should be read from column to row for each outcome (row treatment is reference).**

Abbreviations: APX, apixaban; ASA, aspirin; CLP, clopidogrel; DBG-110MG, dabigatran 110mg; DBG-150MG, dabigatran 150mg; EDX-30MG, edoxaban 30mg; EDX-60MG, edoxaban 60mg; GNT, genotype-guided warfarin dosing; RVX, rivaroxaban; SM: self-management of warfarin; ST: self-testing of warfarin; WTH: Watchman device

eTable 9.3: Results of network meta-analysis of interventions options on all-cause mortality

The following table shows the effect sizes (risk ratio) and the rank order (SUCRA ranks) compared to usual warfarin care (UC).

Interventions	Risk ratio	95% Confidence interval	p-value	SUCRA rank
UC+WTH	**0.49**	**(0.28, 0.86)**	**0.012**	1
UC+SM	0.42	(0.17, 1.04)	0.061	2
UC				3
EDX-60MG	0.92	(0.84, 1.01)	0.081	4
ASA	1.07	(0.92, 1.24)	0.386	5
EDX-30MG	**0.88**	**(0.80, 0.96)**	**0.007**	6
DBG-150MG	0.89	(0.79, 1.01)	0.069	7
RVX	0.85	(0.71, 1.01)	0.065	8
UC+ST	0.96	(0.78, 1.19)	0.725	9
CONTROL	1.11	(0.80, 1.56)	0.522	10
APX	**0.89**	**(0.80, 0.98)**	**0.020**	11
DBG-110MG	0.92	(0.81, 1.04)	0.169	12
ASA+CLP	1.00	(0.80, 1.23)	0.968	13
UC+GNT	2.51	(0.49, 12.81)	0.268	14
**Number of RCTs**	**26**			

Abbreviations: APX, apixaban; ASA, aspirin; CLP, clopidogrel; DBG-110MG, dabigatran 110mg; DBG-150MG, dabigatran 150mg; EDX-30MG, edoxaban 30mg; EDX-60MG, edoxaban 60mg; GNT, genotype-guided warfarin dosing; RVX, rivaroxaban; SM: self-management of warfarin; ST: self-testing of warfarin; WTH: Watchman device

eFigure 9.3: Network estimated risk ratios (95% confidence intervals) of intervention options on all-cause mortality

**UC+WTH**													
1.17 (0.40,3.40)	**UC+SM**												
**0.49 (0.28,0.86)**	0.42 (0.17,1.04)	**UC**											
**0.53 (0.30,0.94)**	0.46 (0.18,1.14)	1.09 (0.99,1.19)	**EDX-60MG**										
**0.46 (0.26,0.82)**	**0.39 (0.16,0.99)**	0.94 (0.81,1.09)	0.86 (0.73,1.03)	**ASA**									
**0.56 (0.32,0.98)**	0.48 (0.19,1.19)	1.14 (1.04,1.25)	1.05 (0.95,1.15)	**1.21 (1.02,1.45)**	**EDX-30MG**								
**0.55 (0.31,0.98)**	0.47 (0.19,1.18)	1.12 (0.99,1.27)	1.03 (0.89,1.21)	1.20 (0.99,1.45)	0.99 (0.84,1.15)	**DBG-150MG**							
0.58 (0.32,1.04)	0.50 (0.20,1.25)	1.18 (0.99,1.41)	1.09 (0.89,1.33)	**1.26 (1.00,1.59)**	1.04 (0.85,1.27)	1.05 (0.85,1.31)	**RVX**						
**0.51 (0.28,0.93)**	0.44 (0.17,1.11)	1.04 (0.84,1.28)	0.96 (0.76,1.20)	1.11 (0.86,1.43)	0.91 (0.72,1.15)	0.93 (0.72,1.18)	0.88 (0.67,1.16)	**UC+ST**					
**0.44 (0.23,0.84)**	**0.38 (0.14,0.99)**	0.90 (0.64,1.25)	0.83 (0.58,1.17)	0.96 (0.67,1.37)	0.79 (0.56,1.11)	0.80 (0.56,1.14)	0.76 (0.52,1.11)	0.86 (0.58,1.28)	**CONTROL**				
**0.55 (0.31,0.97)**	0.47 (0.19,1.18)	**1.13 (1.02,1.24)**	1.04 (0.91,1.19)	1.20 (1.03,1.41)	0.99 (0.86,1.13)	1.00 (0.86,1.18)	0.95 (0.78,1.17)	1.08 (0.86,1.37)	1.26 (0.89,1.78)	**APX**			
**0.54 (0.30,0.95)**	0.46 (0.18,1.15)	1.09 (0.96,1.23)	1.00 (0.86,1.17)	1.16 (0.96,1.41)	0.96 (0.82,1.12)	0.97 (0.86,1.10)	0.92 (0.74,1.15)	1.05 (0.82,1.34)	1.22 (0.85,1.73)	0.97 (0.83,1.13)	**DBG-110MG**		
**0.49 (0.27,0.90)**	0.42 (0.17,1.07)	1.00 (0.81,1.25)	0.93 (0.73,1.17)	1.07 (0.83,1.39)	0.88 (0.70,1.12)	0.90 (0.70,1.15)	0.85 (0.64,1.12)	0.97 (0.72,1.31)	1.12 (0.75,1.67)	0.89 (0.70,1.13)	0.92 (0.72,1.18)	**ASA+CLP**	
0.20 (0.03,1.10)	0.17 (0.03,1.08)	0.40 (0.08,2.03)	0.37 (0.07,1.88)	0.43 (0.08,2.18)	0.35 (0.07,1.79)	0.35 (0.07,1.82)	0.34 (0.07,1.73)	0.38 (0.07,1.98)	0.44 (0.08,2.34)	0.35 (0.07,1.81)	0.37 (0.07,1.87)	0.40 (0.08,2.05)	**UC+GNT**

*** Treatments are ordered by SUCRA rank. Comparisons between treatments should be read from column to row for each outcome (row treatment is reference).**

Abbreviations: APX, apixaban; ASA, aspirin; CLP, clopidogrel; DBG-110MG, dabigatran 110mg; DBG-150MG, dabigatran 150mg; EDX-30MG, edoxaban 30mg; EDX-60MG, edoxaban 60mg; GNT, genotype-guided warfarin dosing; RVX, rivaroxaban; SM: self-management of warfarin; ST: self-testing of warfarin; WTH: Watchman device

eTable 9.4: Results of network meta-analysis of interventions options on ischemic stroke

The following table shows the effect sizes (risk ratio) and the rank order (SUCRA ranks) compared to usual warfarin care (UC).

Interventions	Risk ratio	95% Confidence interval	p-value	SUCRA rank
EDX-30MG	**1.42**	**(1.20, 1.67)**	**0.000**	1
UC	reference			2
ASA	**2.65**	**(1.89, 3.71)**	**0.000**	3
RVX	0.87	(0.72, 1.05)	0.155	4
APX	0.93	(0.76, 1.14)	0.478	5
DBG-150MG	**0.77**	**(0.61, 0.99)**	**0.042**	6
UC+WTH	1.36	(0.69, 2.63)	0.378	7
EDX-60MG	1.00	(0.84, 1.20)	0.961	8
CONTROL	**2.86**	**(1.83, 4.49)**	**0.000**	9
ASA+CLP	**2.12**	**(1.47, 3.05)**	**0.000**	10
DBG-110MG	1.12	(0.90, 1.40)	0.316	11
**Number of RCTs**	**19**			

Abbreviations: APX, apixaban; ASA, aspirin; CLP, clopidogrel; DBG-110MG, dabigatran 110mg; DBG-150MG, dabigatran 150mg; EDX-30MG, edoxaban 30mg; EDX-60MG, edoxaban 60mg; GNT, genotype-guided warfarin dosing; RVX, rivaroxaban; SM: self-management of warfarin; ST: self-testing of warfarin; WTH: Watchman device

eFigure 9.4: Network estimated risk ratios (95% confidence intervals) of intervention options on ischemic stroke

**EDX-30MG**										
**1.42 (1.20,1.67)**	**UC**									
**0.53 (0.37,0.78)**	**0.38 (0.27,0.53)**	**ASA**								
**1.63 (1.27,2.09)**	1.15 (0.95,1.39)	**3.04 (2.07,4.47)**	**RVX**							
**1.52 (1.18,1.98)**	1.08 (0.88,1.31)	**2.85 (2.07,3.92)**	0.94 (0.71,1.24)	**APX**						
**1.83 (1.36,2.46)**	**1.29 (1.01,1.65)**	**3.42 (2.26,5.19)**	1.13 (0.83,1.53)	1.20 (0.87,1.65)	**DBG-150MG**					
1.04 (0.52,2.11)	0.74 (0.37,1.46)	1.95 (0.91,4.18)	0.64 (0.32,1.30)	0.68 (0.34,1.39)	0.57 (0.28,1.18)	**UC+WTH**				
**1.41 (1.20,1.66)**	1.00 (0.83,1.19)	**2.64 (1.80,3.86)**	0.87 (0.67,1.13)	0.93 (0.71,1.21)	0.77 (0.57,1.04)	1.35 (0.67,2.74)	**EDX-60MG**			
**0.49 (0.31,0.80)**	**0.35 (0.22,0.55)**	0.93 (0.53,1.62)	**0.30 (0.19,0.50)**	**0.32 (0.20,0.53)**	**0.27 (0.16,0.45)**	0.47 (0.21,1.08)	**0.35 (0.22,0.57)**	**CONTROL**		
0.67 (0.45,1.00)	**0.47 (0.33,0.68)**	1.25 (0.76,2.05)	**0.41 (0.27,0.62)**	**0.44 (0.29,0.66)**	**0.37 (0.24,0.57)**	0.64 (0.30,1.39)	**0.47 (0.32,0.71)**	1.35 (0.76,2.41)	**ASA+CLP**	
1.26 (0.96,1.67)	0.89 (0.71,1.12)	**2.37 (1.58,3.54)**	0.78 (0.58,1.04)	0.83 (0.61,1.12)	**0.69 (0.54,0.88)**	1.21 (0.59,2.49)	0.90 (0.67,1.19)	**2.55 (1.55,4.22)**	**1.89 (1.23,2.90)**	**DBG-110MG**

*** Treatments are ordered by SUCRA rank. Comparisons between treatments should be read from column to row for each outcome (row treatment is reference).**

Abbreviations: APX, apixaban; ASA, aspirin; CLP, clopidogrel; DBG-110MG, dabigatran 110mg; DBG-150MG, dabigatran 150mg; EDX-30MG, edoxaban 30mg; EDX-60MG, edoxaban 60mg; GNT, genotype-guided warfarin dosing; RVX, rivaroxaban; SM: self-management of warfarin; ST: self-testing of warfarin; WTH: Watchman device

eTable 9.5: Results of network meta-analysis of interventions options on clinically relevant non-major bleeding

The following table shows the effect sizes (risk ratio) and the rank order (SUCRA ranks) compared to usual warfarin care (UC).

Interventions	Risk ratio	95% Confidence interval	p-value	SUCRA rank
CONTROL	0.33	(0.03, 3.16)	0.338	1
DBG-110MG	0.51	(0.11, 2.34)	0.385	2
DBG-150MG	1.13	(0.51, 2.51)	0.767	3
ASA	**0.60**	**(0.44, 0.83)**	**0.002**	4
EDX-60MG	**0.87**	**(0.82, 0.94)**	**0.000**	5
RVX	1.04	(0.97, 1.11)	0.295	6
APX	**0.68**	**(0.59, 0.79)**	**0.000**	7
EDX-30MG	**0.70**	**(0.65, 0.75)**	**0.000**	8
UC	reference			9
**Number of RCTs**	**14**			

Abbreviations: APX, apixaban; ASA, aspirin; CLP, clopidogrel; DBG-110MG, dabigatran 110mg; DBG-150MG, dabigatran 150mg; EDX-30MG, edoxaban 30mg; EDX-60MG, edoxaban 60mg; GNT, genotype-guided warfarin dosing; RVX, rivaroxaban; SM: self-management of warfarin; ST: self-testing of warfarin; WTH: Watchman device

eFigure 9.5: Network estimated risk ratios (95% confidence intervals) of intervention options on clinically relevant non-major bleeding

**CONTROL**								
**0.65 (0.04,9.99)**	**DBG-110MG**							
0.29 (0.03,3.22)	0.45 (0.10,2.08)	**DBG-150MG**						
0.55 (0.06,5.38)	0.84 (0.18,4.03)	1.88 (0.79,4.45)	**ASA**					
0.38 (0.04,3.62)	0.58 (0.13,2.69)	1.29 (0.58,2.88)	**0.69 (0.49,0.96)**	**EDX-60MG**				
0.32 (0.03,3.05)	0.49 (0.11,2.26)	1.09 (0.49,2.42)	**0.58 (0.42,0.81)**	**0.84 (0.76,0.93)**	**RVX**			
0.49 (0.05,4.65)	0.74 (0.16,3.46)	1.65 (0.73,3.72)	0.88 (0.66,1.17)	**1.28 (1.09,1.51)**	**1.52 (1.29,1.79)**	**APX**		
0.48 (0.05,4.56)	0.73 (0.16,3.38)	1.62 (0.73,3.62)	0.86 (0.62,1.21)	**1.26 (1.16,1.36)**	**1.49 (1.35,1.66)**	0.98 (0.83,1.16)	**EDX-30MG**	
0.33 (0.03,3.16)	0.51 (0.11,2.34)	1.13 (0.51,2.51)	**0.60 (0.44,0.83)**	**0.87 (0.82,0.94)**	1.04 (0.97,1.11)	**0.68 (0.59,0.79)**	**0.70 (0.65,0.75)**	**UC**

*** Treatments are ordered by SUCRA rank. Comparisons between treatments should be read from column to row for each outcome (row treatment is reference).**

Abbreviations: APX, apixaban; ASA, aspirin; CLP, clopidogrel; DBG-110MG, dabigatran 110mg; DBG-150MG, dabigatran 150mg; EDX-30MG, edoxaban 30mg; EDX-60MG, edoxaban 60mg; GNT, genotype-guided warfarin dosing; RVX, rivaroxaban; SM: self-management of warfarin; ST: self-testing of warfarin; WTH: Watchman device

eTable 9.6: Results of network meta-analysis of interventions options on intracranial bleeding

The following table shows the effect sizes (risk ratio) and the rank order (SUCRA ranks) compared to usual warfarin care (UC).

Interventions	Risk ratio	95% Confidence interval	p-value	SUCRA rank
UC+WTH	**0.22**	**0.22 (0.06, 0.76)**	**0.016**	1
EDX-60MG	**0.48**	**0.48 (0.30, 0.78)**	**0.003**	2
DBG-150MG	**0.41**	**0.41 (0.24, 0.70)**	**0.001**	3
EDX-30MG	**0.31**	**0.31 (0.19, 0.52)**	**0.000**	4
ASA	0.65	0.65 (0.34, 1.24)	0.199	5
RVX	0.76	0.76 (0.53, 1.08)	0.131	6
CONTROL	0.57	0.57 (0.19, 1.71)	0.316	7
DBG-110MG	**0.31**	**0.31 (0.18, 0.55)**	**0.000**	8
ASA+CLP	0.52	0.52 (0.23, 1.17)	0.114	9
UC+SM	1.00	(0.14, 7.29)	0.998	10
UC	reference			11
APX	**0.44**	**0.44 (0.28, 0.70)**	**0.000**	12
UC+ST	1.37	1.37 (0.51, 3.64)	0.531	13
**Number of RCTs**	**26**			

Abbreviations: APX, apixaban; ASA, aspirin; CLP, clopidogrel; DBG-110MG, dabigatran 110mg; DBG-150MG, dabigatran 150mg; EDX-30MG, edoxaban 30mg; EDX-60MG, edoxaban 60mg; GNT, genotype-guided warfarin dosing; RVX, rivaroxaban; SM: self-management of warfarin; ST: self-testing of warfarin; WTH: Watchman device

eFigure 9.6: Network estimated risk ratios (95% confidence intervals) of intervention options on intracranial bleeding

**UC+WTH**												
0.46 (0.12,1.71)	**EDX-60MG**											
0.54 (0.14,2.06)	1.17 (0.57,2.40)	**DBG-150MG**										
0.70 (0.19,2.65)	1.53 (0.90,2.61)	1.30 (0.63,2.71)	**EDX-30MG**									
0.34 (0.08,1.37)	0.74 (0.33,1.65)	0.63 (0.27,1.47)	0.48 (0.21,1.10)	**ASA**								
0.29 (0.08,1.05)	0.63 (0.35,1.16)	0.54 (0.28,1.02)	0.41 (0.22,0.77)	0.86 (0.40,1.81)	**RVX**							
0.39 (0.07,2.01)	0.84 (0.26,2.78)	0.72 (0.21,2.43)	0.55 (0.17,1.84)	1.14 (0.36,3.57)	1.33 (0.42,4.23)	**CONTROL**						
0.71 (0.18,2.76)	1.55 (0.74,3.25)	1.32 (0.71,2.45)	1.01 (0.48,2.16)	2.09 (0.88,4.98)	**2.45 (1.26,4.77)**	1.84 (0.53,6.31)	**DBG-110MG**					
0.43 (0.10,1.87)	0.93 (0.36,2.39)	0.79 (0.30,2.09)	0.61 (0.23,1.58)	1.26 (0.44,3.57)	1.47 (0.60,3.57)	1.10 (0.28,4.32)	0.60 (0.22,1.62)	**ASA+CLP**				
0.22 (0.02,2.30)	0.48 (0.06,3.73)	0.41 (0.05,3.22)	0.32 (0.04,2.45)	0.65 (0.08,5.29)	0.76 (0.10,5.75)	0.57 (0.06,5.55)	0.31 (0.04,2.46)	0.52 (0.06,4.46)	**UC+SM**			
**0.22 (0.06,0.76)**	**0.48 (0.30,0.78)**	**0.41 (0.24,0.70)**	**0.31 (0.19,0.52)**	0.65 (0.34,1.25)	0.76 (0.53,1.08)	0.57 (0.19,1.71)	**0.31 (0.18,0.55)**	0.52 (0.23,1.17)	1.00 (0.14,7.29)	**UC**		
0.50 (0.13,1.85)	1.09 (0.57,2.08)	0.93 (0.46,1.86)	0.71 (0.36,1.39)	1.47 (0.76,2.83)	1.72 (0.96,3.07)	1.29 (0.41,4.08)	0.70 (0.34,1.45)	1.17 (0.46,2.97)	2.25 (0.29,17.30)	**2.26 (1.43,3.55)**	**APX**	
**0.16 (0.03,0.78)**	0.35 (0.12,1.05)	**0.30 (0.10,0.91)**	**0.23 (0.08,0.69)**	0.48 (0.15,1.55)	0.56 (0.20,1.58)	0.42 (0.10,1.82)	**0.23 (0.07,0.70)**	0.38 (0.11,1.35)	0.73 (0.08,6.69)	0.73 (0.27,1.95)	0.32 (0.11,0.95)	**UC+ST**

*** Treatments are ordered by SUCRA rank. Comparisons between treatments should be read from column to row for each outcome (row treatment is reference).**

Abbreviations: APX, apixaban; ASA, aspirin; CLP, clopidogrel; DBG-110MG, dabigatran 110mg; DBG-150MG, dabigatran 150mg; EDX-30MG, edoxaban 30mg; EDX-60MG, edoxaban 60mg; GNT, genotype-guided warfarin dosing; RVX, rivaroxaban; SM: self-management of warfarin; ST: self-testing of warfarin; WTH: Watchman device

eTable 9.7: Results of network meta-analysis of interventions options on gastrointestinal bleeding

The following table shows the effect sizes (risk ratio) and the rank order (SUCRA ranks) compared to usual warfarin care (UC).

Interventions	Risk ratio	95% Confidence interval	p-value	SUCRA rank
DBG-110MG	1.11	(0.21, 5.87)	0.903	1
CONTROL	0.23	(0.02, 2.20)	0.204	2
ASA	0.48	(0.07, 3.28)	0.457	3
EDX-60MG	1.22	(0.23, 6.41)	0.813	4
UC+GNT	0.74	(0.12, 4.51)	0.741	5
APX	0.62	(0.13, 2.86)	0.541	6
DBG-150MG	1.50	(0.29, 7.93)	0.631	7
RVX	1.37	(0.42, 4.43)	0.601	8
UC	reference			9
EDX-30MG	0.68	(0.13, 3.58)	0.648	10
**Number of RCTs**	**10**			

Abbreviations: APX, apixaban; ASA, aspirin; CLP, clopidogrel; DBG-110MG, dabigatran 110mg; DBG-150MG, dabigatran 150mg; EDX-30MG, edoxaban 30mg; EDX-60MG, edoxaban 60mg; GNT, genotype-guided warfarin dosing; RVX, rivaroxaban; SM: self-management of warfarin; ST: self-testing of warfarin; WTH: Watchman device

eFigure 9.7: Network estimated risk ratios (95% confidence intervals) of intervention options on gastrointestinal bleeding

**DBG-110MG**									
4.75 (0.29,77.51)	**CONTROL**								
2.30 (0.18,29.06)	0.48 (0.03,9.22)	**ASA**							
0.91 (0.09,9.52)	0.19 (0.01,3.11)	0.40 (0.03,4.99)	**EDX-60MG**						
1.51 (0.13,17.65)	0.32 (0.02,5.67)	0.66 (0.05,9.17)	1.66 (0.14,19.33)	**UC+GNT**					
1.79 (0.19,17.10)	0.38 (0.02,5.66)	0.78 (0.16,3.83)	1.97 (0.21,18.72)	1.19 (0.11,12.68)	**APX**				
0.74 (0.14,3.89)	0.16 (0.01,2.53)	0.32 (0.03,4.06)	0.81 (0.08,8.50)	0.49 (0.04,5.73)	0.41 (0.04,3.95)	**DBG-150MG**			
0.81 (0.11,6.23)	0.17 (0.01,2.15)	0.35 (0.03,3.64)	0.89 (0.12,6.81)	0.54 (0.06,4.67)	0.45 (0.06,3.31)	1.10 (0.14,8.42)	**RVX**		
1.11 (0.21,5.87)	0.23 (0.02,2.20)	0.48 (0.07,3.28)	1.22 (0.23,6.41)	0.74 (0.12,4.51)	0.62 (0.13,2.86)	1.50 (0.29,7.93)	1.37 (0.42,4.43)	**UC**	
1.63 (0.16,17.17)	0.34 (0.02,5.60)	0.71 (0.06,8.99)	1.80 (0.34,9.46)	1.08 (0.09,12.68)	0.92 (0.10,8.74)	2.21 (0.21,23.23)	2.01 (0.26,15.42)	1.47 (0.28,7.76)	**EDX-30MG**

*** Treatments are ordered by SUCRA rank. Comparisons between treatments should be read from column to row for each outcome (row treatment is reference).**

Abbreviations: APX, apixaban; ASA, aspirin; CLP, clopidogrel; DBG-110MG, dabigatran 110mg; DBG-150MG, dabigatran 150mg; EDX-30MG, edoxaban 30mg; EDX-60MG, edoxaban 60mg; GNT, genotype-guided warfarin dosing; RVX, rivaroxaban; SM: self-management of warfarin; ST: self-testing of warfarin; WTH: Watchman device

eTable 9.8: Results of network meta-analysis of interventions options on myocardial infarction

The following table shows the effect sizes (risk ratio) and the rank order (SUCRA ranks) compared to usual warfarin care (UC).

Interventions	Risk ratio	95% Confidence interval	p-value	SUCRA rank
UC	reference			1
ASA	1.04	(0.68, 1.59)	0.848	2
RVX	0.82	(0.63, 1.06)	0.128	3
APX	0.88	(0.67, 1.15)	0.340	4
ASA+CLP	1.55	(0.92, 2.61)	0.100	5
DBG-110MG	1.36	(0.99, 1.88)	0.058	6
EDX-60MG	0.95	(0.75, 1.20)	0.686	7
DBG-150MG	1.40	(1.01, 1.92)	0.041	8
EDX-30MG	1.21	(0.97, 1.50)	0.096	9
UC+ST	1.49	(0.83, 2.70)	0.185	10
CONTROL	1.00	(0.14, 7.00)	0.996	11
**Number of RCTs**	**16**			

Abbreviations: APX, apixaban; ASA, aspirin; CLP, clopidogrel; DBG-110MG, dabigatran 110mg; DBG-150MG, dabigatran 150mg; EDX-30MG, edoxaban 30mg; EDX-60MG, edoxaban 60mg; GNT, genotype-guided warfarin dosing; RVX, rivaroxaban; SM: self-management of warfarin; ST: self-testing of warfarin; WTH: Watchman device

eFigure 9.8: Network estimated risk ratios (95% confidence intervals) of intervention options on myocardial infarction

**UC**										
0.96 (0.63,1.47)	**ASA**									
1.22 (0.94,1.58)	1.27 (0.77,2.09)	**RVX**								
1.14 (0.87,1.48)	1.19 (0.78,1.80)	0.93 (0.64,1.35)	**APX**							
0.65 (0.38,1.09)	0.67 (0.34,1.32)	**0.53 (0.30,0.95)**	0.57 (0.32,1.02)	**ASA+CLP**						
0.73 (0.53,1.01)	0.76 (0.45,1.30)	**0.60 (0.40,0.91)**	0.64 (0.42,0.98)	1.13 (0.62,2.09)	**DBG-110MG**					
1.05 (0.83,1.33)	1.09 (0.67,1.78)	0.86 (0.61,1.22)	0.92 (0.65,1.31)	1.63 (0.92,2.88)	1.43 (0.96,2.13)	**EDX-60MG**				
**0.72 (0.52,0.99)**	0.75 (0.44,1.27)	**0.59 (0.39,0.88)**	**0.63 (0.42,0.95)**	1.11 (0.60,2.04)	0.98 (0.73,1.31)	0.68 (0.46,1.01)	**DBG-150MG**			
0.83 (0.67,1.03)	0.86 (0.54,1.39)	**0.68 (0.48,0.95)**	0.73 (0.52,1.03)	1.28 (0.73,2.26)	1.13 (0.77,1.67)	**0.79 (0.63,0.99)**	1.16 (0.79,1.71)	**EDX-30MG**		
0.67 (0.37,1.21)	0.70 (0.34,1.45)	0.55 (0.29,1.05)	0.59 (0.31,1.13)	1.04 (0.47,2.28)	0.91 (0.47,1.79)	0.64 (0.34,1.21)	0.94 (0.48,1.83)	0.81 (0.43,1.52)	**UC+ST**	
1.00 (0.14,7.07)	1.05 (0.14,7.71)	0.82 (0.12,5.89)	0.88 (0.12,6.32)	1.56 (0.21,11.72)	1.37 (0.19,9.90)	0.96 (0.13,6.83)	1.40 (0.19,10.13)	1.21 (0.17,8.62)	1.50 (0.20,11.51)	**CONTROL**

*** Treatments are ordered by SUCRA rank. Comparisons between treatments should be read from column to row for each outcome (row treatment is reference).**

Abbreviations: APX, apixaban; ASA, aspirin; CLP, clopidogrel; DBG-110MG, dabigatran 110mg; DBG-150MG, dabigatran 150mg; EDX-30MG, edoxaban 30mg; EDX-60MG, edoxaban 60mg; GNT, genotype-guided warfarin dosing; RVX, rivaroxaban; SM: self-management of warfarin; ST: self-testing of warfarin; WTH: Watchman device

S10 Appendix: Treatment ranking and surface under the cumulative ranking curves (SUCRA)

eFigure 10.1: SUCRA ranking curve for stroke or systemic embolism

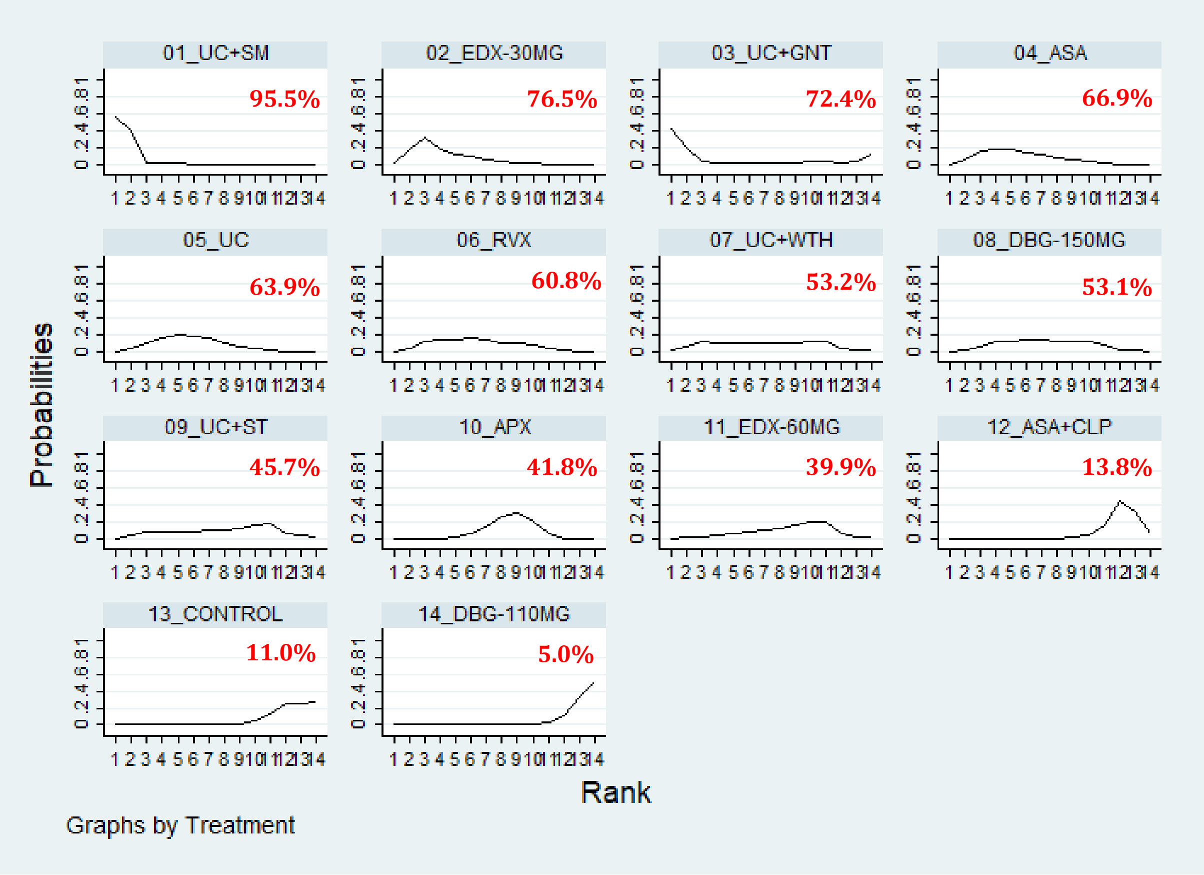

Abbreviations: APX, apixaban; ASA, aspirin; CLP, clopidogrel; DBG-110MG, dabigatran 110mg; DBG-150MG, dabigatran 150mg; EDX-30MG, edoxaban 30mg; EDX-60MG, edoxaban 60mg; GNT, genotype-guided warfarin dosing; RVX, rivaroxaban; SM: self-management of warfarin; ST: self-testing of warfarin; WTH: Watchman device

eFigure 10.2: SUCRA ranking curve for major bleeding

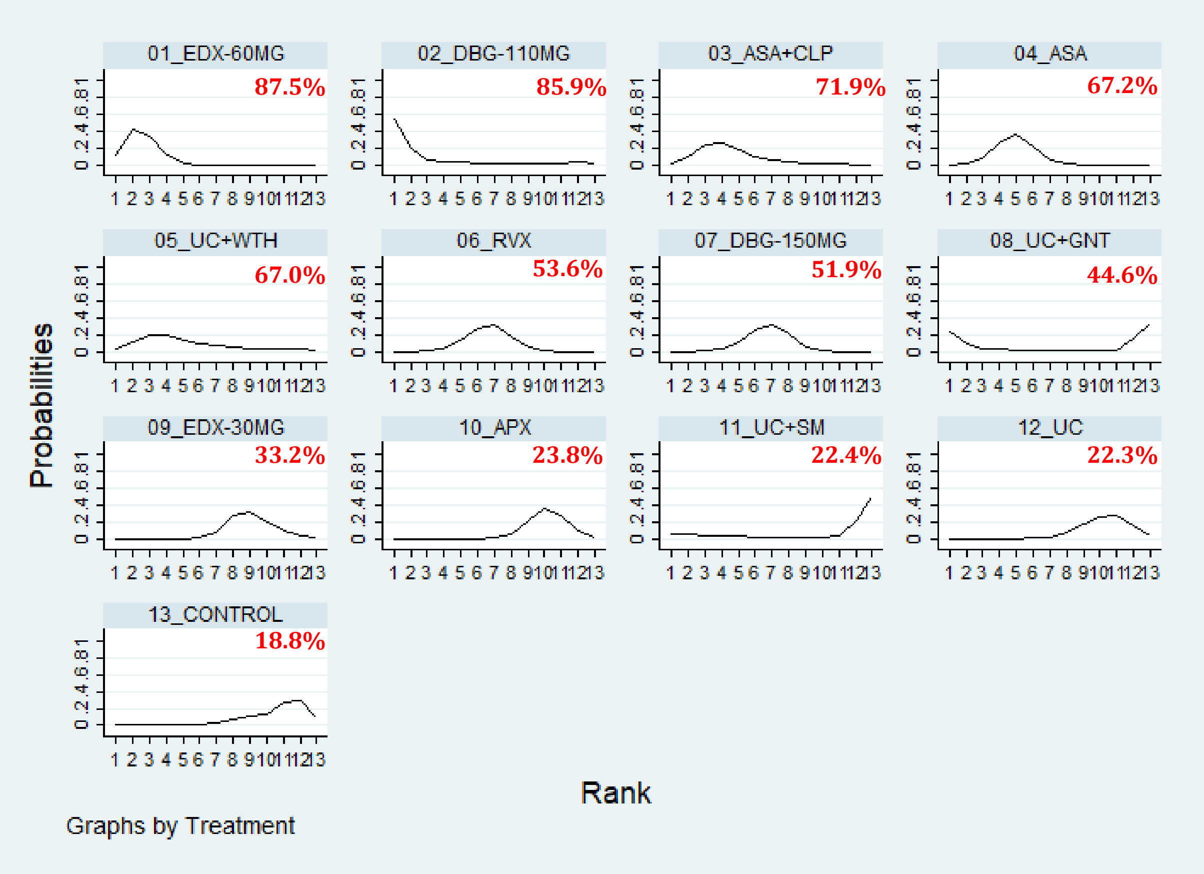

Abbreviations: APX, apixaban; ASA, aspirin; CLP, clopidogrel; DBG-110MG, dabigatran 110mg; DBG-150MG, dabigatran 150mg; EDX-30MG, edoxaban 30mg; EDX-60MG, edoxaban 60mg; GNT, genotype-guided warfarin dosing; RVX, rivaroxaban; SM: self-management of warfarin; ST: self-testing of warfarin; WTH: Watchman device

eFigure 10.3: SUCRA ranking curve for all-cause mortality

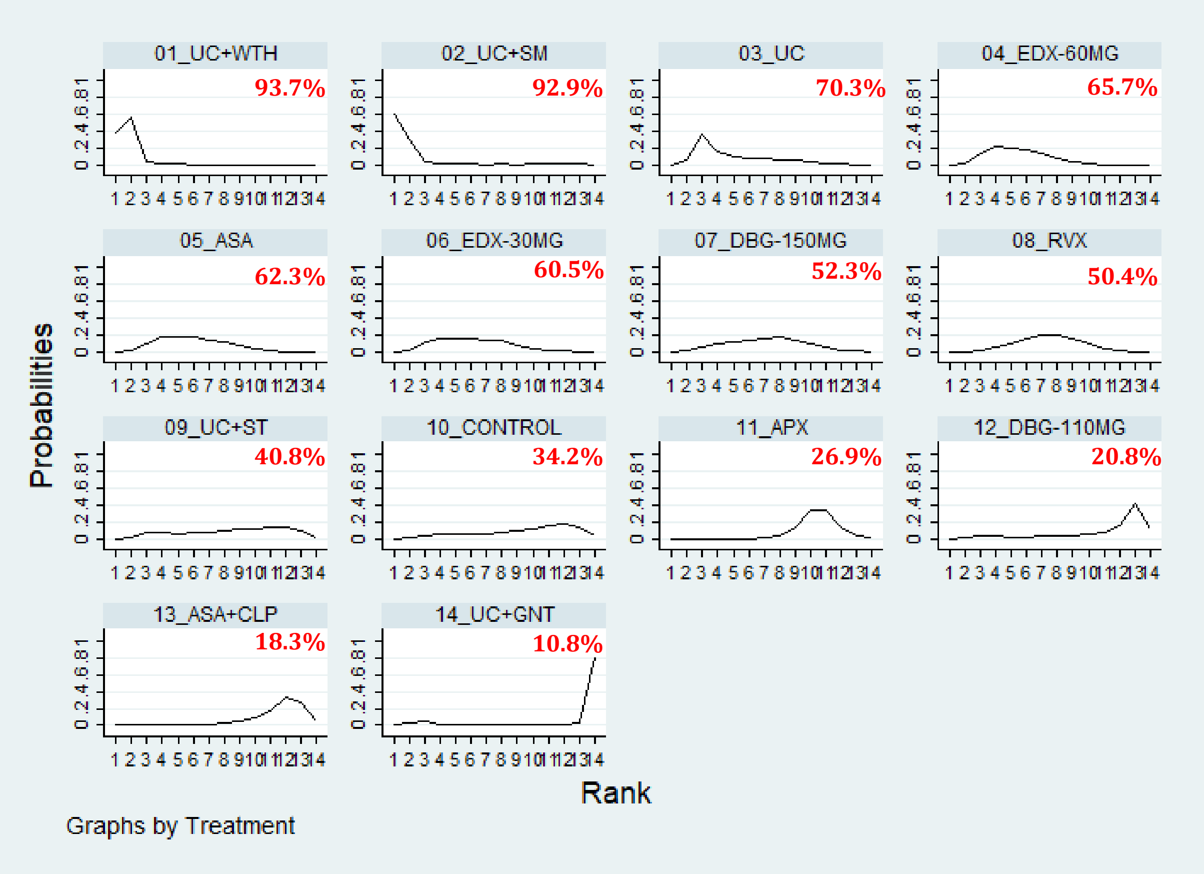

Abbreviations: APX, apixaban; ASA, aspirin; CLP, clopidogrel; DBG-110MG, dabigatran 110mg; DBG-150MG, dabigatran 150mg; EDX-30MG, edoxaban 30mg; EDX-60MG, edoxaban 60mg; GNT, genotype-guided warfarin dosing; RVX, rivaroxaban; SM: self-management of warfarin; ST: self-testing of warfarin; WTH: Watchman device

eFigure 10.4: SUCRA ranking curve for ischemic stroke

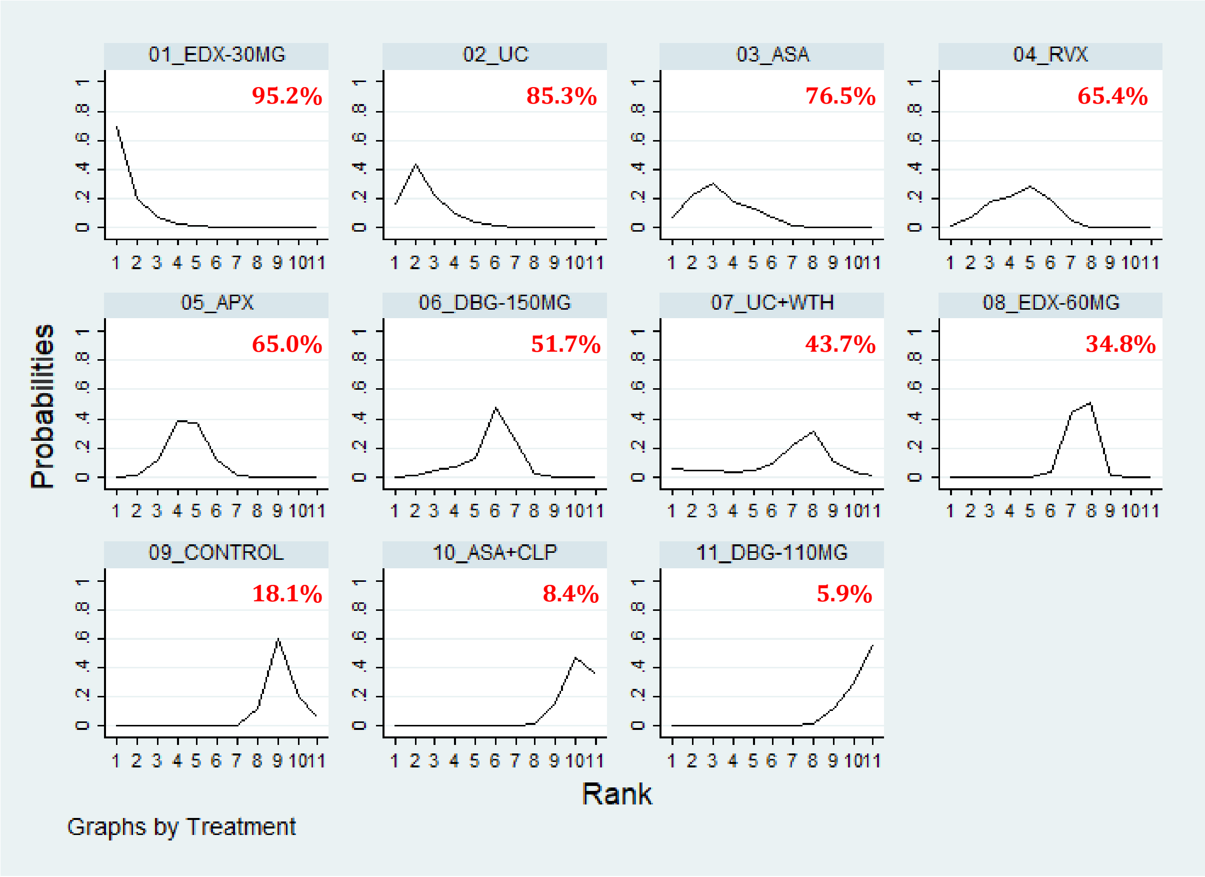

Abbreviations: APX, apixaban; ASA, aspirin; CLP, clopidogrel; DBG-110MG, dabigatran 110mg; DBG-150MG, dabigatran 150mg; EDX-30MG, edoxaban 30mg; EDX-60MG, edoxaban 60mg; GNT, genotype-guided warfarin dosing; RVX, rivaroxaban; SM: self-management of warfarin; ST: self-testing of warfarin; WTH: Watchman device

eFigure 10.5: SUCRA ranking curve for clinically relevant non-major bleeding

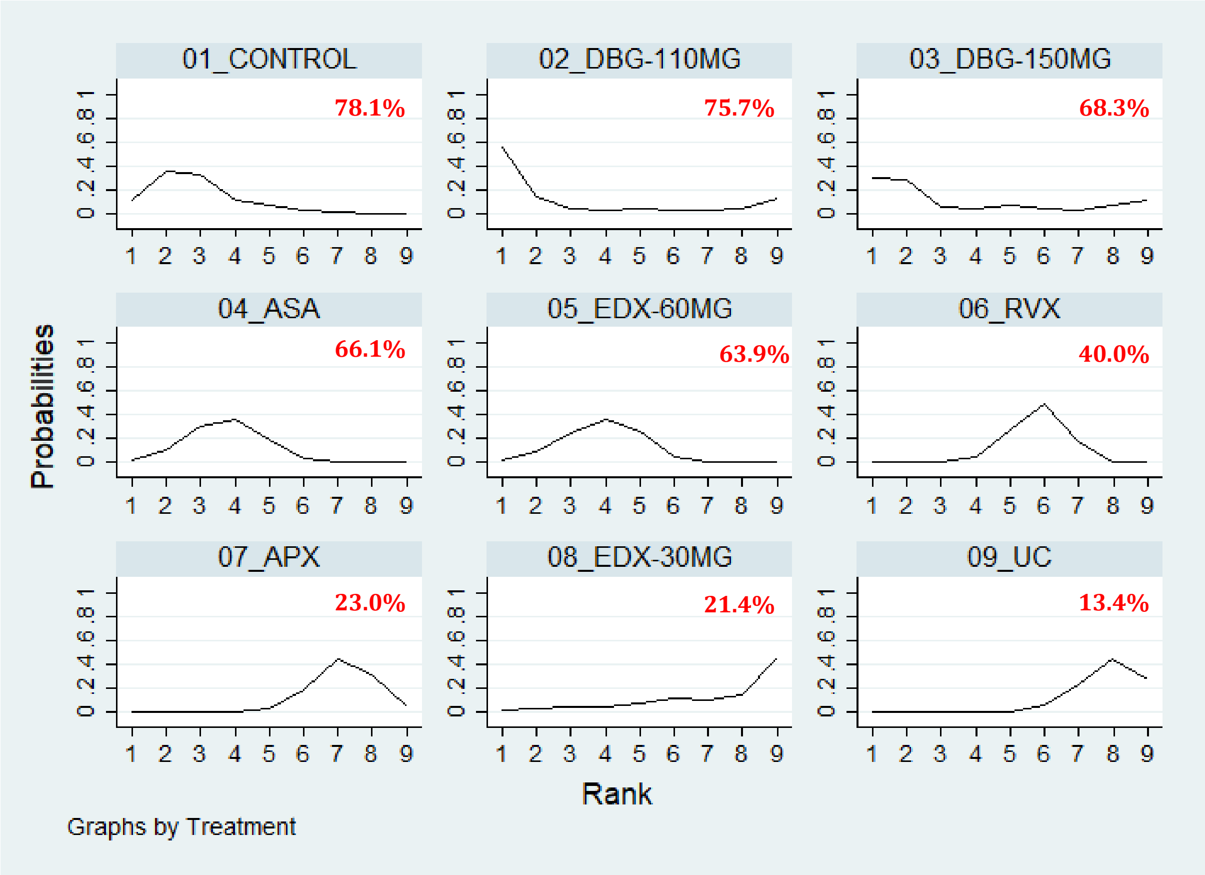

Abbreviations: APX, apixaban; ASA, aspirin; CLP, clopidogrel; DBG-110MG, dabigatran 110mg; DBG-150MG, dabigatran 150mg; EDX-30MG, edoxaban 30mg; EDX-60MG, edoxaban 60mg; GNT, genotype-guided warfarin dosing; RVX, rivaroxaban; SM: self-management of warfarin; ST: self-testing of warfarin; WTH: Watchman device

eFigure 10.6: SUCRA ranking curve for intracranial bleeding

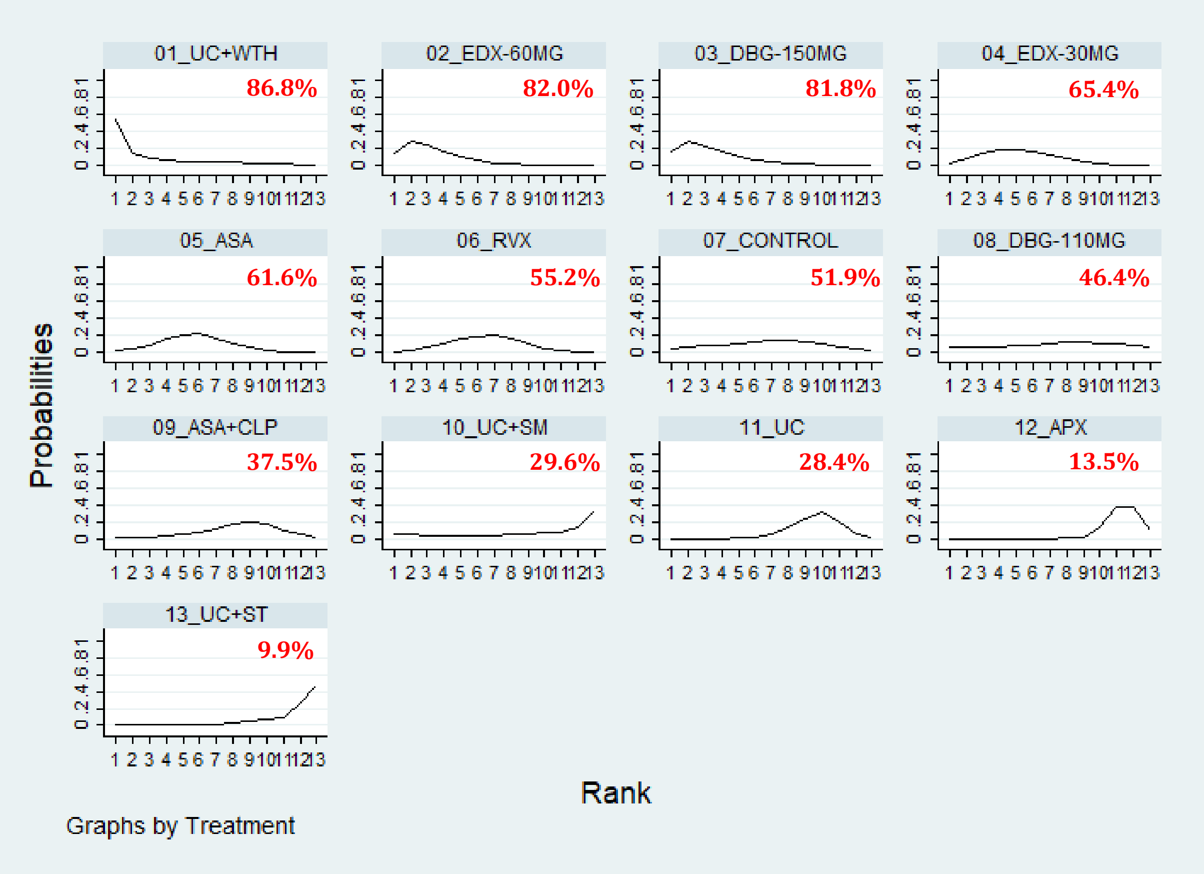

Abbreviations: APX, apixaban; ASA, aspirin; CLP, clopidogrel; DBG-110MG, dabigatran 110mg; DBG-150MG, dabigatran 150mg; EDX-30MG, edoxaban 30mg; EDX-60MG, edoxaban 60mg; GNT, genotype-guided warfarin dosing; RVX, rivaroxaban; SM: self-management of warfarin; ST: self-testing of warfarin; WTH: Watchman device

eFigure 10.7: SUCRA ranking curve for gastrointestinal bleeding

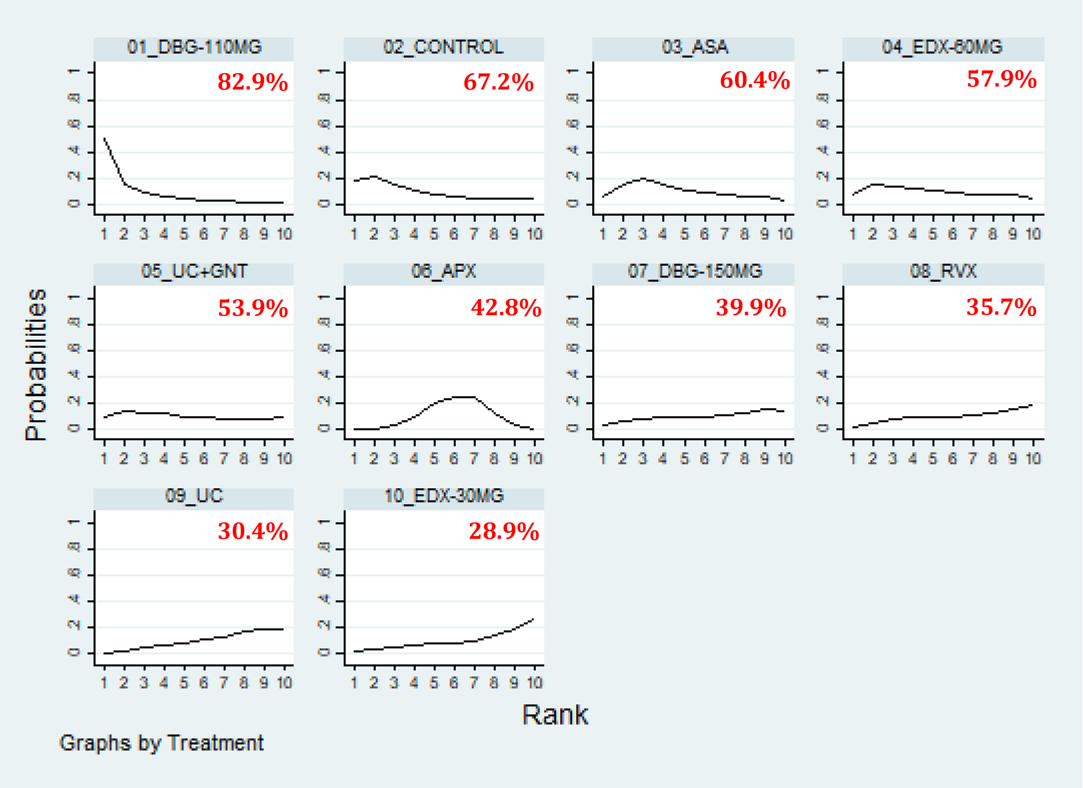

Abbreviations: APX, apixaban; ASA, aspirin; CLP, clopidogrel; DBG-110MG, dabigatran 110mg; DBG-150MG, dabigatran 150mg; EDX-30MG, edoxaban 30mg; EDX-60MG, edoxaban 60mg; GNT, genotype-guided warfarin dosing; RVX, rivaroxaban; SM: self-management of warfarin; ST: self-testing of warfarin; WTH: Watchman device

eFigure 10.8 SUCRA ranking curve for myocardial infarction

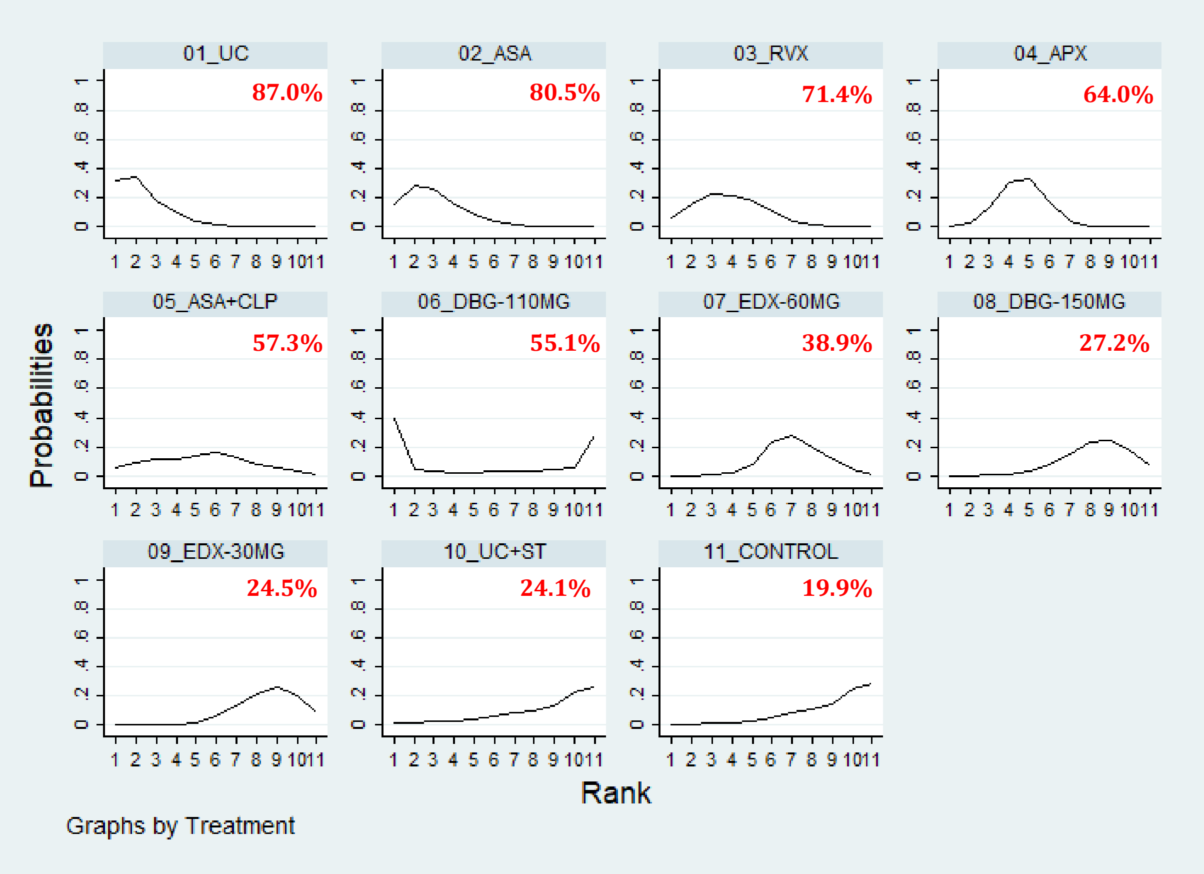

Abbreviations: APX, apixaban; ASA, aspirin; CLP, clopidogrel; DBG-110MG, dabigatran 110mg; DBG-150MG, dabigatran 150mg; EDX-30MG, edoxaban 30mg; EDX-60MG, edoxaban 60mg; GNT, genotype-guided warfarin dosing; RVX, rivaroxaban; SM: self-management of warfarin; ST: self-testing of warfarin; WTH: Watchman device

S11 Appendix: Cluster rank plot

eFigure 11.1: Cluster rank incorporating risk estimates of stroke or systemic embolism (efficacy) vs. major bleeding (safety)

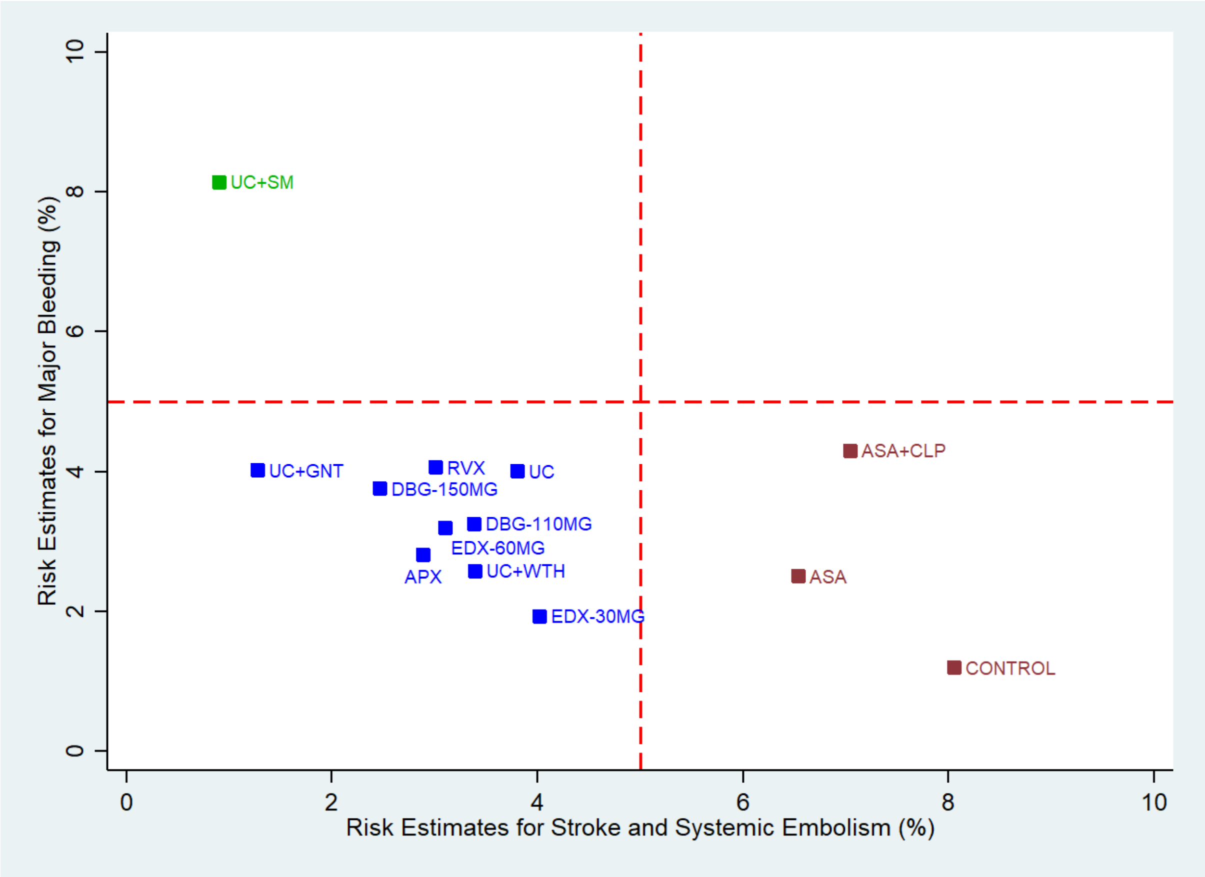

Abbreviations: APX, apixaban; ASA, aspirin; CLP, clopidogrel; DBG-110MG, dabigatran 110mg; DBG-150MG, dabigatran 150mg; EDX-30MG, edoxaban 30mg; EDX-60MG, edoxaban 60mg; GNT, genotype-guided warfarin dosing; RVX, rivaroxaban; SM: self-management of warfarin; ST: self-testing of warfarin; WTH: Watchman device

S12 Appendix: Sensitivity Analyses

eTable 12.1: Sensitivity analyses for the risk of stroke or systemic embolism with intervention options of different study characteristics

The following table shows the effect sizes (risk ratio) and the rank order (SUCRA ranks) compared to usual warfarin care (UC) before (main analysis) and after subgroup analyses.

Intervention	Standard analysis	SUCRA rank	Trials with AF participants only	SUCRA rank	Inclusion of studies from Year 2006 onwards*	SUCRA rank	Omitting small sample size studies (<25th percentile)	SUCRA rank	Omitting studies with high overall risk of bias	SUCRA rank
UC+SM	**0.24 (0.08, 0.68)**	1	0.33 (0.01, 8.37)	2	1.02 (0.02, 51.78)	8	**0.28 (0.11, 0.69)**	1	**0.23 (0.09, 0.62)**	1
EDX-30MG	1.06 (0.65, 1.72)	2	1.05 (0.64, 1.73)	1	1.06 (0.64, 1.75)	1	1.01 (0.55, 1.87)	3	1.14 (0.99, 1.31)	2
UC+GNT	0.33 (0.13, 8.45)	3	-	-	0.33 (0.01, 8.45)	2	0.33 (0.01, 8.67)	2	0.33 (0.01, 8.18)	3
ASA	**1.72 (1.29, 2.29)**	4	**1.72 (1.29, 2.30)**	3	**1.60 (1.13, 2.26)**	3	**1.65 (1.17, 2.33)**	4	**1.85 (1.48, 2.32)**	4
UC	Reference	5	Reference	4	Reference	4	Reference	5	Reference	5
RVX	0.79 (0.57, 1.10)	6	0.79 (0.57, 1.10)	5	0.79 (0.56, 1.12)	5	0.73 (0.45, 1.21)	6	**0.86 (0.75, 0.98)**	7
UC+WTH	0.89 (0.45, 1.78)	7	0.90 (0.45, 1.80)	6	0.89 (0.45, 1.79)	7	0.93 (0.42, 2.03)	8	0.84 (0.50, 1.44)	6
DBG-150MG	0.65 (0.39, 1.07)	8	0.65 (0.39, 1.08)	7	0.65 (0.39, 1.07)	6	0.66 (0.35, 1.25)	7	**0.66 (0.54, 0.82)**	8
UC+ST	0.99 (0.51, 1.93)	9	0.98 (0.02, 49.37)	8	0.99 (0.51, 1.95)	9	0.99 (0.45, 2.19)	9	0.99 (0.61, 1.62)	9
APX	0.76 (0.51, 1.12)	10	0.76 (0.51, 1.13)	9	0.73 (0.49, 1.11)	10	0.73 (0.44, 1.20)	11	**0.80 (0.68, 0.94)**	10
EDX-60MG	0.82 (0.51, 1.32)	11	0.81 (0.50, 1.33)	10	0.82 (0.50, 1.33)	11	0.79 (0.43, 1.45)	10	0.86 (0.74, 1.01)	11
ASA+CLP	**1.85 (1.07, 3.21)**	12	**1.85 (1.06, 3.24)**	11	**1.85 (1.07, 3.21)**	13	1.85 (0.93, 3.68)	12	**1.85 (1.37, 2.51)**	13
CONTROL	**2.12 (1.49, 3.01)**	13	**2.12 (1.49, 3.03)**	12	1.46 (0.72, 2.96)	14	**2.12 (1.43, 3.16)**	13	**1.88 (1.32, 2.67)**	12
DBG-110MG	0.89 (0.54, 1.46)	14	0.89 (0.54, 1.48)	13	0.89 (0.54, 1.46)	12	0.91 (0.48, 1.73)	14	0.91 (0.75, 1.11)	14
Overall inconsistency chi2 (P value)	4.23 (p-0.5169)		4.14 (p=0.5291)		0.04 (p=0.9799)		4.16 (p=0.5265)		0.80 (p=0.8483)	
Number of studies	36		32		27		30		32	

Abbreviations: APX, apixaban; ASA, aspirin; CLP, clopidogrel; DBG-110MG, dabigatran 110mg; DBG-150MG, dabigatran 150mg; EDX-30MG, edoxaban 30mg; EDX-60MG, edoxaban 60mg; GNT, genotype-guided warfarin dosing; RVX, rivaroxaban; SM: self-management of warfarin; ST: self-testing of warfarin; WTH: Watchman device

eTable 12.2: Sensitivity analyses for the risk of major bleeding with interventions options with different major bleeding definitions

The following table shows the effect sizes (risk ratio) and the rank order (SUCRA ranks) compared to usual warfarin care (UC) before (standard analysis) and after sensitivity analyses.

Interventions	Main analysis (BARC 3-5 based definition)	SUCRA rank	ISTH (major)-based definition	SUCRA rank
EDX-60MG	**0.80 (0.71, 0.90)**	1	**0.81 (0.71, 0.94)**	1
DBG-110MG	**0.81 (0.71, 0.94)**	2	**0.80 (0.71, 0.90)**	2
ASA+CLP	1.07 (0.81, 1.42)	3	1.07 (0.81, 1.42)	3
ASA	**0.63 (0.41, 0.96)**	4	0.66 (0.43, 1.02)	4
UC+WTH	0.64 (0.35, 1.18)	5	0.64 (0.35, 1.18)	5
RVX	1.01 (0.89, 1.16)	6	1.01 (0.89, 1.16)	6
DBG-150MG	0.94 (0.82, 1.08)	7	0.94 (0.82, 1.08)	7
UC+GNT	1.00 (0.02, 50.40)	8	1.00 (0.02, 50.40)	8
EDX-30MG	**0.48 (0.42, 0.56)**	9	**0.48 (0.42, 0.56)**	9
APX	**0.70 (0.61, 0.81)**	10	**0.71 (0.62, 0.81)**	10
UC+SM	2.03 (0.19, 21.83)	11	2.03 (0.19, 21.83)	11
UC	Reference	12	Reference	12
CONTROL	0.30 (0.06, 1.47)	13	0.20 (0.02, 1.66)	13
Overall inconsistency chi2 (P value)	0.90 (p=0.6382)		1.61 (p=0.4480)	
Number of studies	23		21	

Abbreviations: APX, apixaban; ASA, aspirin; CLP, clopidogrel; DBG-110MG, dabigatran 110mg; DBG-150MG, dabigatran 150mg; EDX-30MG, edoxaban 30mg; EDX-60MG, edoxaban 60mg; GNT, genotype-guided warfarin dosing; RVX, rivaroxaban; SM: self-management of warfarin; ST: self-testing of warfarin; WTH: Watchman device

eTable 12.3: Sensitivity analyses for the risk of major bleeding with intervention options of different study characteristics

The following table shows the effect sizes (risk ratio) and the rank order (SUCRA ranks) compared to usual warfarin care (UC) before (main analysis) and after subgroup analyses.

Interventions	Standard analysis	SUCRA rank	Trials with AF participants only	SUCRA rank	Inclusion of studies from Year 2006 onwards*	SUCRA rank	Omitting small sample size studies (<25th percentile)	SUCRA rank	Omitting studies with high overall risk of bias	SUCRA rank
EDX-60MG	**0.80 (0.71, 0.90)**	1	**0.80 (0.71, 0.90)**	**1**	**0.80 (0.71, 0.90)**	1	**0.80 (0.71, 0.90)**	1	**0.80 (0.71, 0.90)**	1
DBG-110MG	**0.81 (0.71, 0.94)**	2	**0.81 (0.71, 0.94)**	**2**	**0.81 (0.71, 0.94)**	12	**0.81 (0.71, 0.94)**	2	**0.81 (0.71, 0.94)**	2
ASA+CLP	1.07 (0.81, 1.42)	3	1.07 (0.81, 1.42)	3	1.07 (0.81, 1.42)	2	1.07 (0.81, 1.42)	3	1.07 (0.81, 1.42)	3
ASA	**0.63 (0.41, 0.96)**	4	**0.63 (0.41, 0.96)**	**4**	**0.63 (0.41, 0.96)**	4	**0.59 (0.38, 0.92)**	4	**0.60 (0.39, 0.91)**	4
UC+WTH	0.64 (0.35, 1.18)	5	0.64 (0.35, 1.18)	5	0.64 (0.35, 1.18)	3	0.64 (0.35, 1.18)	5	0.64 (0.35, 1.18)	5
RVX	1.01 (0.89, 1.16)	6	1.01 (0.89, 1.16)	6	1.01 (0.89, 1.16)	5	1.02 (0.89, 1.16)	6	1.01 (0.89, 1.16)	6
DBG-150MG	0.94 (0.82, 1.08)	7	0.94 (0.82, 1.08)	7	0.94 (0.82, 1.08)	6	0.94 (0.82, 1.08)	7	0.94 (0.82, 1.08)	7
UC+GNT	1.00 (0.02, 50.40)	8	-	-	1.00 (0.02, 50.40)	7	1.00 (0.02, 50.40)	8	1.00 (0.02, 50.40)	8
EDX-30MG	**0.48 (0.42, 0.56)**	9	**0.48 (0.42, 0.56)**	**8**	**0.48 (0.42, 0.56)**	8	**0.48 (0.42, 0.56)**	9	**0.48 (0.42, 0.56)**	9
APX	**0.70 (0.61, 0.81)**	10	**0.70 (0.61, 0.81)**	**9**	**0.70 (0.61, 0.81)**	9	**0.70 (0.61, 0.80)**	10	**0.70 (0.61, 0.80)**	10
UC+SM	2.03 (0.19, 21.83)	11	-	-	2.03 (0.19, 21.83)	11	-	-	2.03 (0.19, 21.83)	11
UC	Reference	12	Reference	10	Reference	10	Reference	11	Reference	12
CONTROL	0.30 (0.06, 1.47)	13	0.30 (0.06, 1.47)	11	-	-	0.30 (0.06, 1.47)	12	0.30 (0.01, 1.47)	13
UC+ST	-	-	0.90 (p=0.6382)		0.90 (p=0.6382)		-	-	-	-
Overall inconsistency chi2 (P value)	0.90 (p=0.6382)		21		21		2.34 (p=0.3111)		1.36 (p=0.5066)	
Number of studies	23						18		22	

Abbreviations: APX, apixaban; ASA, aspirin; CLP, clopidogrel; DBG-110MG, dabigatran 110mg; DBG-150MG, dabigatran 150mg; EDX-30MG, edoxaban 30mg; EDX-60MG, edoxaban 60mg; GNT, genotype-guided warfarin dosing; RVX, rivaroxaban; SM: self-management of warfarin; ST: self-testing of warfarin; WTH: Watchman device

eTable 12.4: Sensitivity analyses for the risk of all-cause mortality with intervention options of different study characteristics

The following table shows the effect sizes (risk ratio) and the rank order (SUCRA ranks) compared to usual warfarin care (UC) before (main analysis) and after subgroup analyses.

Interventions	Standard analysis	SUCRA rank	Trials with AF participants only	SUCRA rank	Inclusion of studies from Year 2006 onwards*	SUCRA rank	Omitting small sample size studies (<25th percentile)	SUCRA rank	Omitting studies with high overall risk of bias	SUCRA rank
UC+WTH	0.49 (0.28, 0.86)	**1**	0.49 (0.28, 0.86)	1	0.49 (0.28, 0.86)	1	0.49 (0.81, 0.86)	2	0.49 (0.28, 0.86)	1
UC+SM	0.42 (0.17, 1.04)	**2**	-	-	1.02 (0.02, 50.41)	9	0.40 (0.16, 1.02)	1	0.42 (0.17, 1.04)	2
UC	Reference	**3**	Reference	2	Reference	2	Reference	3	Reference	3
EDX-60MG	0.92 (0.84, 1.01)	**4**	0.92 (0.84, 1.01)	3	0.88 (0.84, 1.01)	3	0.92 (0.84, 1.01)	4	0.92 (0.84, 1.01)	4
ASA	1.07 (0.92, 1.24)	**5**	1.07 (0.92, 1.24)	4	1.08 (0.91, 1.28)	4	1.07 (0.92, 1.24)	5	1.06 (0.92, 1.23)	5
EDX-30MG	0.88 (0.80, 0.96)	**6**	0.88 (0.80, 0.96)	5	0.89 (0.80, 0.96)	5	0.88 (0.80, 0.96)	6	0.88 (0.80, 0.96)	6
DBG-150MG	0.89 (0.79, 1.01)	**7**	0.89 (0.79, 1.01)	6	0.92 (0.79, 1.01)	6	0.89 (0.79, 1.01)	7	0.89 (0.79, 1.01)	7
RVX	0.85 (0.71, 1.01)	**8**	0.85 (0.71, 1.01)	7	0.92 (0.71, 1.01)	7	0.85 (0.71, 1.01)	8	0.85 (0.71, 1.01)	8
UC+ST	0.96 (0.78, 1.19)	**9**	-	-	0.96 (0.78, 1.19)	10	0.96 (0.78, 1.19)	9	0.96 (0.78, 1.19)	9
CONTROL	1.11 (0.80, 1.56)	**10**	1.11 (0.80, 1.56)	8	0.93 (0.37, 2.30)	11	1.11 (0.80, 1.56)	10	1.07 (0.54, 2.12)	11
APX	0.89 (0.80, 0.98)	**11**	0.89 (0.80, 0.98)	9	0.89 (0.80, 0.98)	12	0.89 (0.80, 0.98)	11	0.89 (0.80, 0.98)	12
DBG-110MG	0.92 (0.81, 1.04)	**12**	0.92 (0.81, 1.04)	10	0.92 (0.81, 1.04)	8	0.92 (0.81, 1.04)	12	0.92 (0.81, 1.04)	10
ASA+CLP	1.00 (0.80, 1.23)	**13**	1.00 (0.80, 1.23)	11	1.00 (0.80, 1.23)	13	1.00 (0.80, 1.23)	13	1.00 (0.80, 1.23)	13
UC+GNT	2.51 (0.49, 12.81)	**14**	-	-	2.51 (0.49, 12.81)	14	2.51 (0.49, 12.81)	14	2.51 (0.49, 12.81)	14
Overall inconsistency chi2 (P value)	0.60 (p=0.7407)		0.60 (p=0.7407)		0.29 (p=0.5930)		0.63 (p=0.7303)		0.70 (p=0.7061)	
Number of studies	26		22		21		20		24	

Abbreviations: APX, apixaban; ASA, aspirin; CLP, clopidogrel; DBG-110MG, dabigatran 110mg; DBG-150MG, dabigatran 150mg; EDX-30MG, edoxaban 30mg; EDX-60MG, edoxaban 60mg; GNT, genotype-guided warfarin dosing; RVX, rivaroxaban; SM: self-management of warfarin; ST: self-testing of warfarin; WTH: Watchman device

S13 Appendix: Adjusted funnel plots

eFigure 13.1: Comparison-adjusted funnel plot for the network of stroke or systemic embolism in all comparisons

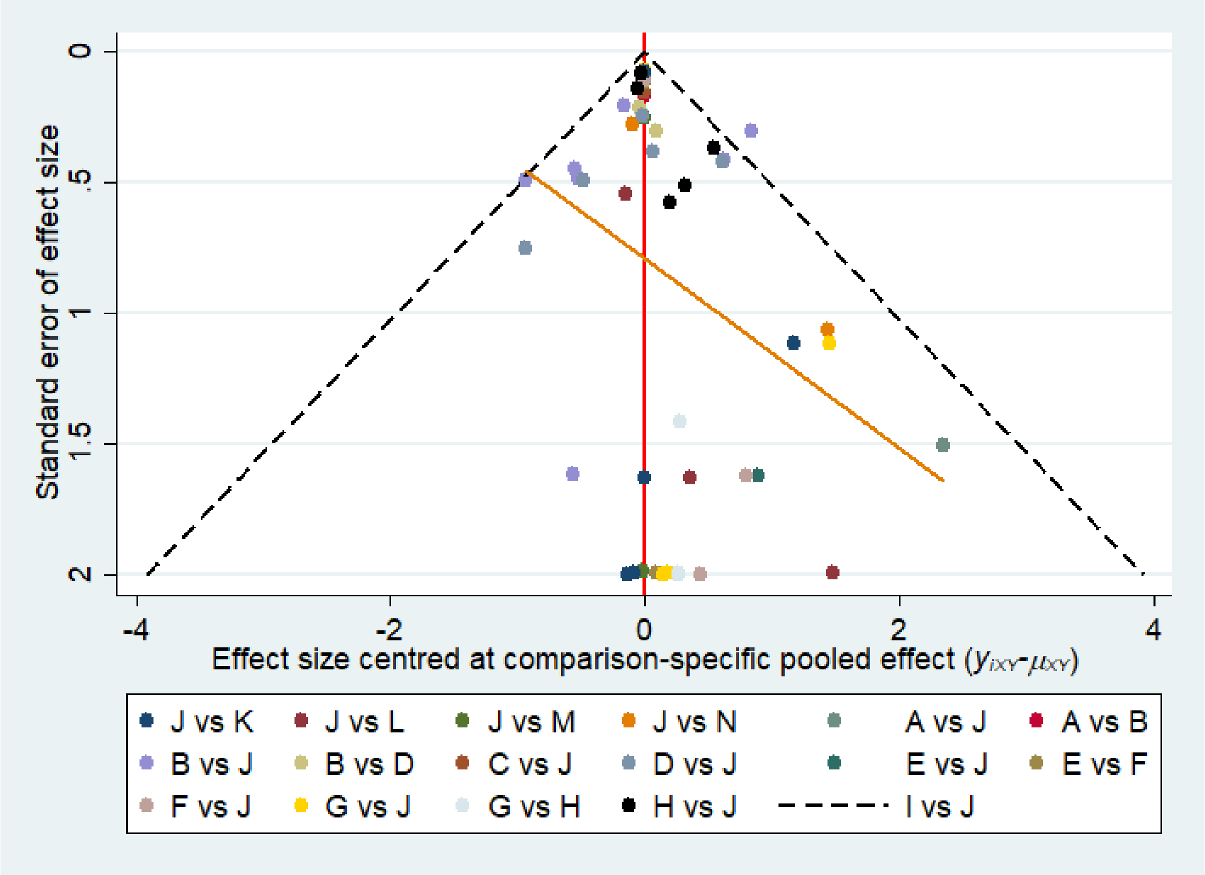

Abbreviations: APX, apixaban; ASA, aspirin; CLP, clopidogrel; DBG-110MG, dabigatran 110mg; DBG-150MG, dabigatran 150mg; EDX-30MG, edoxaban 30mg; EDX-60MG, edoxaban 60mg; GNT, genotype-guided warfarin dosing; RVX, rivaroxaban; SM: self-management of warfarin; ST: self-testing of warfarin; WTH: Watchman device

eFigure 13.2: Comparison-adjusted funnel plot for the network of major bleeding in all comparisons

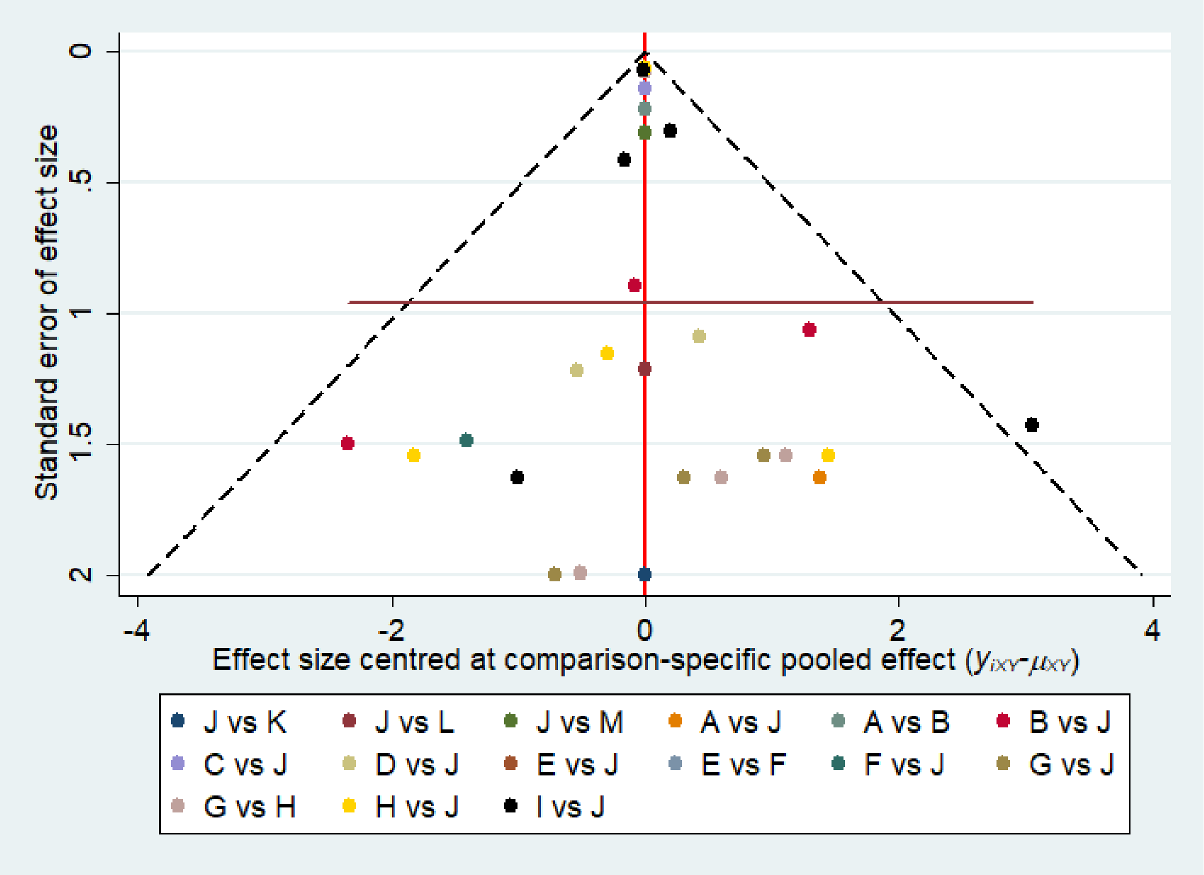

Abbreviations: APX, apixaban; ASA, aspirin; CLP, clopidogrel; DBG-110MG, dabigatran 110mg; DBG-150MG, dabigatran 150mg; EDX-30MG, edoxaban 30mg; EDX-60MG, edoxaban 60mg; GNT, genotype-guided warfarin dosing; RVX, rivaroxaban; SM: self-management of warfarin; ST: self-testing of warfarin; WTH: Watchman device

eFigure 13.3: Comparison-adjusted funnel plot for the network of all-cause mortality in all comparisons

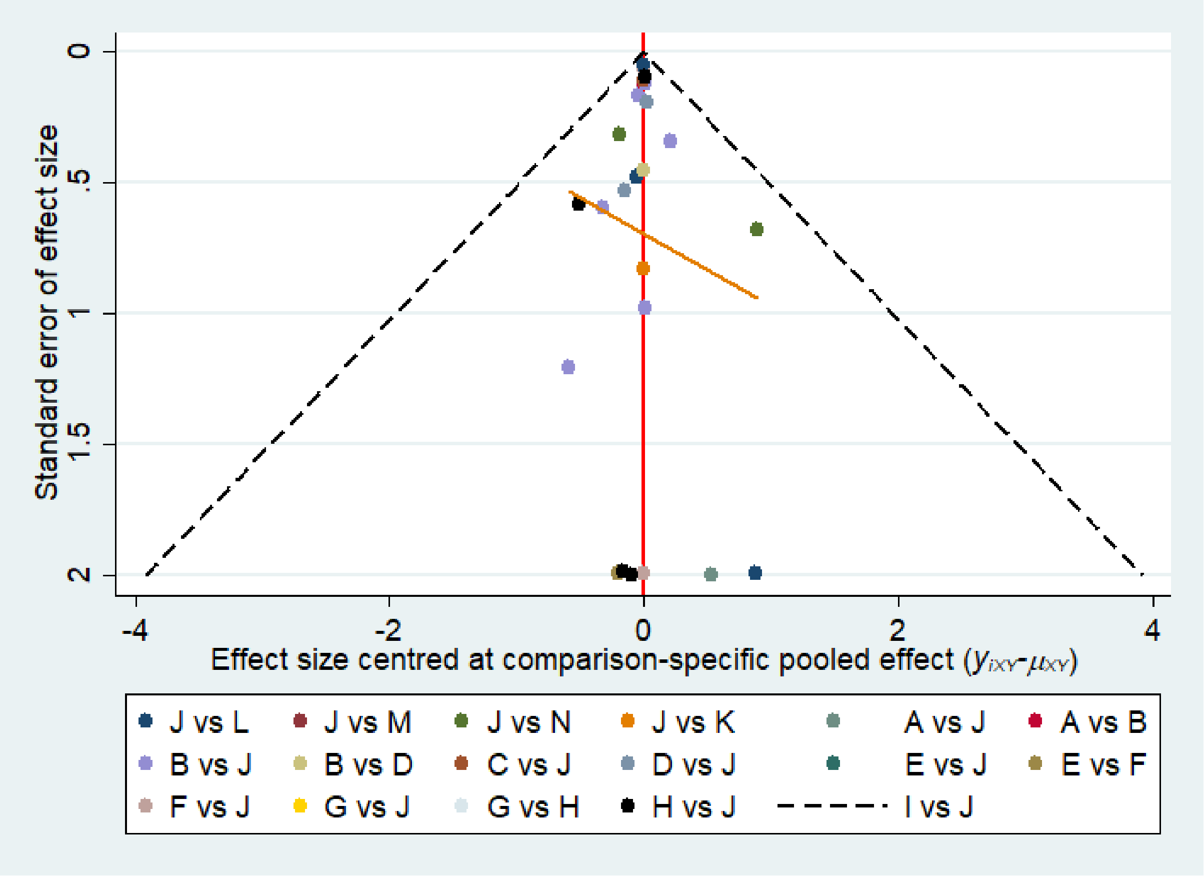

Abbreviations: APX, apixaban; ASA, aspirin; CLP, clopidogrel; DBG-110MG, dabigatran 110mg; DBG-150MG, dabigatran 150mg; EDX-30MG, edoxaban 30mg; EDX-60MG, edoxaban 60mg; GNT, genotype-guided warfarin dosing; RVX, rivaroxaban; SM: self-management of warfarin; ST: self-testing of warfarin; WTH: Watchman device

eFigure 13.4: Comparison-adjusted funnel plot for the network of ischemic stroke in all comparisons

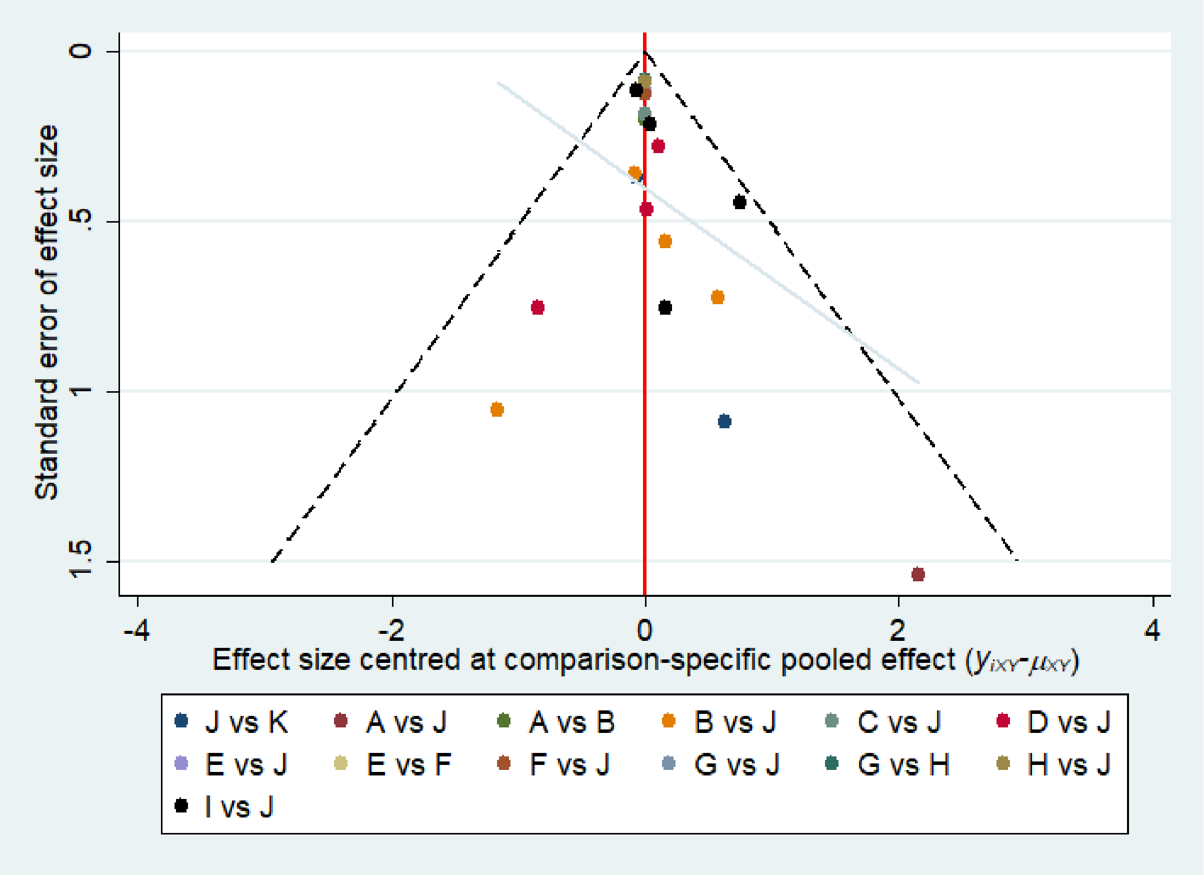

Abbreviations: APX, apixaban; ASA, aspirin; CLP, clopidogrel; DBG-110MG, dabigatran 110mg; DBG-150MG, dabigatran 150mg; EDX-30MG, edoxaban 30mg; EDX-60MG, edoxaban 60mg; GNT, genotype-guided warfarin dosing; RVX, rivaroxaban; SM: self-management of warfarin; ST: self-testing of warfarin; WTH: Watchman device

eFigure 13.5: Comparison-adjusted funnel plot for the network of clinically relevant non-major bleeding in all comparisons

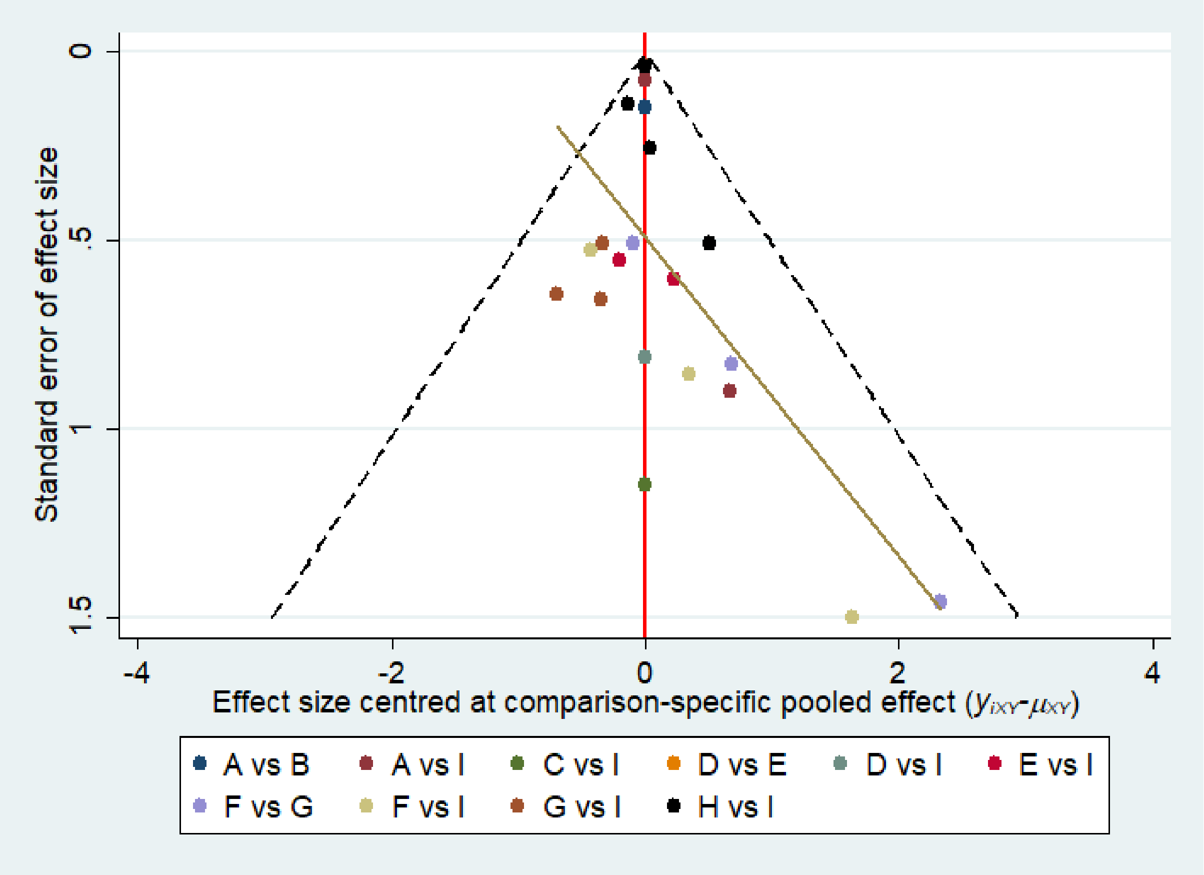

Abbreviations: APX, apixaban; ASA, aspirin; CLP, clopidogrel; DBG-110MG, dabigatran 110mg; DBG-150MG, dabigatran 150mg; EDX-30MG, edoxaban 30mg; EDX-60MG, edoxaban 60mg; GNT, genotype-guided warfarin dosing; RVX, rivaroxaban; SM: self-management of warfarin; ST: self-testing of warfarin; WTH: Watchman device

eFigure 13.6: Comparison-adjusted funnel plot for the network of intracranial bleeding in all comparisons

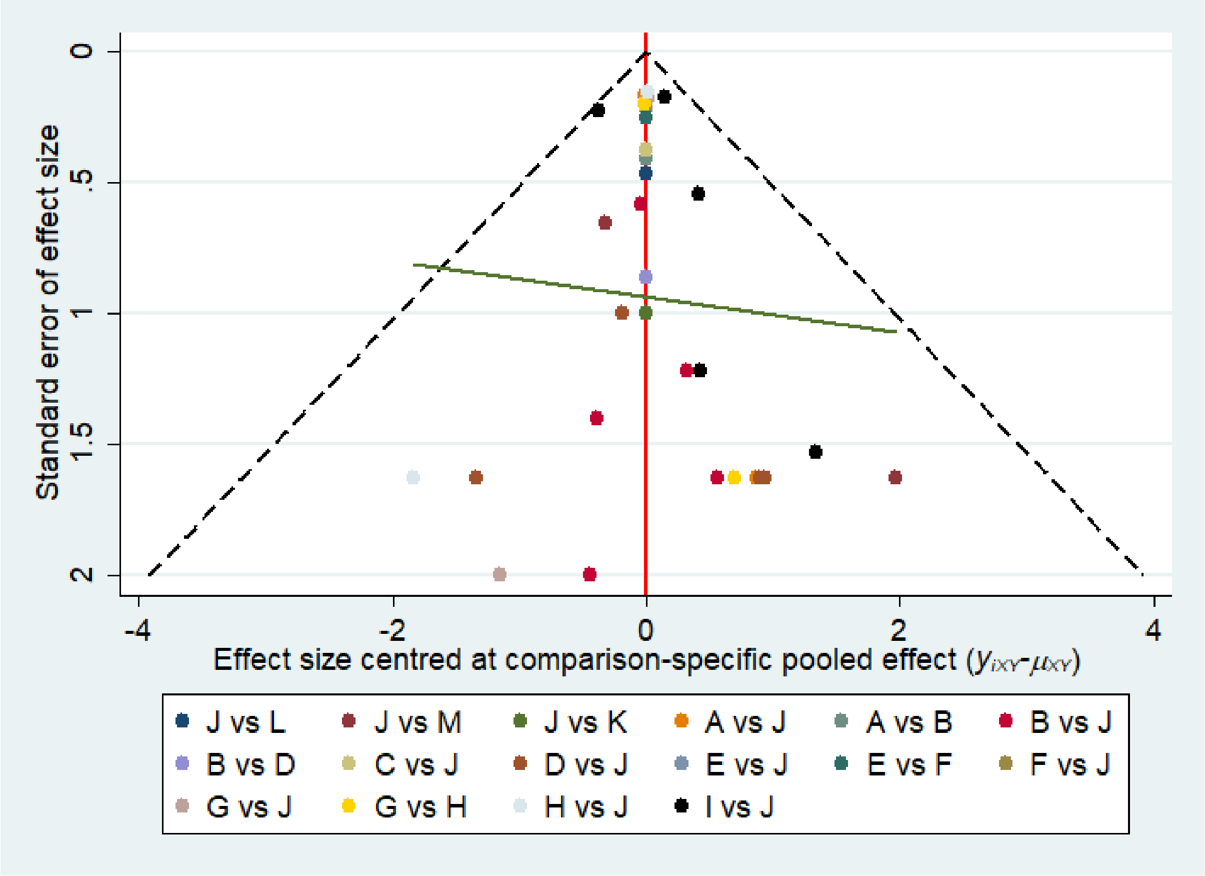

Abbreviations: APX, apixaban; ASA, aspirin; CLP, clopidogrel; DBG-110MG, dabigatran 110mg; DBG-150MG, dabigatran 150mg; EDX-30MG, edoxaban 30mg; EDX-60MG, edoxaban 60mg; GNT, genotype-guided warfarin dosing; RVX, rivaroxaban; SM: self-management of warfarin; ST: self-testing of warfarin; WTH: Watchman device

eFigure 13.7: Comparison-adjusted funnel plot for the network of gastrointestinal bleeding in all comparisons

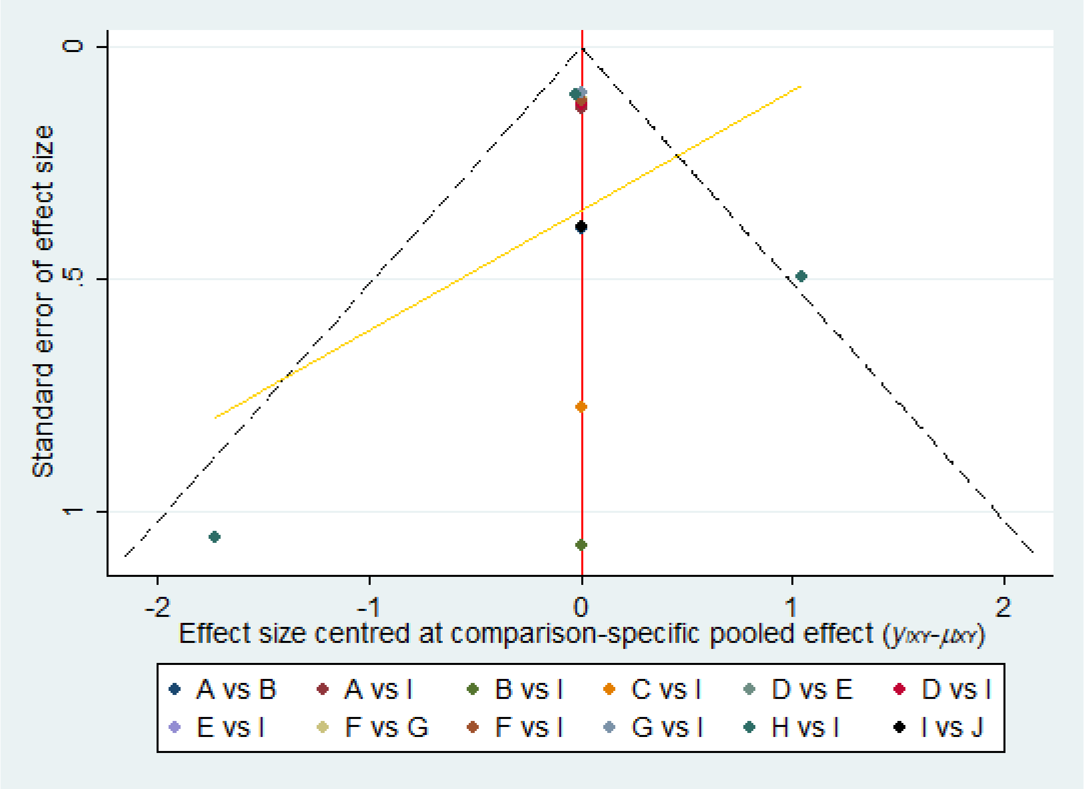

Abbreviations: APX, apixaban; ASA, aspirin; CLP, clopidogrel; DBG-110MG, dabigatran 110mg; DBG-150MG, dabigatran 150mg; EDX-30MG, edoxaban 30mg; EDX-60MG, edoxaban 60mg; GNT, genotype-guided warfarin dosing; RVX, rivaroxaban; SM: self-management of warfarin; ST: self-testing of warfarin; WTH: Watchman device

eFigure 13.8: Comparison-adjusted funnel plot for the network of myocardial infarction in all comparisons

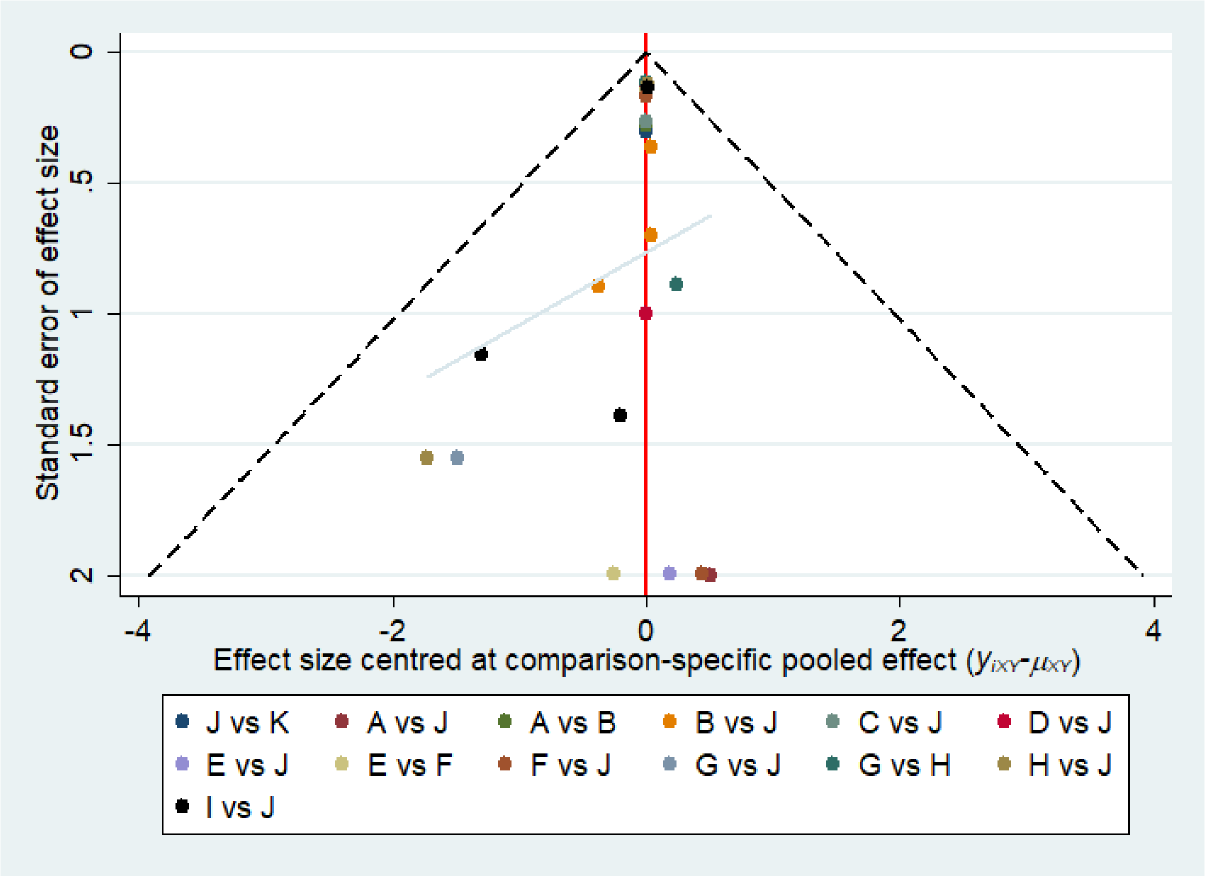

Abbreviations: APX, apixaban; ASA, aspirin; CLP, clopidogrel; DBG-110MG, dabigatran 110mg; DBG-150MG, dabigatran 150mg; EDX-30MG, edoxaban 30mg; EDX-60MG, edoxaban 60mg; GNT, genotype-guided warfarin dosing; RVX, rivaroxaban; SM: self-management of warfarin; ST: self-testing of warfarin; WTH: Watchman device

S14 Appendix: Assessment of small study effects by Egger's test in each outcome

eTable14.1: Assessment of small study effects by Egger's test for each pairwise in each outcome

Outcome		Comparison			Number of studies	P-value
**Stroke or Systemic Embolism**	B - A	ASA	vs.	APX	1	
	D - B	CONTROL	vs.	ASA	2	
	F - E	DBG-150MG	vs.	DBG-110MG	1	
	H - G	EDX-60MG	vs.	EDX-30MG	2	
	J - A	UC	vs.	APX	2	
	J - B	UC	vs.	ASA	7	0.639
	J - C	UC	vs.	ASA+CLP	1	
	J - D	UC	vs.	CONTROL	5	0.495
	J - E	UC	vs.	DBG-110MG	2	
	J - F	UC	vs.	DBG-150MG	2	
	J - G	UC	vs.	EDX-30MG	2	
	J - H	UC	vs.	EDX-60MG	2	
	J - I	UC	vs.	RVX	5	0.129
	K - J	UC+GNT	vs.	UC	1	
	L - J	UC+SM	vs.	UC	2	
	M - J	UC+ST	vs.	UC	1	
	N - J	UC+WTH	vs.	UC	2	
**All-cause Mortality**	B - A	ASA	vs.	APX	1	
	D - B	CONTROL	vs.	ASA	1	
	F - E	DBG-150MG	vs.	DBG-110MG	1	
	J - B	UC	vs.	ASA	6	
	H - G	EDX-60MG	vs.	EDX-30MG	1	
	J - A	UC	vs.	APX	1	
	J - B	UC	vs.	ASA	6	0.506
	J - C	UC	vs.	ASA+CLP	1	
	J - D	UC	vs.	CONTROL	2	
	J - E	UC	vs.	DBG-110MG	1	
	J - F	UC	vs.	DBG-150MG	1	
	J - G	UC	vs.	EDX-30MG	1	
	J - H	UC	vs.	EDX-60MG	1	
	J - I	UC	vs.	RVX	2	
	K - J	UC+GNT	vs.	UC	1	
	M - J	UC+ST	vs.	UC	1	
	N - J	UC+WTH	vs.	UC	2	
**Major Bleeding**	B - A	ASA	vs.	APX	1	
	F - E	DBG-150MG	vs.	DBG-110MG	1	
	H - G	EDX-60MG	vs.	EDX-30MG	3	0.198
	J - A	UC	vs.	APX	2	

	J - B	UC	vs.	ASA	3	0.570
	J - C	UC	vs.	ASA+CLP	1	
	J - D	UC	vs.	CONTROL	2	
	J - E	UC	vs.	DBG-110MG	1	
	J - F	UC	vs.	DBG-150MG	2	
	J - G	UC	vs.	EDX-30MG	3	0.307
	J - H	UC	vs.	EDX-60MG	4	0.831
	J - I	UC	vs.	RVX	5	0.503
	K - J	UC+GNT	vs.	UC	N/A	
	M - J	UC+WTH	vs.	UC	1	
**Clinically Relevant Non-Major Bleeding (CRNMB)**	B - A	ASA	vs.	APX	1	
	I - A	UC	vs.	APX	2	
	I - C	UC	vs.	CONTROL	1	0.264
	E - D	DBG-150MG	vs.	DBG-110MG	1	
	I - D	UC	vs.	DBG-110MG	1	
	I - E	UC	vs.	DBG-150MG	2	
	G - F	EDX-60MG	vs.	EDX-30MG	4	
	I - F	UC	vs.	EDX-30MG	4	0.702
	I - G	UC	vs.	EDX-60MG	4	**0.049**
	I - H	UC	vs.	RVX	4	0.831
**Ischemic Stroke**	B - A	ASA	vs.	APX	1	
	F - E	DBG-150MG	vs.	DBG-110MG	1	
	H - G	EDX-60MG	vs.	EDX-30MG	1	
	J - A	UC	vs.	APX	2	
	J - B	UC	vs.	ASA	4	0.790
	J - C	UC	vs.	ASA+CLP	1	
	J - D	UC	vs.	CONTROL	3	0.264
	J - E	UC	vs.	DBG-110MG	1	
	J - F	UC	vs.	DBG-150MG	1	
	J - G	UC	vs.	EDX-30MG	1	
	J - H	UC	vs.	EDX-60MG	1	
	J - I	UC	vs.	RVX	4	0.247
	K - J	UC+WTH	vs.	UC	2	
**Intracranial Bleeding**	B - A	ASA	vs.	APX	1	
	J - A	UC	vs.	APX	2	
	D - B	CONTROL	vs.	ASA	1	
	J - B	UC	vs.	ASA	4	
	J - C	UC	vs.	ASA+CLP	1	
	J - D	UC	vs.	CONTROL	4	0.616
	F - E	DBG-150MG	vs.	DBG-110MG	1	
	J - E	UC	vs.	DBG-110MG	1	0.782
	J - F	UC	vs.	DBG-150MG	1	
	H - G	EDX-60MG	vs.	EDX-30MG	2	
	J - G	UC	vs.	EDX-30MG	1	
	J - H	UC	vs.	EDX-60MG	2	
	J - I	UC	vs.	RVX	5	0.504

	K - J	UC+SM	vs.	UC	1	
	L - J	UC+ST	vs.	UC	1	
	M - J	UC+WTH	vs.	UC	2	
**Gastrointestinal Bleeding**	B - A	ASA	vs.	APX	1	
	I - A	UC	vs.	APX	1	
	I - B	UC	vs.	ASA	1	
	I - C	UC	vs.	CONTROL	1	
	E - D	DBG-150MG	vs.	DBG-110MG	1	
	I - D	UC	vs.	DBG-110MG	1	
	I - E	UC	vs.	DBG-150MG	1	
	G - F	EDX-60MG	vs.	EDX-30MG	1	
	I - F	UC	vs.	EDX-30MG	1	
	I - G	UC	vs.	EDX-60MG	1	
	I - H	UC	vs.	RVX	3	0.962
	J - I	UC+GNT	vs.	UC	1	0.927
**Myocardial Infarction**	B - A	ASA	vs.	APX	1	
	J - A	UC	vs.	APX	1	
	J - B	UC	vs.	ASA	3	
	J - C	UC	vs.	ASA+CLP	1	
	J - D	UC	vs.	CONTROL	1	0.463
	F - E	DBG-150MG	vs.	DBG-110MG	1	
	J - E	UC	vs.	DBG-110MG	1	
	J - F	UC	vs.	DBG-150MG	1	
	H - G	EDX-60MG	vs.	EDX-30MG	2	
	J - G	UC	vs.	EDX-30MG	2	
	J - H	UC	vs.	EDX-60MG	2	
	J - I	UC	vs.	RVX	3	0.424
	K - J	UC+ST	vs.	UC	1	

S15 Appendix: Evaluation of the quality of evidence using GRADE framework for all outcomes

eTable15.1: Estimates of effects and quality ratings for comparison of treatment options in all of the outcomes.

Comparison	Direct evidence		Indirect evidence		Network meta-analysis	
	Risk ratio (95% confidence interval)	Quality of evidence	Risk ratio (95% confidence interval)	Quality of evidence	Risk ratio (95% confidence interval)	Quality of evidence
**Stroke or systemic embolism**						
ASA vs. APX	2.22 (1.61,3.13)	⊕⊕⊕⊕ HIGH	2.39 (1.19,4.79)	⊕⊕◯◯ LOW [§]	2.27 (1.51,3.42)	⊕⊕⊕⊕ HIGH
ASA+CLP vs. APX			2.44 (1.24,4.80)	⊕⊕◯◯ LOW [++]	2.44 (1.24,4.80)	⊕⊕◯◯ LOW
CONTROL vs. APX			2.80 (1.68,4.64)	⊕◯◯◯ VERY LOW [++]	2.80 (1.68,4.64)	⊕◯◯◯ VERY LOW
DBG-110MG vs. APX			1.17 (0.63,2.20)	⊕⊕◯◯ LOW [++]	1.17 (0.63,2.20)	⊕⊕◯◯ LOW
DBG-150MG vs. APX			0.85 (0.45,1.60)	⊕⊕◯◯ LOW [++]	0.85 (0.45,1.60)	⊕⊕◯◯ LOW
EDX-30MG vs. APX			1.40 (0.76,2.57)	⊕⊕◯◯ LOW [++]	1.40 (0.76,2.57)	⊕⊕◯◯ LOW
EDX-60MG vs. APX			1.08 (0.59,1.98)	⊕⊕◯◯ LOW [++]	1.08 (0.59,1.98)	⊕⊕◯◯ LOW
RVX vs. APX			1.05 (0.64,1.72)	⊕⊕◯◯ LOW [++]	1.05 (0.64,1.72)	⊕⊕◯◯ LOW
UC vs. APX	1.28 (1.06,1.52)	⊕⊕◯◯ LOW [a, e]	1.28 (0.61,2.69)	⊕◯◯◯ VERY LOW [§]	1.32 (0.89,1.95)	⊕⊕◯◯ LOW
UC+GNT vs. APX			0.44 (0.02,11.41)	⊕◯◯◯ VERY LOW [++]	0.44 (0.02,11.41)	⊕◯◯◯ VERY LOW
UC+SM vs. APX			0.31 (0.10,0.96)	⊕◯◯◯ VERY LOW [++]	0.31 (0.10,0.96)	⊕◯◯◯ VERY LOW
UC+ST vs. APX			1.31 (0.61,2.84)	⊕◯◯◯ VERY LOW [++]	1.31 (0.61,2.84)	⊕◯◯◯ VERY LOW
UC+WTH vs. APX			1.18 (0.53,2.63)	⊕⊕◯◯ LOW [++]	1.18 (0.53,2.63)	⊕⊕◯◯ LOW
ASA+CLP vs. ASA			1.08 (0.58,2.00)	⊕◯◯◯ VERY LOW [++]	1.08 (0.58,2.00)	⊕◯◯◯ VERY LOW
CONTROL vs. ASA	0.95 (0.68,1.35)	⊕⊕◯◯ LOW [a,b]	1.68 (1.00,2.80)	⊕◯◯◯ VERY LOW [j]	1.23 (0.85,1.78)	⊕⊕◯◯ LOW
DBG-110MG vs. ASA			0.52 (0.29,0.92)	⊕◯◯◯ VERY LOW [++]	0.52 (0.29,0.92)	⊕◯◯◯ VERY LOW
DBG-150MG vs. ASA			0.38 (0.21,0.67)	⊕◯◯◯ VERY LOW [++]	0.38 (0.21,0.67)	⊕◯◯◯ VERY LOW
EDX-30MG vs. ASA			0.61 (0.35,1.08)	⊕◯◯◯ VERY LOW [++]	0.61 (0.35,1.08)	⊕◯◯◯ VERY LOW
EDX-60MG vs. ASA			0.47 (0.27,0.83)	⊕◯◯◯ VERY LOW [++]	0.47 (0.27,0.83)	⊕◯◯◯ VERY LOW
RVX vs. ASA			0.46 (0.30,0.71)	⊕◯◯◯ VERY LOW [++]	0.46 (0.30,0.71)	⊕◯◯◯ VERY LOW
UC vs. ASA	0.61 (0.47,0.79)	⊕◯◯◯ VERY LOW [a,e,f,g]	0.48 (0.28,0.82)	⊕⊕◯◯ LOW [§]	0.58 (0.44,0.77)	⊕⊕◯◯ LOW
UC+GNT vs. ASA			0.19 (0.01,4.97)	⊕◯◯◯ VERY LOW [++]	0.19 (0.01,4.97)	⊕◯◯◯ VERY LOW
UC+SM vs. ASA			0.14 (0.05,0.41)	⊕◯◯◯ VERY LOW [++]	0.14 (0.05,0.41)	⊕◯◯◯ VERY LOW
UC+ST vs. ASA			0.58 (0.28,1.19)	⊕◯◯◯ VERY LOW [++]	0.58 (0.28,1.19)	⊕◯◯◯ VERY LOW

UC+WTH vs. ASA			0.52 (0.25,1.09)	⊕◯◯◯ VERY LOW [++]	0.52 (0.25,1.09)	⊕◯◯◯ VERY LOW
CONTROL vs. ASA+CLP			1.14 (0.60,2.19)	⊕⊕⊕◯ MODERATE [++]	1.14 (0.60,2.19)	⊕⊕⊕◯ MODERATE
DBG-110MG vs. ASA+CLP			0.48 (0.23,1.01)	⊕⊕◯◯ LOW [++]	0.48 (0.23,1.01)	⊕⊕◯◯ LOW
DBG-150MG vs. ASA+CLP			0.35 (0.17,0.73)	⊕⊕⊕◯ MODERATE [++]	0.35 (0.17,0.73)	⊕⊕⊕◯ MODERATE
EDX-30MG vs. ASA+CLP			0.57 (0.27,1.19)	⊕⊕◯◯ LOW [++]	0.57 (0.27,1.19)	⊕⊕◯◯ LOW
EDX-60MG vs. ASA+CLP			0.44 (0.21,0.92)	⊕⊕◯◯ LOW [++]	0.44 (0.21,0.92)	⊕⊕◯◯ LOW
RVX vs. ASA+CLP			0.43 (0.23,0.81)	⊕⊕⊕◯ MODERATE [++]	0.43 (0.23,0.81)	⊕⊕⊕◯ MODERATE
UC vs. ASA+CLP	0.54 (0.40,0.73)	⊕⊕⊕◯ MODERATE [a]	Not estimable	Not estimable *	0.54 (0.31,0.94)	⊕⊕⊕◯ MODERATE
UC+GNT vs. ASA+CLP			0.18 (0.01,4.78)	⊕◯◯◯ VERY LOW [++]	0.18 (0.01,4.78)	⊕◯◯◯ VERY LOW
UC+SM vs. ASA+CLP			0.13 (0.04,0.42)	⊕◯◯◯ VERY LOW [++]	0.13 (0.04,0.42)	⊕◯◯◯ VERY LOW
UC+ST vs. ASA+CLP			0.54 (0.23,1.27)	⊕◯◯◯ VERY LOW [++]	0.54 (0.23,1.27)	⊕◯◯◯ VERY LOW
UC+WTH vs. ASA+CLP			0.48 (0.20,1.16)	⊕⊕⊕◯ MODERATE [++]	0.48 (0.20,1.16)	⊕⊕⊕◯ MODERATE
DBG-110MG vs. CONTROL			0.42 (0.23,0.77)	⊕⊕◯◯ LOW [++]	0.42 (0.23,0.77)	⊕⊕◯◯ LOW
DBG-150MG vs. CONTROL			0.31 (0.16,0.57)	⊕⊕⊕◯ MODERATE [++]	0.31 (0.16,0.57)	⊕⊕⊕◯ MODERATE
EDX-30MG vs. CONTROL			0.50 (0.27,0.92)	⊕⊕◯◯ LOW [++]	0.50 (0.27,0.92)	⊕⊕◯◯ LOW
EDX-60MG vs. CONTROL			0.39 (0.21,0.71)	⊕⊕◯◯ LOW [++]	0.39 (0.21,0.71)	⊕⊕◯◯ LOW
RVX vs. CONTROL			0.37 (0.23,0.61)	⊕⊕⊕◯ MODERATE [++]	0.37 (0.23,0.61)	⊕⊕⊕◯ MODERATE
UC vs. CONTROL	0.37 (0.27,0.52)	⊕⊕⊕◯ MODERATE [a]	0.78 (0.42,1.45)	⊕◯◯◯ VERY LOW [§]	0.47 (0.33,0.67)	⊕⊕⊕◯ MODERATE
UC+GNT vs. CONTROL			0.16 (0.01,4.06)	⊕◯◯◯ VERY LOW [++]	0.16 (0.01,4.06)	⊕◯◯◯ VERY LOW
UC+SM vs. CONTROL			0.11 (0.04,0.34)	⊕◯◯◯ VERY LOW [++]	0.11 (0.04,0.34)	⊕◯◯◯ VERY LOW
UC+ST vs. CONTROL			0.47 (0.22,0.99)	⊕◯◯◯ VERY LOW [++]	0.47 (0.22,0.99)	⊕◯◯◯ VERY LOW
UC+WTH vs. CONTROL			0.42 (0.20,0.90)	⊕⊕⊕◯ MODERATE [++]	0.42 (0.20,0.90)	⊕⊕⊕◯ MODERATE
DBG-150MG vs. DBG-110MG	0.73 (0.58,0.91)	⊕⊕⊕◯ MODERATE [a]	0.31 (0.00,856.89)	⊕⊕◯◯ LOW [§]	0.73 (0.44,1.20)	⊕⊕⊕◯ MODERATE
EDX-30MG vs. DBG-110MG			1.19 (0.60,2.35)	⊕⊕◯◯ LOW [++]	1.19 (0.60,2.35)	⊕⊕◯◯ LOW
EDX-60MG vs. DBG-110MG			0.92 (0.46,1.81)	⊕⊕◯◯ LOW [++]	0.92 (0.46,1.81)	⊕⊕◯◯ LOW
RVX vs. DBG-110MG			0.89 (0.50,1.60)	⊕⊕◯◯ LOW [++]	0.89 (0.50,1.60)	⊕⊕◯◯ LOW
UC vs. DBG-110MG	1.10 (0.91,1.33)	⊕⊕◯◯ LOW [a,b]	2.63 (0.00,7,270.20)	⊕⊕⊕◯ MODERATE [¶]	1.12 (0.68,1.84)	⊕⊕⊕◯ MODERATE
UC+GNT vs. DBG-110MG			0.38 (0.01,9.85)	⊕◯◯◯ VERY LOW [++]	0.38 (0.01,9.85)	⊕◯◯◯ VERY LOW
UC+SM vs. DBG-110MG			0.27 (0.08,0.85)	⊕◯◯◯ VERY LOW [++]	0.27 (0.08,0.85)	⊕◯◯◯ VERY LOW
UC+ST vs. DBG-110MG			1.12 (0.49,2.56)	⊕◯◯◯ VERY LOW [++]	1.12 (0.49,2.56)	⊕◯◯◯ VERY LOW
UC+WTH vs. DBG-110MG			1.00 (0.43,2.36)	⊕⊕◯◯ LOW [++]	1.00 (0.43,2.36)	⊕⊕◯◯ LOW
EDX-30MG vs. DBG-150MG			1.63	⊕⊕◯◯	1.63	⊕⊕◯◯

			(0.82,3.24)	LOW [++]	(0.82,3.24)	LOW
EDX-60MG vs. DBG-150MG			1.26 (0.64,2.50)	⊕⊕◯◯ LOW [++]	1.26 (0.64,2.50)	⊕⊕◯◯ LOW
RVX vs. DBG-150MG			1.22 (0.68,2.20)	⊕⊕⊕◯ MODERATE [++]	1.22 (0.68,2.20)	⊕⊕⊕◯ MODERATE
UC vs. DBG-150MG	1.52 (1.22,1.85)	⊕⊕⊕◯ MODERATE [a]	1.54 (0.94,2.55)	⊕⊕◯◯ LOW [§]	1.54 (0.94,2.55)	⊕⊕⊕◯ MODERATE
UC+GNT vs. DBG-150MG			0.52 (0.02,13.56)	⊕◯◯◯ VERY LOW [++]	0.52 (0.02,13.56)	⊕◯◯◯ VERY LOW
UC+SM vs. DBG-150MG			0.37 (0.11,1.17)	⊕◯◯◯ VERY LOW [++]	0.37 (0.11,1.17)	⊕◯◯◯ VERY LOW
UC+ST vs. DBG-150MG			1.54 (0.67,3.53)	⊕◯◯◯ VERY LOW [++]	1.54 (0.67,3.53)	⊕◯◯◯ VERY LOW
UC+WTH vs. DBG-150MG			1.38 (0.58,3.26)	⊕⊕⊕◯ MODERATE [++]	1.38 (0.58,3.26)	⊕⊕⊕◯ MODERATE
EDX-30MG vs. DBG-150MG			1.63 (0.82,3.24)	⊕⊕◯◯ LOW [++]	1.63 (0.82,3.24)	⊕⊕◯◯ LOW
EDX-60MG vs. DBG-150MG			1.26 (0.64,2.50)	⊕⊕◯◯ LOW [++]	1.26 (0.64,2.50)	⊕⊕◯◯ LOW
RVX vs. DBG-150MG			1.22 (0.68,2.20)	⊕⊕⊕◯ MODERATE [++]	1.22 (0.68,2.20)	⊕⊕⊕◯ MODERATE
UC+GNT vs. DBG-150MG			0.52 (0.02,13.56)	⊕◯◯◯ VERY LOW [++]	0.52 (0.02,13.56)	⊕◯◯◯ VERY LOW
UC+SM vs. DBG-150MG			0.37 (0.11,1.17)	⊕◯◯◯ VERY LOW [++]	0.37 (0.11,1.17)	⊕◯◯◯ VERY LOW
UC+ST vs. DBG-150MG			1.54 (0.67,3.53)	⊕◯◯◯ VERY LOW [++]	1.54 (0.67,3.53)	⊕◯◯◯ VERY LOW
UC+WTH vs. DBG-150MG			1.38 (0.58,3.26)	⊕⊕⊕◯ MODERATE [++]	1.38 (0.58,3.26)	⊕⊕⊕◯ MODERATE
EDX-60MG vs. EDX-30MG	0.76 (0.66,0.88)	⊕⊕⊕◯ MODERATE [a]	0.77 (0.48,1.24)	⊕⊕◯◯ LOW [j]	0.77 (0.48,1.24)	⊕⊕⊕◯ MODERATE
RVX vs. EDX-30MG			0.75 (0.43,1.31)	⊕⊕◯◯ LOW [++]	0.75 (0.43,1.31)	⊕⊕◯◯ LOW
UC vs. EDX-30MG	0.88 (0.76,1.01)	⊕⊕◯◯ LOW [a,b]	0.95 (0.58,1.54)	⊕⊕◯◯ LOW [§]	0.95 (0.58,1.54)	⊕⊕◯◯ LOW
UC+GNT vs. EDX-30MG			0.32 (0.01,8.28)	⊕◯◯◯ VERY LOW [++]	0.32 (0.01,8.28)	⊕◯◯◯ VERY LOW
UC+SM vs. EDX-30MG			0.22 (0.07,0.72)	⊕◯◯◯ VERY LOW [++]	0.22 (0.07,0.72)	⊕◯◯◯ VERY LOW
UC+ST vs. EDX-30MG			0.94 (0.41,2.14)	⊕◯◯◯ VERY LOW [++]	0.94 (0.41,2.14)	⊕◯◯◯ VERY LOW
UC+WTH vs. EDX-30MG			0.84 (0.35,2.01)	⊕⊕◯◯ LOW [++]	0.84 (0.35,2.01)	⊕⊕◯◯ LOW
RVX vs. EDX-60MG			0.97 (0.56,1.69)	⊕⊕◯◯ LOW [++]	0.97 (0.56,1.69)	⊕⊕◯◯ LOW
UC vs. EDX-60MG	1.16 (0.99,1.35)	⊕⊕◯◯ LOW [a,b]	1.22 (0.76,1.98)	⊕⊕◯◯ LOW [§]	1.22 (0.76,1.98)	⊕⊕◯◯ LOW
UC+GNT vs. EDX-60MG			0.41 (0.02,10.72)	⊕◯◯◯ VERY LOW [++]	0.41 (0.02,10.72)	⊕◯◯◯ VERY LOW
UC+SM vs. EDX-60MG			0.29 (0.09,0.93)	⊕◯◯◯ VERY LOW [++]	0.29 (0.09,0.93)	⊕◯◯◯ VERY LOW
UC+ST vs. EDX-60MG			1.22 (0.54,2.76)	⊕◯◯◯ VERY LOW [++]	1.22 (0.54,2.76)	⊕◯◯◯ VERY LOW
UC+WTH vs. EDX-60MG			1.09 (0.46,2.58)	⊕⊕◯◯ LOW [++]	1.09 (0.46,2.58)	⊕⊕◯◯ LOW
UC vs. RVX	1.18 (1.02,1.35)	⊕⊕⊕◯ MODERATE [a]	Not estimable	Not estimable *	1.26 (0.91,1.75)	⊕⊕⊕◯ MODERATE
UC+GNT vs. RVX			0.42 (0.02,10.84)	⊕◯◯◯ VERY LOW [++]	0.42 (0.02,10.84)	⊕◯◯◯ VERY LOW
UC+SM vs. RVX			0.30 (0.10,0.90)	⊕◯◯◯ VERY LOW [++]	0.30 (0.10,0.90)	⊕◯◯◯ VERY LOW
UC+ST vs. RVX			1.25 (0.60,2.63)	⊕◯◯◯ VERY LOW [++]	1.25 (0.60,2.63)	⊕◯◯◯ VERY LOW

UC+WTH vs. RVX			1.13 (0.51,2.47)	⊕⊕⊕◯ MODERATE ++	1.13 (0.51,2.47)	⊕⊕⊕◯ MODERATE
UC+GNT vs. UC	0.33 (0.01,8.33)	⊕◯◯◯ VERY LOW [a,b,c,d,h]	Not estimable	Not estimable *	0.33 (0.01,8.45)	⊕◯◯◯ VERY LOW
UC+SM vs. UC	0.21 (0.08,0.57)	⊕◯◯◯ VERY LOW [a,c,d,h]	Not estimable	Not estimable *	0.24 (0.08,0.68)	⊕◯◯◯ VERY LOW
UC+ST vs. UC	0.99 (0.61,1.64)	⊕◯◯◯ VERY LOW [a,b,h]	Not estimable	Not estimable *	0.99 (0.51,1.93)	⊕◯◯◯ VERY LOW
UC+WTH vs. UC	0.90 (0.53,1.52)	⊕⊕⊕◯ MODERATE [a]	Not estimable	Not estimable *	0.89 (0.45,1.78)	⊕⊕⊕◯ MODERATE
UC+SM vs. UC+GNT			0.71 (0.02,21.08)	⊕◯◯◯ VERY LOW ++	0.71 (0.02,21.08)	⊕◯◯◯ VERY LOW
UC+ST vs. UC+GNT			2.97 (0.11,80.15)	⊕◯◯◯ VERY LOW ++	2.97 (0.11,80.15)	⊕◯◯◯ VERY LOW
UC+WTH vs. UC+GNT			2.67 (0.10,72.41)	⊕◯◯◯ VERY LOW ++	2.67 (0.10,72.41)	⊕◯◯◯ VERY LOW
UC+ST vs. UC+SM			4.20 (1.21,14.57)	⊕◯◯◯ VERY LOW ++	4.20 (1.21,14.57)	⊕◯◯◯ VERY LOW
UC+WTH vs. UC+SM			3.77 (1.08,13.21)	⊕◯◯◯ VERY LOW ++	3.77 (1.08,13.21)	⊕◯◯◯ VERY LOW
**All-cause mortality**						
ASA vs. APX	1.27 (0.99,1.61)	⊕⊕⊕◯ MODERATE [b]	1.16 (0.94,1.42)	⊕◯◯◯ VERY LOW §	1.20 (1.03,1.41)	⊕⊕⊕◯ MODERATE
ASA+CLP vs. APX			1.12 (0.88,1.42)	⊕⊕◯◯ LOW ++	1.12 (0.88,1.42)	⊕⊕◯◯ LOW
CONTROL vs. APX			1.26 (0.89,1.78)	⊕⊕◯◯ LOW ++	1.26 (0.89,1.78)	⊕⊕◯◯ LOW
DBG-110MG vs. APX			1.03 (0.88,1.21)	⊕⊕◯◯ LOW ++	1.03 (0.88,1.21)	⊕⊕◯◯ LOW
DBG-150MG vs. APX			1.00 (0.86,1.18)	⊕⊕◯◯ LOW ++	1.00 (0.86,1.18)	⊕⊕◯◯ LOW
EDX-30MG vs. APX			0.99 (0.86,1.13)	⊕⊕⊕◯ MODERATE ++	0.99 (0.86,1.13)	⊕⊕⊕◯ MODERATE
EDX-60MG vs. APX			1.04 (0.91,1.19)	⊕⊕⊕◯ MODERATE ++	1.04 (0.91,1.19)	⊕⊕⊕◯ MODERATE
RVX vs. APX			0.95 (0.78,1.17)	⊕⊕⊕◯ MODERATE ++	0.95 (0.78,1.17)	⊕⊕⊕◯ MODERATE
UC vs. APX	1.11 (1.00,1.23)	⊕⊕⊕◯ MODERATE [b]	1.22 (0.91,1.65)	⊕◯◯◯ VERY LOW §	1.13 (1.02,1.24)	⊕⊕⊕◯ MODERATE
UC+GNT vs. APX			2.83 (0.55,14.47)	⊕◯◯◯ VERY LOW ++	2.83 (0.55,14.47)	⊕◯◯◯ VERY LOW
UC+SM vs. APX			0.47 (0.19,1.18)	⊕◯◯◯ VERY LOW ++	0.47 (0.19,1.18)	⊕◯◯◯ VERY LOW
UC+ST vs. APX			1.08 (0.86,1.37)	⊕◯◯◯ VERY LOW ++	1.08 (0.86,1.37)	⊕◯◯◯ VERY LOW
UC+WTH vs. APX			0.55 (0.31,0.97)	⊕⊕⊕◯ MODERATE ++	0.55 (0.31,0.97)	⊕⊕⊕◯ MODERATE
ASA+CLP vs. ASA			0.93 (0.72,1.21)	⊕◯◯◯ VERY LOW ++	0.93 (0.72,1.21)	⊕◯◯◯ VERY LOW
CONTROL vs. ASA	0.86 (0.35,2.10)	⊕⊕◯◯ LOW [a,b]	1.08 (0.73,1.60)	⊕◯◯◯ VERY LOW §	1.04 (0.73,1.49)	⊕⊕◯◯ LOW
DBG-110MG vs. ASA			0.86 (0.71,1.04)	⊕◯◯◯ VERY LOW ++	0.86 (0.71,1.04)	⊕◯◯◯ VERY LOW
DBG-150MG vs. ASA			0.84 (0.69,1.01)	⊕◯◯◯ VERY LOW ++	0.84 (0.69,1.01)	⊕◯◯◯ VERY LOW
EDX-30MG vs. ASA			0.82 (0.69,0.98)	⊕◯◯◯ VERY LOW ++	0.82 (0.69,0.98)	⊕◯◯◯ VERY LOW
EDX-60MG vs. ASA			0.86 (0.73,1.03)	⊕◯◯◯ VERY LOW ++	0.86 (0.73,1.03)	⊕◯◯◯ VERY LOW
RVX vs. ASA			0.79 (0.63,1.00)	⊕◯◯◯ VERY LOW ++	0.79 (0.63,1.00)	⊕◯◯◯ VERY LOW

UC vs. ASA	0.97 (0.81,1.16)	⊕◯◯◯ VERY LOW [a,b,f,g]	0.87 (0.67,1.12)	⊕⊕⊕◯ MODERATE [b]	0.94 (0.81,1.09)	⊕⊕⊕◯ MODERATE
UC+GNT vs. ASA			2.35 (0.46,12.08)	⊕◯◯◯ VERY LOW [++]	2.35 (0.46,12.08)	⊕◯◯◯ VERY LOW
UC+SM vs. ASA			0.39 (0.16,0.99)	⊕◯◯◯ VERY LOW [++]	0.39 (0.16,0.99)	⊕◯◯◯ VERY LOW
UC+ST vs. ASA			0.90 (0.70,1.17)	⊕◯◯◯ VERY LOW [++]	0.90 (0.70,1.17)	⊕◯◯◯ VERY LOW
UC+WTH vs. ASA			0.46 (0.26,0.82)	⊕◯◯◯ VERY LOW [++]	0.46 (0.26,0.82)	⊕◯◯◯ VERY LOW
CONTROL vs. ASA+CLP			1.12 (0.75,1.67)	⊕⊕◯◯ LOW [++]	1.12 (0.75,1.67)	⊕⊕◯◯ LOW
DBG-110MG vs. ASA+CLP			0.92 (0.72,1.18)	⊕⊕◯◯ LOW [++]	0.92 (0.72,1.18)	⊕⊕◯◯ LOW
DBG-150MG vs. ASA+CLP			0.90 (0.70,1.15)	⊕⊕◯◯ LOW [++]	0.90 (0.70,1.15)	⊕⊕◯◯ LOW
EDX-30MG vs. ASA+CLP			0.88 (0.70,1.12)	⊕⊕◯◯ LOW [++]	0.88 (0.70,1.12)	⊕⊕◯◯ LOW
EDX-60MG vs. ASA+CLP			0.93 (0.73,1.17)	⊕⊕◯◯ LOW [++]	0.93 (0.73,1.17)	⊕⊕◯◯ LOW
RVX vs. ASA+CLP			0.85 (0.64,1.12)	⊕⊕◯◯ LOW [++]	0.85 (0.64,1.12)	⊕⊕◯◯ LOW
UC vs. ASA+CLP	1.00 (0.81,1.25)	⊕⊕◯◯ LOW [a,b]	Not estimable	Not estimable *	1.00 (0.81,1.25)	⊕⊕◯◯ LOW
UC+GNT vs. ASA+CLP			2.52 (0.49,13.05)	⊕◯◯◯ VERY LOW [++]	2.52 (0.49,13.05)	⊕◯◯◯ VERY LOW
UC+SM vs. ASA+CLP			0.42 (0.17,1.07)	⊕◯◯◯ VERY LOW [++]	0.42 (0.17,1.07)	⊕◯◯◯ VERY LOW
UC+ST vs. ASA+CLP			0.97 (0.72,1.31)	⊕◯◯◯ VERY LOW [++]	0.97 (0.72,1.31)	⊕◯◯◯ VERY LOW
UC+WTH vs. ASA+CLP			0.49 (0.27,0.90)	⊕⊕◯◯ LOW [++]	0.49 (0.27,0.90)	⊕⊕◯◯ LOW
DBG-110MG vs. CONTROL			0.82 (0.58,1.17)	⊕⊕◯◯ LOW [++]	0.82 (0.58,1.17)	⊕⊕◯◯ LOW
DBG-150MG vs. CONTROL			0.80 (0.56,1.14)	⊕⊕◯◯ LOW [++]	0.80 (0.56,1.14)	⊕⊕◯◯ LOW
EDX-30MG vs. CONTROL			0.79 (0.56,1.11)	⊕⊕◯◯ LOW [++]	0.79 (0.56,1.11)	⊕⊕◯◯ LOW
EDX-60MG vs. CONTROL			0.83 (0.58,1.17)	⊕⊕◯◯ LOW [++]	0.83 (0.58,1.17)	⊕⊕◯◯ LOW
RVX vs. CONTROL			0.76 (0.52,1.11)	⊕⊕◯◯ LOW [++]	0.76 (0.52,1.11)	⊕⊕◯◯ LOW
UC vs. CONTROL	0.87 (0.61,1.23)	⊕⊕◯◯ LOW [a,b]	1.09 (0.44,2.70)	⊕◯◯◯ VERY LOW [§]	0.90 (0.64,1.25)	⊕⊕◯◯ LOW
UC+GNT vs. CONTROL			2.25 (0.43,11.88)	⊕◯◯◯ VERY LOW [++]	2.25 (0.43,11.88)	⊕◯◯◯ VERY LOW
UC+SM vs. CONTROL			0.38 (0.14,0.99)	⊕◯◯◯ VERY LOW [++]	0.38 (0.14,0.99)	⊕◯◯◯ VERY LOW
UC+ST vs. CONTROL			0.86 (0.58,1.28)	⊕◯◯◯ VERY LOW [++]	0.86 (0.58,1.28)	⊕◯◯◯ VERY LOW
UC+WTH vs. CONTROL			0.44 (0.23,0.84)	⊕⊕◯◯ LOW [++]	0.44 (0.23,0.84)	⊕⊕◯◯ LOW
DBG-150MG vs. DBG-110MG	0.97 (0.85,1.10)	⊕⊕◯◯ LOW [a,b]	0.97 (0.86,1.10)	⊕⊕◯◯ LOW [§]	0.97 (0.86,1.10)	⊕⊕◯◯ LOW
EDX-30MG vs. DBG-110MG			0.96 (0.82,1.12)	⊕⊕◯◯ LOW [++]	0.96 (0.82,1.12)	⊕⊕◯◯ LOW
EDX-60MG vs. DBG-110MG			1.00 (0.86,1.17)	⊕⊕◯◯ LOW [++]	1.00 (0.86,1.17)	⊕⊕◯◯ LOW
RVX vs. DBG-110MG			0.92 (0.74,1.15)	⊕⊕◯◯ LOW [++]	0.92 (0.74,1.15)	⊕⊕◯◯ LOW
UC vs. DBG-110MG	1.09 (0.96,1.23)	⊕⊕◯◯ LOW [a,b]	1.09 (0.96,1.23)	⊕⊕◯◯ LOW [§]	1.09 (0.96,1.23)	⊕⊕◯◯ LOW

UC+GNT vs. DBG-110MG			2.74 (0.53,14.03)	⊕◯◯◯ VERY LOW [++]	2.74 (0.53,14.03)	⊕◯◯◯ VERY LOW
UC+SM vs. DBG-110MG			0.46 (0.18,1.15)	⊕◯◯◯ VERY LOW [++]	0.46 (0.18,1.15)	⊕◯◯◯ VERY LOW
UC+ST vs. DBG-110MG			1.05 (0.82,1.34)	⊕◯◯◯ VERY LOW [++]	1.05 (0.82,1.34)	⊕◯◯◯ VERY LOW
UC+WTH vs. DBG-110MG			0.54 (0.30,0.95)	⊕⊕◯◯ LOW [++]	0.54 (0.30,0.95)	⊕⊕◯◯ LOW
EDX-30MG vs. DBG-150MG			0.99 (0.84,1.15)	⊕⊕◯◯ LOW [++]	0.99 (0.84,1.15)	⊕⊕◯◯ LOW
EDX-60MG vs. DBG-150MG			1.03 (0.89,1.21)	⊕⊕◯◯ LOW [++]	1.03 (0.89,1.21)	⊕⊕◯◯ LOW
RVX vs. DBG-150MG			0.95 (0.76,1.18)	⊕⊕◯◯ LOW [++]	0.95 (0.76,1.18)	⊕⊕◯◯ LOW
UC vs. DBG-150MG	1.12 (0.99,1.26)	⊕⊕◯◯ LOW [a,b]	1.12 (0.99,1.27)	⊕⊕◯◯ LOW [§]	1.12 (0.99,1.27)	⊕⊕◯◯ LOW
UC+GNT vs. DBG-150MG			2.82 (0.55,14.44)	⊕◯◯◯ VERY LOW [++]	2.82 (0.55,14.44)	⊕◯◯◯ VERY LOW
UC+SM vs. DBG-150MG			0.47 (0.19,1.18)	⊕◯◯◯ VERY LOW [++]	0.47 (0.19,1.18)	⊕◯◯◯ VERY LOW
UC+ST vs. DBG-150MG			1.08 (0.85,1.38)	⊕◯◯◯ VERY LOW [++]	1.08 (0.85,1.38)	⊕◯◯◯ VERY LOW
UC+WTH vs. DBG-150MG			0.55 (0.31,0.98)	⊕⊕◯◯ LOW [++]	0.55 (0.31,0.98)	⊕⊕◯◯ LOW
EDX-30MG vs. DBG-150MG			0.99 (0.84,1.15)	⊕⊕◯◯ LOW [++]	0.99 (0.84,1.15)	⊕⊕◯◯ LOW
EDX-60MG vs. DBG-150MG			1.03 (0.89,1.21)	⊕⊕◯◯ LOW [++]	1.03 (0.89,1.21)	⊕⊕◯◯ LOW
RVX vs. DBG-150MG			0.95 (0.76,1.18)	⊕⊕◯◯ LOW [++]	0.95 (0.76,1.18)	⊕⊕◯◯ LOW
UC+GNT vs. DBG-150MG			2.82 (0.55,14.44)	⊕◯◯◯ VERY LOW [++]	2.82 (0.55,14.44)	⊕◯◯◯ VERY LOW
UC+SM vs. DBG-150MG			0.47 (0.19,1.18)	⊕◯◯◯ VERY LOW [++]	0.47 (0.19,1.18)	⊕◯◯◯ VERY LOW
UC+ST vs. DBG-150MG			1.08 (0.85,1.38)	⊕◯◯◯ VERY LOW [++]	1.08 (0.85,1.38)	⊕◯◯◯ VERY LOW
UC+WTH vs. DBG-150MG			0.55 (0.31,0.98)	⊕⊕◯◯ LOW [++]	0.55 (0.31,0.98)	⊕⊕◯◯ LOW
EDX-60MG vs. EDX-30MG	1.05 (0.95,1.15)	⊕⊕⊕◯ MODERATE [b]	1.05 (0.95,1.15)	⊕⊕⊕◯ MODERATE [¶]	1.05 (0.95,1.15)	⊕⊕⊕◯ MODERATE
RVX vs. EDX-30MG			0.96 (0.79,1.18)	⊕⊕⊕◯ MODERATE [++]	0.96 (0.79,1.18)	⊕⊕⊕◯ MODERATE
UC vs. EDX-30MG	1.14 (1.03,1.25)	⊕⊕⊕⊕ HIGH	1.14 (1.04,1.25)	⊕⊕⊕◯ MODERATE [¶]	1.14 (1.04,1.25)	⊕⊕⊕⊕ HIGH
UC+GNT vs. EDX-30MG			2.86 (0.56,14.62)	⊕◯◯◯ VERY LOW [++]	2.86 (0.56,14.62)	⊕◯◯◯ VERY LOW
UC+SM vs. EDX-30MG			0.48 (0.19,1.19)	⊕◯◯◯ VERY LOW [++]	0.48 (0.19,1.19)	⊕◯◯◯ VERY LOW
UC+ST vs. EDX-30MG			1.10 (0.87,1.38)	⊕◯◯◯ VERY LOW [++]	1.10 (0.87,1.38)	⊕◯◯◯ VERY LOW
UC+WTH vs. EDX-30MG			0.56 (0.32,0.98)	⊕⊕⊕◯ MODERATE [++]	0.56 (0.32,0.98)	⊕⊕⊕◯ MODERATE
RVX vs. EDX-60MG			0.92 (0.75,1.12)	⊕⊕⊕◯ MODERATE [++]	0.92 (0.75,1.12)	⊕⊕⊕◯ MODERATE
UC vs. EDX-60MG	1.09 (0.99,1.19)	⊕⊕⊕◯ MODERATE [b]	1.09 (0.99,1.19)	⊕⊕⊕◯ MODERATE [¶]	1.09 (0.99,1.19)	⊕⊕⊕◯ MODERATE
UC+GNT vs. EDX-60MG			2.73 (0.53,13.94)	⊕◯◯◯ VERY LOW [++]	2.73 (0.53,13.94)	⊕◯◯◯ VERY LOW
UC+SM vs. EDX-60MG			0.46 (0.18,1.14)	⊕◯◯◯ VERY LOW [++]	0.46 (0.18,1.14)	⊕◯◯◯ VERY LOW
UC+ST vs. EDX-60MG			1.04 (0.83,1.32)	⊕◯◯◯ VERY LOW [++]	1.04 (0.83,1.32)	⊕◯◯◯ VERY LOW

UC+WTH vs. EDX-60MG			0.53 (0.30,0.94)	⊕⊕⊕◯ MODERATE [++]	0.53 (0.30,0.94)	⊕⊕⊕◯ MODERATE
UC vs. RVX	1.18 (0.99,1.41)	⊕⊕⊕◯ MODERATE [b]	Not estimable	Not estimable [*]	1.18 (0.99,1.41)	⊕⊕⊕◯ MODERATE
UC+GNT vs. RVX			2.97 (0.58,15.30)	⊕◯◯◯ VERY LOW [++]	2.97 (0.58,15.30)	⊕◯◯◯ VERY LOW
UC+SM vs. RVX			0.50 (0.20,1.25)	⊕◯◯◯ VERY LOW [++]	0.50 (0.20,1.25)	⊕◯◯◯ VERY LOW
UC+ST vs. RVX			1.14 (0.86,1.50)	⊕◯◯◯ VERY LOW [++]	1.14 (0.86,1.50)	⊕◯◯◯ VERY LOW
UC+WTH vs. RVX			0.58 (0.32,1.04)	⊕⊕⊕◯ MODERATE [++]	0.58 (0.32,1.04)	⊕⊕⊕◯ MODERATE
UC+GNT vs. UC	2.50 (0.49,12.50)	⊕◯◯◯ VERY LOW [a,b,c,d,h]	Not estimable	Not estimable [*]	2.51 (0.49,12.81)	⊕◯◯◯ VERY LOW
UC+SM vs. UC	0.40 (0.16,1.02)	⊕◯◯◯ VERY LOW [a,b,h]	Not estimable	Not estimable [*]	0.42 (0.17,1.04)	⊕◯◯◯ VERY LOW
UC+ST vs. UC	0.96 (0.78,1.19)	⊕◯◯◯ VERY LOW [a,b,h]	Not estimable	Not estimable [*]	0.96 (0.78,1.19)	⊕◯◯◯ VERY LOW
UC+WTH vs. UC	0.50 (0.29,0.87)	⊕⊕⊕◯ MODERATE [e]	Not estimable	Not estimable [*]	0.49 (0.28,0.86)	⊕⊕⊕◯ MODERATE
UC+SM vs. UC+GNT			0.17 (0.03,1.08)	⊕◯◯◯ VERY LOW [++]	0.17 (0.03,1.08)	⊕◯◯◯ VERY LOW
UC+ST vs. UC+GNT			0.38 (0.07,1.98)	⊕◯◯◯ VERY LOW [++]	0.38 (0.07,1.98)	⊕◯◯◯ VERY LOW
UC+WTH vs. UC+GNT			0.20 (0.03,1.10)	⊕◯◯◯ VERY LOW [++]	0.20 (0.03,1.10)	⊕◯◯◯ VERY LOW
UC+ST vs. UC+SM			2.29 (0.90,5.84)	⊕◯◯◯ VERY LOW [++]	2.29 (0.90,5.84)	⊕◯◯◯ VERY LOW
UC+WTH vs. UC+SM			1.17 (0.40,3.40)	⊕◯◯◯ VERY LOW [++]	1.17 (0.40,3.40)	⊕◯◯◯ VERY LOW
**Major Bleeding**						
ASA vs. APX	0.89 (0.58,1.37)	⊕⊕⊕◯ MODERATE [b]	0.89 (0.26,3.03)	⊕◯◯◯ VERY LOW [§]	0.89 (0.60,1.34)	⊕⊕⊕◯ MODERATE
ASA+CLP vs. APX			1.53 (1.12,2.08)	⊕⊕◯◯ LOW [++]	1.53 (1.12,2.08)	⊕⊕◯◯ LOW
CONTROL vs. APX			0.43 (0.09,2.11)	⊕◯◯◯ VERY LOW [++]	0.43 (0.09,2.11)	⊕◯◯◯ VERY LOW
DBG-110MG vs. APX			1.16 (0.95,1.41)	⊕⊕⊕◯ MODERATE [++]	1.16 (0.95,1.41)	⊕⊕⊕◯ MODERATE
DBG-150MG vs. APX			1.34 (1.10,1.62)	⊕⊕◯◯ LOW [++]	1.34 (1.10,1.62)	⊕⊕◯◯ LOW
EDX-30MG vs. APX			0.69 (0.56,0.84)	⊕⊕⊕◯ MODERATE [++]	0.69 (0.56,0.84)	⊕⊕⊕◯ MODERATE
EDX-60MG vs. APX			1.14 (0.94,1.37)	⊕⊕⊕◯ MODERATE [++]	1.14 (0.94,1.37)	⊕⊕⊕◯ MODERATE
RVX vs. APX			1.44 (1.19,1.74)	⊕⊕◯◯ LOW [++]	1.44 (1.19,1.74)	⊕⊕◯◯ LOW
UC vs. APX	1.43 (1.23,1.64)	⊕⊕⊕◯ MODERATE [a]	1.42 (0.39,5.18)	⊕◯◯◯ VERY LOW [§]	1.42 (1.24,1.63)	⊕⊕⊕◯ MODERATE
UC+GNT vs. APX			1.43 (0.03,71.88)	⊕⊕⊕◯ MODERATE [++]	1.43 (0.03,71.88)	⊕⊕⊕◯ MODERATE
UC+SM vs. APX			2.89 (0.27,31.18)	⊕⊕◯◯ LOW [++]	2.89 (0.27,31.18)	⊕⊕◯◯ LOW
UC+WTH vs. APX			0.92 (0.49,1.70)	⊕⊕⊕◯ MODERATE [++]	0.92 (0.49,1.70)	⊕⊕⊕◯ MODERATE
ASA+CLP vs. ASA			1.71 (1.03,2.84)	⊕◯◯◯ VERY LOW [++]	1.71 (1.03,2.84)	⊕◯◯◯ VERY LOW
CONTROL vs. ASA			0.48 (0.09,2.48)	⊕◯◯◯ VERY LOW [++]	0.48 (0.09,2.48)	⊕◯◯◯ VERY LOW
DBG-110MG vs. ASA			1.30 (0.83,2.03)	⊕◯◯◯ VERY LOW [++]	1.30 (0.83,2.03)	⊕◯◯◯ VERY LOW

DBG-150MG vs. ASA			1.50 (0.96,2.33)	⊕◯◯◯ VERY LOW ++	1.50 (0.96,2.33)	⊕◯◯◯ VERY LOW
EDX-30MG vs. ASA			0.77 (0.49,1.20)	⊕◯◯◯ VERY LOW ++	0.77 (0.49,1.20)	⊕◯◯◯ VERY LOW
EDX-60MG vs. ASA			1.28 (0.82,1.98)	⊕◯◯◯ VERY LOW ++	1.28 (0.82,1.98)	⊕◯◯◯ VERY LOW
RVX vs. ASA			1.62 (1.04,2.52)	⊕◯◯◯ VERY LOW ++	1.62 (1.04,2.52)	⊕◯◯◯ VERY LOW
UC vs. ASA	1.54 (0.56,4.20)	⊕◯◯◯ VERY LOW [a,b,d,f,g]	1.60 (1.02,2.50)	⊕⊕⊕◯ MODERATE ¶	1.60 (1.05,2.43)	⊕⊕⊕◯ MODERATE
UC+GNT vs. ASA			1.60 (0.03,82.26)	⊕◯◯◯ VERY LOW ++	1.60 (0.03,82.26)	⊕◯◯◯ VERY LOW
UC+SM vs. ASA			3.25 (0.29,36.15)	⊕◯◯◯ VERY LOW ++	3.25 (0.29,36.15)	⊕◯◯◯ VERY LOW
UC+WTH vs. ASA			1.03 (0.49,2.15)	⊕◯◯◯ VERY LOW ++	1.03 (0.49,2.15)	⊕◯◯◯ VERY LOW
CONTROL vs. ASA+CLP			0.28 (0.06,1.41)	⊕◯◯◯ VERY LOW ++	0.28 (0.06,1.41)	⊕◯◯◯ VERY LOW
DBG-110MG vs. ASA+CLP			0.76 (0.55,1.03)	⊕⊕◯◯ LOW ++	0.76 (0.55,1.03)	⊕⊕◯◯ LOW
DBG-150MG vs. ASA+CLP			0.87 (0.64,1.19)	⊕⊕◯◯ LOW ++	0.87 (0.64,1.19)	⊕⊕◯◯ LOW
EDX-30MG vs. ASA+CLP			0.45 (0.33,0.62)	⊕⊕◯◯ LOW ++	0.45 (0.33,0.62)	⊕⊕◯◯ LOW
EDX-60MG vs. ASA+CLP			0.74 (0.55,1.01)	⊕⊕◯◯ LOW ++	0.74 (0.55,1.01)	⊕⊕◯◯ LOW
RVX vs. ASA+CLP			0.94 (0.69,1.28)	⊕⊕◯◯ LOW ++	0.94 (0.69,1.28)	⊕⊕◯◯ LOW
UC vs. ASA+CLP	0.93 (0.70,1.23)	⊕⊕◯◯ LOW [a,b]	Not estimable	Not estimable *	0.93 (0.71,1.23)	⊕⊕◯◯ LOW
UC+GNT vs. ASA+CLP			0.93 (0.02,47.37)	⊕⊕◯◯ LOW ++	0.93 (0.02,47.37)	⊕⊕◯◯ LOW
UC+SM vs. ASA+CLP			1.89 (0.17,20.65)	⊕⊕◯◯ LOW ++	1.89 (0.17,20.65)	⊕⊕◯◯ LOW
UC+WTH vs. ASA+CLP			0.60 (0.31,1.16)	⊕⊕◯◯ LOW ++	0.60 (0.31,1.16)	⊕⊕◯◯ LOW
DBG-110MG vs. CONTROL			2.72 (0.55,13.46)	⊕◯◯◯ VERY LOW ++	2.72 (0.55,13.46)	⊕◯◯◯ VERY LOW
DBG-150MG vs. CONTROL			3.14 (0.63,15.54)	⊕◯◯◯ VERY LOW ++	3.14 (0.63,15.54)	⊕◯◯◯ VERY LOW
EDX-30MG vs. CONTROL			1.61 (0.33,8.00)	⊕◯◯◯ VERY LOW ++	1.61 (0.33,8.00)	⊕◯◯◯ VERY LOW
EDX-60MG vs. CONTROL			2.67 (0.54,13.21)	⊕◯◯◯ VERY LOW ++	2.67 (0.54,13.21)	⊕◯◯◯ VERY LOW
RVX vs. CONTROL			3.39 (0.68,16.76)	⊕◯◯◯ VERY LOW ++	3.39 (0.68,16.76)	⊕◯◯◯ VERY LOW
UC vs. CONTROL	3.34 (0.68,16.45)	⊕◯◯◯ VERY LOW [a,b,c,d]	Not estimable	Not estimable *	3.34 (0.68,16.45)	⊕◯◯◯ VERY LOW
UC+GNT vs. CONTROL			3.36 (0.05,230.02)	⊕◯◯◯ VERY LOW ++	3.36 (0.05,230.02)	⊕◯◯◯ VERY LOW
UC+SM vs. CONTROL			6.80 (0.39,118.51)	⊕◯◯◯ VERY LOW ++	6.80 (0.39,118.51)	⊕◯◯◯ VERY LOW
UC+WTH vs. CONTROL			2.15 (0.39,11.83)	⊕◯◯◯ VERY LOW ++	2.15 (0.39,11.83)	⊕◯◯◯ VERY LOW
DBG-150MG vs. DBG-110MG	1.15 (1.00,1.33)	⊕⊕◯◯ LOW [a,b]	0.05 (0.00,17.53)	⊕⊕◯◯ LOW §	1.15 (1.00,1.33)	⊕⊕◯◯ LOW
EDX-30MG vs. DBG-110MG			0.59 (0.48,0.73)	⊕⊕⊕◯ MODERATE ++	0.59 (0.48,0.73)	⊕⊕⊕◯ MODERATE
EDX-60MG vs. DBG-110MG			0.98 (0.81,1.19)	⊕⊕⊕◯ MODERATE ++	0.98 (0.81,1.19)	⊕⊕⊕◯ MODERATE

RVX vs. DBG-110MG			1.25 (1.03,1.51)	⊕⊕◯◯ LOW [++]	1.25 (1.03,1.51)	⊕⊕◯◯ LOW
UC vs. DBG-110MG	1.23 (1.06,1.43)	⊕⊕⊕◯ MODERATE [a]	0.07 (0.00,24.90)	⊕⊕◯◯ LOW [§]	1.23 (1.07,1.42)	⊕⊕⊕◯ MODERATE
UC+GNT vs. DBG-110MG			1.24 (0.02,62.14)	⊕⊕⊕◯ MODERATE [++]	1.24 (0.02,62.14)	⊕⊕⊕◯ MODERATE
UC+SM vs. DBG-110MG			2.50 (0.23,26.96)	⊕⊕◯◯ LOW [++]	2.50 (0.23,26.96)	⊕⊕◯◯ LOW
UC+WTH vs. DBG-110MG			0.79 (0.43,1.47)	⊕⊕⊕◯ MODERATE [++]	0.79 (0.43,1.47)	⊕⊕⊕◯ MODERATE
EDX-30MG vs. DBG-150MG			0.51 (0.42,0.63)	⊕⊕◯◯ LOW [++]	0.51 (0.42,0.63)	⊕⊕◯◯ LOW
EDX-60MG vs. DBG-150MG			0.85 (0.71,1.02)	⊕⊕◯◯ LOW [++]	0.85 (0.71,1.02)	⊕⊕◯◯ LOW
RVX vs. DBG-150MG			1.08 (0.89,1.30)	⊕⊕◯◯ LOW [++]	1.08 (0.89,1.30)	⊕⊕◯◯ LOW
UC vs. DBG-150MG	1.06 (0.93,1.22)	⊕⊕◯◯ LOW [a,b]	1.06 (0.93,1.22)	⊕⊕◯◯ LOW [§]	1.06 (0.93,1.22)	⊕⊕◯◯ LOW
UC+GNT vs. DBG-150MG			1.07 (0.02,53.80)	⊕⊕◯◯ LOW [++]	1.07 (0.02,53.80)	⊕⊕◯◯ LOW
UC+SM vs. DBG-150MG			2.17 (0.20,23.33)	⊕⊕◯◯ LOW [++]	2.17 (0.20,23.33)	⊕⊕◯◯ LOW
UC+WTH vs. DBG-150MG			0.69 (0.37,1.27)	⊕⊕◯◯ LOW [++]	0.69 (0.37,1.27)	⊕⊕◯◯ LOW
EDX-60MG vs. EDX-30MG	1.67 (1.43,1.92)	⊕⊕⊕◯ MODERATE [a]	1.65 (1.42,1.92)	⊕⊕⊕◯ MODERATE [¶]	1.65 (1.42,1.92)	⊕⊕⊕◯ MODERATE
RVX vs. EDX-30MG			2.10 (1.72,2.55)	⊕⊕◯◯ LOW [++]	2.10 (1.72,2.55)	⊕⊕◯◯ LOW
UC vs. EDX-30MG	2.08 (1.79,2.38)	⊕⊕⊕◯ MODERATE [a]	2.07 (1.79,2.39)	⊕⊕⊕◯ MODERATE [¶]	2.07 (1.79,2.39)	⊕⊕⊕◯ MODERATE
UC+GNT vs. EDX-30MG			2.08 (0.04,104.57)	⊕⊕⊕◯ MODERATE [++]	2.08 (0.04,104.57)	⊕⊕⊕◯ MODERATE
UC+SM vs. EDX-30MG			4.21 (0.39,45.37)	⊕⊕◯◯ LOW [++]	4.21 (0.39,45.37)	⊕⊕◯◯ LOW
UC+WTH vs. EDX-30MG			1.33 (0.72,2.48)	⊕⊕⊕◯ MODERATE [++]	1.33 (0.72,2.48)	⊕⊕⊕◯ MODERATE
RVX vs. EDX-60MG			1.27 (1.06,1.52)	⊕⊕◯◯ LOW [++]	1.27 (1.06,1.52)	⊕⊕◯◯ LOW
UC vs. EDX-60MG	1.25 (1.10,1.41)	⊕⊕⊕◯ MODERATE [a]	1.25 (1.11,1.42)	⊕⊕⊕◯ MODERATE [¶]	1.25 (1.11,1.42)	⊕⊕⊕◯ MODERATE
UC+GNT vs. EDX-60MG			1.26 (0.02,63.19)	⊕⊕⊕◯ MODERATE [++]	1.26 (0.02,63.19)	⊕⊕⊕◯ MODERATE
UC+SM vs. EDX-60MG			2.55 (0.24,27.40)	⊕⊕◯◯ LOW [++]	2.55 (0.24,27.40)	⊕⊕◯◯ LOW
UC+WTH vs. EDX-60MG			0.81 (0.44,1.49)	⊕⊕⊕◯ MODERATE [++]	0.81 (0.44,1.49)	⊕⊕⊕◯ MODERATE
UC vs. RVX	1.00 (0.88,1.14)	⊕⊕◯◯ LOW [a,b]	Not estimable	Not estimable *	0.99 (0.87,1.12)	⊕⊕◯◯ LOW
UC+GNT vs. RVX			0.99 (0.02,49.83)	⊕⊕◯◯ LOW [++]	0.99 (0.02,49.83)	⊕⊕◯◯ LOW
UC+SM vs. RVX			2.01 (0.19,21.61)	⊕⊕◯◯ LOW [++]	2.01 (0.19,21.61)	⊕⊕◯◯ LOW
UC+WTH vs. RVX			0.64 (0.34,1.18)	⊕⊕◯◯ LOW [++]	0.64 (0.34,1.18)	⊕⊕◯◯ LOW
UC+SM vs. UC	2.04 (0.19,1.18)	⊕⊕◯◯ LOW [a,b,d]	Not estimable	Not estimable *	2.03 (0.19,21.83)	⊕⊕◯◯ LOW
UC+WTH vs. UC	0.65 (0.35,1.18)	⊕⊕⊕◯ MODERATE [a]	Not estimable	Not estimable *	0.64 (0.35,1.18)	⊕⊕⊕◯ MODERATE
UC+SM vs. UC+GNT			2.03 (0.02,197.24)	⊕⊕◯◯ LOW [++]	2.03 (0.02,197.24)	⊕⊕◯◯ LOW
UC+WTH vs. UC+GNT			0.64 (0.01,33.70)	⊕⊕⊕◯ MODERATE [++]	0.64 (0.01,33.70)	⊕⊕⊕◯ MODERATE

UC+WTH vs. UC+SM			0.32 (0.03,3.66)	⊕⊕◯◯ LOW [++]	0.32 (0.03,3.66)	⊕⊕◯◯ LOW
**Ischemic Stroke**						
ASA vs. APX	2.70 (1.82,3.85)	⊕⊕⊕⊕ HIGH	3.28 (1.86,5.78)	⊕⊕◯◯ LOW [§]	2.85 (2.07,3.92)	⊕⊕⊕⊕ HIGH
ASA+CLP vs. APX			2.28 (1.51,3.45)	⊕⊕◯◯ LOW [++]	2.28 (1.51,3.45)	⊕⊕◯◯ LOW
CONTROL vs. APX			3.08 (1.88,5.04)	⊕⊕◯◯ LOW [++]	3.08 (1.88,5.04)	⊕⊕◯◯ LOW
DBG-110MG vs. APX			1.21 (0.89,1.63)	⊕⊕◯◯ LOW [++]	1.21 (0.89,1.63)	⊕⊕◯◯ LOW
DBG-150MG vs. APX			0.83 (0.61,1.14)	⊕⊕◯◯ LOW [++]	0.83 (0.61,1.14)	⊕⊕◯◯ LOW
EDX-30MG vs. APX			1.52 (1.18,1.98)	⊕⊕◯◯ LOW [++]	1.52 (1.18,1.98)	⊕⊕◯◯ LOW
EDX-60MG vs. APX			1.08 (0.83,1.41)	⊕⊕◯◯ LOW [++]	1.08 (0.83,1.41)	⊕⊕◯◯ LOW
RVX vs. APX			0.94 (0.71,1.24)	⊕⊕◯◯ LOW [++]	0.94 (0.71,1.24)	⊕⊕◯◯ LOW
UC vs. APX	1.10 (0.89,1.35)	⊕⊕◯◯ LOW [a,b]	0.89 (0.47,1.72)	⊕⊕◯◯ LOW [§]	1.08 (0.88,1.31)	⊕⊕◯◯ LOW
UC+WTH vs. APX			1.46 (0.72,2.98)	⊕⊕◯◯ LOW [++]	1.46 (0.72,2.98)	⊕⊕◯◯ LOW
ASA+CLP vs. ASA			0.80 (0.49,1.31)	⊕⊕◯◯ LOW [++]	0.80 (0.49,1.31)	⊕⊕◯◯ LOW
CONTROL vs. ASA			1.08 (0.62,1.89)	⊕⊕◯◯ LOW [++]	1.08 (0.62,1.89)	⊕⊕◯◯ LOW
DBG-110MG vs. ASA			0.42 (0.28,0.63)	⊕⊕◯◯ LOW [++]	0.42 (0.28,0.63)	⊕⊕◯◯ LOW
DBG-150MG vs. ASA			0.29 (0.19,0.44)	⊕⊕◯◯ LOW [++]	0.29 (0.19,0.44)	⊕⊕◯◯ LOW
EDX-30MG vs. ASA			0.53 (0.37,0.78)	⊕⊕◯◯ LOW [++]	0.53 (0.37,0.78)	⊕⊕◯◯ LOW
EDX-60MG vs. ASA			0.38 (0.26,0.55)	⊕⊕◯◯ LOW [++]	0.38 (0.26,0.55)	⊕⊕◯◯ LOW
RVX vs. ASA			0.33 (0.22,0.48)	⊕⊕◯◯ LOW [++]	0.33 (0.22,0.48)	⊕⊕◯◯ LOW
UC vs. ASA	0.32 (0.19,0.53)	⊕⊕◯◯ LOW [a,e,f]	0.41 (0.26,0.64)	⊕⊕◯◯ LOW [§]	0.38 (0.27,0.53)	⊕⊕◯◯ LOW
UC+WTH vs. ASA			0.51 (0.24,1.10)	⊕⊕◯◯ LOW [++]	0.51 (0.24,1.10)	⊕⊕◯◯ LOW
CONTROL vs. ASA+CLP			1.35 (0.76,2.41)	⊕⊕⊕◯ MODERATE [++]	1.35 (0.76,2.41)	⊕⊕⊕◯ MODERATE
DBG-110MG vs. ASA+CLP			0.53 (0.35,0.81)	⊕⊕◯◯ LOW [++]	0.53 (0.35,0.81)	⊕⊕◯◯ LOW
DBG-150MG vs. ASA+CLP			0.37 (0.24,0.57)	⊕⊕⊕◯ MODERATE [++]	0.37 (0.24,0.57)	⊕⊕⊕◯ MODERATE
EDX-30MG vs. ASA+CLP			0.67 (0.45,1.00)	⊕⊕⊕◯ MODERATE [++]	0.67 (0.45,1.00)	⊕⊕⊕◯ MODERATE
EDX-60MG vs. ASA+CLP			0.47 (0.32,0.71)	⊕⊕⊕◯ MODERATE [++]	0.47 (0.32,0.71)	⊕⊕⊕◯ MODERATE
RVX vs. ASA+CLP			0.41 (0.27,0.62)	⊕⊕◯◯ LOW [++]	0.41 (0.27,0.62)	⊕⊕◯◯ LOW
UC vs. ASA+CLP	0.47 (0.33,0.68)	⊕⊕⊕◯ MODERATE [a]	Not estimable	Not estimable *	0.47 (0.33,0.68)	⊕⊕⊕◯ MODERATE
UC+WTH vs. ASA+CLP			0.64 (0.30,1.39)	⊕⊕⊕◯ MODERATE [++]	0.64 (0.30,1.39)	⊕⊕⊕◯ MODERATE
DBG-110MG vs. CONTROL			0.39 (0.24,0.65)	⊕⊕◯◯ LOW [++]	0.39 (0.24,0.65)	⊕⊕◯◯ LOW
DBG-150MG vs. CONTROL			0.27 (0.16,0.45)	⊕⊕⊕◯ MODERATE [++]	0.27 (0.16,0.45)	⊕⊕⊕◯ MODERATE
EDX-30MG vs. CONTROL			0.49 (0.31,0.80)	⊕⊕⊕◯ MODERATE [++]	0.49 (0.31,0.80)	⊕⊕⊕◯ MODERATE

EDX-60MG vs. CONTROL			0.35 (0.22,0.57)	⊕⊕⊕◯ MODERATE [++]	0.35 (0.22,0.57)	⊕⊕⊕◯ MODERATE
RVX vs. CONTROL			0.30 (0.19,0.50)	⊕⊕◯◯ LOW [++]	0.30 (0.19,0.50)	⊕⊕◯◯ LOW
UC vs. CONTROL	0.35 (0.22,0.55)	⊕⊕⊕◯ MODERATE [a]	Not estimable	Not estimable *	0.35 (0.22,0.55)	⊕⊕⊕◯ MODERATE
UC+WTH vs. CONTROL			0.47 (0.21,1.08)	⊕⊕⊕◯ MODERATE [++]	0.47 (0.21,1.08)	⊕⊕⊕◯ MODERATE
DBG-150MG vs. DBG-110MG	0.69 (0.54,0.88)	⊕⊕⊕◯ MODERATE [a]	0.69 (0.54,0.88)	⊕⊕◯◯ LOW [§]	0.69 (0.54,0.88)	⊕⊕⊕◯ MODERATE
EDX-30MG vs. DBG-110MG			1.26 (0.96,1.67)	⊕⊕◯◯ LOW [++]	1.26 (0.96,1.67)	⊕⊕◯◯ LOW
EDX-60MG vs. DBG-110MG			0.90 (0.67,1.19)	⊕⊕◯◯ LOW [++]	0.90 (0.67,1.19)	⊕⊕◯◯ LOW
RVX vs. DBG-110MG			0.78 (0.58,1.04)	⊕⊕◯◯ LOW [++]	0.78 (0.58,1.04)	⊕⊕◯◯ LOW
UC vs. DBG-110MG	0.89 (0.71,1.11)	⊕⊕◯◯ LOW [a,b]	0.89 (0.71,1.12)	⊕⊕⊕◯ MODERATE [¶]	0.89 (0.71,1.12)	⊕⊕⊕◯ MODERATE
UC+WTH vs. DBG-110MG			1.21 (0.59,2.49)	⊕⊕◯◯ LOW [++]	1.21 (0.59,2.49)	⊕⊕◯◯ LOW
EDX-30MG vs. DBG-150MG			1.83 (1.36,2.46)	⊕⊕⊕◯ MODERATE [++]	1.83 (1.36,2.46)	⊕⊕⊕◯ MODERATE
EDX-60MG vs. DBG-150MG			1.30 (0.96,1.76)	⊕⊕⊕◯ MODERATE [++]	1.30 (0.96,1.76)	⊕⊕⊕◯ MODERATE
RVX vs. DBG-150MG			1.13 (0.83,1.53)	⊕⊕◯◯ LOW [++]	1.13 (0.83,1.53)	⊕⊕◯◯ LOW
UC vs. DBG-150MG	1.28 (1.01,1.64)	⊕⊕⊕◯ MODERATE [a]	1.29 (1.01,1.65)	⊕⊕◯◯ LOW [§]	1.29 (1.01,1.65)	⊕⊕⊕◯ MODERATE
UC+WTH vs. DBG-150MG			1.75 (0.85,3.63)	⊕⊕⊕◯ MODERATE [++]	1.75 (0.85,3.63)	⊕⊕⊕◯ MODERATE
EDX-60MG vs. EDX-30MG	0.71 (0.60,0.83)	⊕⊕⊕⊕ HIGH	0.71 (0.60,0.83)	⊕⊕⊕◯ MODERATE [¶]	0.71 (0.60,0.83)	⊕⊕⊕⊕ HIGH
RVX vs. EDX-30MG			0.62 (0.48,0.79)	⊕⊕◯◯ LOW [++]	0.62 (0.48,0.79)	⊕⊕◯◯ LOW
UC vs. EDX-30MG	0.70 (0.60,0.83)	⊕⊕⊕⊕ HIGH	0.71 (0.60,0.83)	⊕⊕⊕◯ MODERATE [¶]	0.71 (0.60,0.83)	⊕⊕⊕⊕ HIGH
UC+WTH vs. EDX-30MG			0.96 (0.48,1.94)	⊕⊕⊕◯ MODERATE [++]	0.96 (0.48,1.94)	⊕⊕⊕◯ MODERATE
RVX vs. EDX-60MG			0.87 (0.67,1.13)	⊕⊕◯◯ LOW [++]	0.87 (0.67,1.13)	⊕⊕◯◯ LOW
UC vs. EDX-60MG	1.00 (0.83,1.19)	⊕⊕⊕◯ MODERATE [b]	1.00 (0.83,1.19)	⊕⊕⊕⊕ HIGH [¶]	1.00 (0.83,1.19)	⊕⊕⊕⊕ HIGH
UC+WTH vs. EDX-60MG			1.35 (0.67,2.74)	⊕⊕⊕◯ MODERATE [++]	1.35 (0.67,2.74)	⊕⊕⊕◯ MODERATE
UC vs. RVX	1.15 (0.95,1.39)	⊕⊕◯◯ LOW [a,b]	Not estimable	Not estimable *	1.15 (0.95,1.39)	⊕⊕◯◯ LOW
UC+WTH vs. RVX			1.56 (0.77,3.17)	⊕⊕◯◯ LOW [++]	1.56 (0.77,3.17)	⊕⊕◯◯ LOW
UC+WTH vs. UC	1.39 (0.70,2.70)	⊕⊕⊕◯ MODERATE [a]	Not estimable	Not estimable *	1.36 (0.69,2.69)	⊕⊕⊕◯ MODERATE
**Clinically relevant non-major bleeding**						
ASA vs. APX	0.88 (0.66,1.18)	⊕⊕⊕⊕ HIGH	Not estimable	Not estimable *	0.88 (0.66,1.17)	⊕⊕⊕⊕ HIGH
CONTROL vs. APX			0.49 (0.05,4.65)	⊕⊕◯◯ LOW [++]	0.49 (0.05,4.65)	⊕⊕◯◯ LOW
DBG-110MG vs. APX			0.74 (0.16,3.46)	⊕⊕◯◯ LOW [++]	0.74 (0.16,3.46)	⊕⊕◯◯ LOW
DBG-150MG vs. APX			1.65 (0.73,3.72)	⊕⊕⊕◯ MODERATE [++]	1.65 (0.73,3.72)	⊕⊕⊕◯ MODERATE
EDX-30MG vs. APX			1.02 (0.86,1.20)	⊕⊕⊕◯ MODERATE [++]	1.02 (0.86,1.20)	⊕⊕⊕◯ MODERATE
EDX-60MG vs. APX			1.28 (1.09,1.51)	⊕⊕⊕◯ MODERATE [++]	1.28 (1.09,1.51)	⊕⊕⊕◯ MODERATE

RVX vs. APX			1.52 (1.29,1.79)	⊕⊕⊕◯ MODERATE [++]	1.52 (1.29,1.79)	⊕⊕⊕◯ MODERATE
UC vs. APX	1.47 (1.27,1.69)	⊕⊕⊕◯ MODERATE [a]	Not estimable	Not estimable *	1.46 (1.26,1.70)	⊕⊕⊕◯ MODERATE
CONTROL vs. ASA			0.55 (0.06,5.38)	⊕⊕◯◯ LOW [++]	0.55 (0.06,5.38)	⊕⊕◯◯ LOW
DBG-110MG vs. ASA			0.84 (0.18,4.03)	⊕⊕◯◯ LOW [++]	0.84 (0.18,4.03)	⊕⊕◯◯ LOW
DBG-150MG vs. ASA			1.88 (0.79,4.45)	⊕⊕⊕◯ MODERATE [++]	1.88 (0.79,4.45)	⊕⊕⊕◯ MODERATE
EDX-30MG vs. ASA			1.16 (0.83,1.61)	⊕⊕⊕◯ MODERATE [++]	1.16 (0.83,1.61)	⊕⊕⊕◯ MODERATE
EDX-60MG vs. ASA			1.45 (1.04,2.02)	⊕⊕⊕◯ MODERATE [++]	1.45 (1.04,2.02)	⊕⊕⊕◯ MODERATE
RVX vs. ASA			1.73 (1.24,2.41)	⊕⊕⊕◯ MODERATE [++]	1.73 (1.24,2.41)	⊕⊕⊕◯ MODERATE
UC vs. ASA			1.66 (1.20,2.30)	⊕⊕⊕◯ MODERATE [++]	1.66 (1.20,2.30)	⊕⊕⊕◯ MODERATE
DBG-110MG vs. CONTROL			1.53 (0.10,23.33)	⊕⊕◯◯ LOW [++]	1.53 (0.10,23.33)	⊕⊕◯◯ LOW
DBG-150MG vs. CONTROL			3.40 (0.31,37.22)	⊕⊕◯◯ LOW [++]	3.40 (0.31,37.22)	⊕⊕◯◯ LOW
EDX-30MG vs. CONTROL			2.10 (0.22,20.01)	⊕⊕◯◯ LOW [++]	2.10 (0.22,20.01)	⊕⊕◯◯ LOW
EDX-60MG vs. CONTROL			2.63 (0.28,25.15)	⊕⊕◯◯ LOW [++]	2.63 (0.28,25.15)	⊕⊕◯◯ LOW
RVX vs. CONTROL			3.13 (0.33,29.88)	⊕⊕◯◯ LOW [++]	3.13 (0.33,29.88)	⊕⊕◯◯ LOW
UC vs. CONTROL	3.03 (0.32,25.00)	⊕⊕◯◯ LOW [a,c,d]	Not estimable	Not estimable *	3.01 (0.32,28.74)	⊕⊕◯◯ LOW
DBG-150MG vs. DBG-110MG	2.00 (0.4,10.0)	⊕⊕◯◯ LOW [a,d]	4.63 (0.20,109.95)	⊕⊕◯◯ LOW [§]	2.23 (0.48,10.30)	⊕⊕◯◯ LOW
EDX-30MG vs. DBG-110MG			1.37 (0.30,6.35)	⊕⊕◯◯ LOW [++]	1.37 (0.30,6.35)	⊕⊕◯◯ LOW
EDX-60MG vs. DBG-110MG			1.72 (0.37,7.98)	⊕⊕◯◯ LOW [++]	1.72 (0.37,7.98)	⊕⊕◯◯ LOW
RVX vs. DBG-110MG			2.05 (0.44,9.48)	⊕⊕◯◯ LOW [++]	2.05 (0.44,9.48)	⊕⊕◯◯ LOW
UC vs. DBG-110MG	2.22 (0.45,11.10)	⊕⊕◯◯ LOW [a,d]	0.95 (0.04,22.54)	⊕⊕◯◯ LOW [§]	1.97 (0.43,9.12)	⊕⊕◯◯ LOW
EDX-30MG vs. DBG-150MG			0.62 (0.28,1.37)	⊕⊕⊕◯ MODERATE [++]	0.62 (0.28,1.37)	⊕⊕⊕◯ MODERATE
EDX-60MG vs. DBG-150MG			0.77 (0.35,1.73)	⊕⊕⊕◯ MODERATE [++]	0.77 (0.35,1.73)	⊕⊕⊕◯ MODERATE
RVX vs. DBG-150MG			0.92 (0.41,2.05)	⊕⊕⊕◯ MODERATE [++]	0.92 (0.41,2.05)	⊕⊕⊕◯ MODERATE
UC vs. DBG-150MG	0.88 (0.40,1.92)	⊕⊕⊕◯ MODERATE [a]	0.89 (0.40,1.97)	⊕⊕◯◯ LOW [§]	0.89 (0.40,1.97)	⊕⊕⊕◯ MODERATE
EDX-60MG vs. EDX-30MG	1.27 (1.16,1.37)	⊕⊕⊕◯ MODERATE [a]	1.26 (1.16,1.36)	⊕⊕⊕◯ MODERATE [¶]	1.26 (1.16,1.36)	⊕⊕⊕◯ MODERATE
RVX vs. EDX-30MG			1.49 (1.35,1.66)	⊕⊕⊕◯ MODERATE [++]	1.49 (1.35,1.66)	⊕⊕⊕◯ MODERATE
UC vs. EDX-30MG	1.45 (1.33,1.56)	⊕⊕⊕◯ MODERATE [a]	1.44 (1.33,1.55)	⊕⊕⊕◯ MODERATE [¶]	1.44 (1.33,1.55)	⊕⊕⊕◯ MODERATE
RVX vs. EDX-60MG			1.19 (1.08,1.31)	⊕⊕⊕◯ MODERATE [++]	1.19 (1.08,1.31)	⊕⊕⊕◯ MODERATE
UC vs. EDX-60MG	1.14 (1.06,1.22)	⊕⊕⊕◯ MODERATE [a]	1.14 (1.07,1.23)	⊕⊕⊕◯ MODERATE [¶]	1.14 (1.07,1.23)	⊕⊕⊕◯ MODERATE
UC vs. RVX	0.96 (0.89,1.03)	⊕⊕⊕◯ MODERATE [a]	Not estimable	Not estimable *	0.96 (0.90,1.03)	⊕⊕⊕◯ MODERATE
**Intracranial bleeding**						
ASA vs. APX	1.19 (0.53,2.63)	⊕⊕⊕◯ MODERATE [b,c]	1.91 (0.70,5.24)	⊕◯◯◯ VERY LOW [§]	1.47 (0.76,2.83)	⊕⊕⊕◯ MODERATE

ASA+CLP vs. APX			1.17 (0.46,2.97)	⊕⊕◯◯ LOW [++]	1.17 (0.46,2.97)	⊕⊕◯◯ LOW
CONTROL vs. APX			1.29 (0.41,4.08)	⊕⊕◯◯ LOW [++]	1.29 (0.41,4.08)	⊕⊕◯◯ LOW
DBG-110MG vs. APX			0.70 (0.34,1.45)	⊕⊕◯◯ LOW [++]	0.70 (0.34,1.45)	⊕⊕◯◯ LOW
DBG-150MG vs. APX			0.93 (0.46,1.86)	⊕⊕◯◯ LOW [++]	0.93 (0.46,1.86)	⊕⊕◯◯ LOW
EDX-30MG vs. APX			0.71 (0.36,1.39)	⊕⊕◯◯ LOW [++]	0.71 (0.36,1.39)	⊕⊕◯◯ LOW
EDX-60MG vs. APX			1.09 (0.57,2.08)	⊕⊕◯◯ LOW [++]	1.09 (0.57,2.08)	⊕⊕◯◯ LOW
RVX vs. APX			1.72 (0.96,3.07)	⊕⊕◯◯ LOW [++]	1.72 (0.96,3.07)	⊕⊕◯◯ LOW
UC vs. APX	2.38 (1.72,3.23)	⊕⊕◯◯ LOW [a,c]	1.50 (0.43,5.22)	⊕◯◯◯ VERY LOW [§]	2.26 (1.43,3.55)	⊕⊕◯◯ LOW
UC+SM vs. APX			2.25 (0.29,17.30)	⊕◯◯◯ VERY LOW [++]	2.25 (0.29,17.30)	⊕◯◯◯ VERY LOW
UC+ST vs. APX			3.09 (1.05,9.07)	⊕◯◯◯ VERY LOW [++]	3.09 (1.05,9.07)	⊕◯◯◯ VERY LOW
UC+WTH vs. APX			0.50 (0.13,1.85)	⊕⊕◯◯ LOW [++]	0.50 (0.13,1.85)	⊕⊕◯◯ LOW
ASA+CLP vs. ASA			0.80 (0.28,2.27)	⊕⊕◯◯ LOW [++]	0.80 (0.28,2.27)	⊕⊕◯◯ LOW
CONTROL vs. ASA	0.48 (0.09,2.60)	⊕⊕◯◯ LOW [a,b,d]	1.40 (0.31,6.38)	⊕◯◯◯ VERY LOW [§]	0.88 (0.28,2.75)	⊕⊕◯◯ LOW
DBG-110MG vs. ASA			0.48 (0.20,1.14)	⊕◯◯◯ VERY LOW [++]	0.48 (0.20,1.14)	⊕◯◯◯ VERY LOW
DBG-150MG vs. ASA			0.63 (0.27,1.47)	⊕◯◯◯ VERY LOW [++]	0.63 (0.27,1.47)	⊕◯◯◯ VERY LOW
EDX-30MG vs. ASA			0.48 (0.21,1.10)	⊕◯◯◯ VERY LOW [++]	0.48 (0.21,1.10)	⊕◯◯◯ VERY LOW
EDX-60MG vs. ASA			0.74 (0.33,1.65)	⊕◯◯◯ VERY LOW [++]	0.74 (0.33,1.65)	⊕◯◯◯ VERY LOW
RVX vs. ASA			1.17 (0.55,2.48)	⊕◯◯◯ VERY LOW [++]	1.17 (0.55,2.48)	⊕◯◯◯ VERY LOW
UC vs. ASA	1.49 (0.60,3.70)	⊕◯◯◯ VERY LOW [a,b,c,f,g]	1.61 (0.62,4.19)	⊕⊕◯◯ LOW [§]	1.54 (0.80,2.96)	⊕⊕◯◯ LOW
UC+SM vs. ASA			1.53 (0.19,12.45)	⊕◯◯◯ VERY LOW [++]	1.53 (0.19,12.45)	⊕◯◯◯ VERY LOW
UC+ST vs. ASA			2.10 (0.65,6.83)	⊕◯◯◯ VERY LOW [++]	2.10 (0.65,6.83)	⊕◯◯◯ VERY LOW
UC+WTH vs. ASA			0.34 (0.08,1.37)	⊕◯◯◯ VERY LOW [++]	0.34 (0.08,1.37)	⊕◯◯◯ VERY LOW
CONTROL vs. ASA+CLP			1.10 (0.28,4.32)	⊕⊕◯◯ LOW [++]	1.10 (0.28,4.32)	⊕⊕◯◯ LOW
DBG-110MG vs. ASA+CLP			0.60 (0.22,1.62)	⊕⊕◯◯ LOW [++]	0.60 (0.22,1.62)	⊕⊕◯◯ LOW
DBG-150MG vs. ASA+CLP			0.79 (0.30,2.09)	⊕⊕◯◯ LOW [++]	0.79 (0.30,2.09)	⊕⊕◯◯ LOW
EDX-30MG vs. ASA+CLP			0.61 (0.23,1.58)	⊕⊕◯◯ LOW [++]	0.61 (0.23,1.58)	⊕⊕◯◯ LOW
EDX-60MG vs. ASA+CLP			0.93 (0.36,2.39)	⊕⊕◯◯ LOW [++]	0.93 (0.36,2.39)	⊕⊕◯◯ LOW
RVX vs. ASA+CLP			1.47 (0.60,3.57)	⊕⊕◯◯ LOW [++]	1.47 (0.60,3.57)	⊕⊕◯◯ LOW
UC vs. ASA+CLP	1.92 (0.93,4.00)	⊕⊕◯◯ LOW [a,b,c]	1.93 (0.85,4.36)	⊕◯◯◯ VERY LOW [§]	1.93 (0.85,4.36)	⊕⊕◯◯ LOW
UC+SM vs. ASA+CLP			1.92 (0.22,16.51)	⊕◯◯◯ VERY LOW [++]	1.92 (0.22,16.51)	⊕◯◯◯ VERY LOW
UC+ST vs. ASA+CLP			2.64 (0.74,9.43)	⊕◯◯◯ VERY LOW [++]	2.64 (0.74,9.43)	⊕◯◯◯ VERY LOW

UC+WTH vs. ASA+CLP			0.43 (0.10,1.87)	⊕⊕◯◯ LOW [++]	0.43 (0.10,1.87)	⊕⊕◯◯ LOW
DBG-110MG vs. CONTROL			0.54 (0.16,1.87)	⊕⊕◯◯ LOW [++]	0.54 (0.16,1.87)	⊕⊕◯◯ LOW
DBG-150MG vs. CONTROL			0.72 (0.21,2.43)	⊕⊕◯◯ LOW [++]	0.72 (0.21,2.43)	⊕⊕◯◯ LOW
EDX-30MG vs. CONTROL			0.55 (0.17,1.84)	⊕⊕◯◯ LOW [++]	0.55 (0.17,1.84)	⊕⊕◯◯ LOW
EDX-60MG vs. CONTROL			0.84 (0.26,2.78)	⊕⊕◯◯ LOW [++]	0.84 (0.26,2.78)	⊕⊕◯◯ LOW
RVX vs. CONTROL			1.33 (0.42,4.23)	⊕⊕◯◯ LOW [++]	1.33 (0.42,4.23)	⊕⊕◯◯ LOW
UC vs. CONTROL	1.20 (0.38,4.20)	⊕⊕◯◯ LOW [a,b,c]	3.53 (0.55,22.67)	⊕◯◯◯ VERY LOW [§]	1.75 (0.59,5.25)	⊕⊕◯◯ LOW
UC+SM vs. CONTROL			1.75 (0.18,16.93)	⊕◯◯◯ VERY LOW [++]	1.75 (0.18,16.93)	⊕◯◯◯ VERY LOW
UC+ST vs. CONTROL			2.40 (0.55,10.42)	⊕◯◯◯ VERY LOW [++]	2.40 (0.55,10.42)	⊕◯◯◯ VERY LOW
UC+WTH vs. CONTROL			0.39 (0.07,2.01)	⊕⊕◯◯ LOW [++]	0.39 (0.07,2.01)	⊕⊕◯◯ LOW
DBG-150MG vs. DBG-110MG	1.32 (0.80,2.17)	⊕⊕◯◯ LOW [a,b,c]	1.32 (0.71,2.45)	⊕⊕◯◯ LOW [§]	1.32 (0.71,2.45)	⊕⊕◯◯ LOW
EDX-30MG vs. DBG-110MG			1.01 (0.48,2.16)	⊕⊕◯◯ LOW [++]	1.01 (0.48,2.16)	⊕⊕◯◯ LOW
EDX-60MG vs. DBG-110MG			1.55 (0.74,3.25)	⊕⊕◯◯ LOW [++]	1.55 (0.74,3.25)	⊕⊕◯◯ LOW
RVX vs. DBG-110MG			2.45 (1.26,4.77)	⊕⊕◯◯ LOW [++]	2.45 (1.26,4.77)	⊕⊕◯◯ LOW
UC vs. DBG-110MG	3.23 (2.08,5.00)	⊕⊕◯◯ LOW [a,c]	3.22 (1.83,5.66)	⊕⊕◯◯ LOW [§]	3.22 (1.83,5.66)	⊕⊕◯◯ LOW
UC+SM vs. DBG-110MG			3.21 (0.41,25.37)	⊕◯◯◯ VERY LOW [++]	3.21 (0.41,25.37)	⊕◯◯◯ VERY LOW
UC+ST vs. DBG-110MG			4.40 (1.42,13.63)	⊕◯◯◯ VERY LOW [++]	4.40 (1.42,13.63)	⊕◯◯◯ VERY LOW
UC+WTH vs. DBG-110MG			0.71 (0.18,2.76)	⊕⊕◯◯ LOW [++]	0.71 (0.18,2.76)	⊕⊕◯◯ LOW
EDX-30MG vs. DBG-150MG			0.77 (0.37,1.60)	⊕⊕◯◯ LOW [++]	0.77 (0.37,1.60)	⊕⊕◯◯ LOW
EDX-60MG vs. DBG-150MG			1.17 (0.57,2.40)	⊕⊕⊕◯ MODERATE [++]	1.17 (0.57,2.40)	⊕⊕⊕◯ MODERATE
RVX vs. DBG-150MG			1.85 (0.98,3.52)	⊕⊕⊕◯ MODERATE [++]	1.85 (0.98,3.52)	⊕⊕⊕◯ MODERATE
UC vs. DBG-150MG	2.43 (1.67,3.57)	⊕⊕⊕◯ MODERATE [a]	2.44 (1.43,4.15)	⊕⊕◯◯ LOW [§]	2.44 (1.43,4.15)	⊕⊕⊕◯ MODERATE
UC+SM vs. DBG-150MG			2.43 (0.31,19.06)	⊕◯◯◯ VERY LOW [++]	2.43 (0.31,19.06)	⊕◯◯◯ VERY LOW
UC+ST vs. DBG-150MG			3.33 (1.09,10.16)	⊕◯◯◯ VERY LOW [++]	3.33 (1.09,10.16)	⊕◯◯◯ VERY LOW
UC+WTH vs. DBG-150MG			0.54 (0.14,2.06)	⊕⊕⊕◯ MODERATE [++]	0.54 (0.14,2.06)	⊕⊕⊕◯ MODERATE
EDX-60MG vs. EDX-30MG	1.52 (1.02,2.22)	⊕⊕⊕◯ MODERATE [a]	1.53 (0.90,2.61)	⊕⊕◯◯ LOW [§]	1.53 (0.90,2.61)	⊕⊕⊕◯ MODERATE
RVX vs. EDX-30MG			2.42 (1.30,4.49)	⊕⊕◯◯ LOW [++]	2.42 (1.30,4.49)	⊕⊕◯◯ LOW
UC vs. EDX-30MG	3.23 (2.27,4.54)	⊕⊕◯◯ LOW [a,c]	3.18 (1.92,5.26)	⊕⊕⊕◯ MODERATE [¶]	3.18 (1.92,5.26)	⊕⊕⊕◯ MODERATE
UC+SM vs. EDX-30MG			3.17 (0.41,24.65)	⊕◯◯◯ VERY LOW [++]	3.17 (0.41,24.65)	⊕◯◯◯ VERY LOW
UC+ST vs. EDX-30MG			4.35 (1.45,13.06)	⊕◯◯◯ VERY LOW [++]	4.35 (1.45,13.06)	⊕◯◯◯ VERY LOW

UC+WTH vs. EDX-30MG			0.70 (0.19,2.65)	⊕⊕◯◯ LOW [++]	0.70 (0.19,2.65)	⊕⊕◯◯ LOW
RVX vs. EDX-60MG			1.58 (0.86,2.89)	⊕⊕⊕◯ MODERATE [++]	1.58 (0.86,2.89)	⊕⊕⊕◯ MODERATE
UC vs. EDX-60MG	2.13 (1.56,2.86)	⊕⊕⊕◯ MODERATE [a]	2.08 (1.29,3.35)	⊕⊕◯◯ LOW [§]	2.08 (1.29,3.35)	⊕⊕◯◯ LOW
UC+SM vs. EDX-60MG			2.07 (0.27,16.01)	⊕◯◯◯ VERY LOW [++]	2.07 (0.27,16.01)	⊕◯◯◯ VERY LOW
UC+ST vs. EDX-60MG			2.84 (0.96,8.44)	⊕◯◯◯ VERY LOW [++]	2.84 (0.96,8.44)	⊕◯◯◯ VERY LOW
UC+WTH vs. EDX-60MG			0.46 (0.12,1.71)	⊕⊕⊕◯ MODERATE [++]	0.46 (0.12,1.71)	⊕⊕⊕◯ MODERATE
UC vs. RVX	1.37 (1.06,1.79)	⊕⊕⊕◯ MODERATE [a]	Not estimable	Not estimable *	1.31 (0.92,1.87)	⊕⊕⊕◯ MODERATE
UC+SM vs. RVX			1.31 (0.17,9.88)	⊕◯◯◯ VERY LOW [++]	1.31 (0.17,9.88)	⊕◯◯◯ VERY LOW
UC+ST vs. RVX			1.80 (0.63,5.09)	⊕◯◯◯ VERY LOW [++]	1.80 (0.63,5.09)	⊕◯◯◯ VERY LOW
UC+WTH vs. RVX			0.29 (0.08,1.05)	⊕⊕⊕◯ MODERATE [++]	0.29 (0.08,1.05)	⊕⊕⊕◯ MODERATE
UC+SM vs. UC	1.00 (0.14,7.14)	⊕◯◯◯ VERY LOW [a,b,c,d,h]	Not estimable	Not estimable *	1.00 (0.14,7.29)	⊕◯◯◯ VERY LOW
UC+ST vs. UC	1.37 (0.55,3.33)	⊕◯◯◯ VERY LOW [a,b,c,h]	Not estimable	Not estimable *	1.37 (0.51,3.64)	⊕◯◯◯ VERY LOW
UC+WTH vs. UC	0.22 (0.07,0.68)	⊕⊕⊕◯ MODERATE [c]	Not estimable	Not estimable *	0.22 (0.06,0.76)	⊕⊕⊕◯ MODERATE
UC+ST vs. UC+SM			1.37 (0.15,12.58)	⊕◯◯◯ VERY LOW [++]	1.37 (0.15,12.58)	⊕◯◯◯ VERY LOW
UC+WTH vs. UC+SM			0.22 (0.02,2.30)	⊕◯◯◯ VERY LOW [++]	0.22 (0.02,2.30)	⊕◯◯◯ VERY LOW
UC+WTH vs. UC+ST			0.16 (0.03,0.78)	⊕◯◯◯ VERY LOW [++]	0.16 (0.03,0.78)	⊕◯◯◯ VERY LOW
**Gastrointestinal bleeding**						
ASA vs. APX	1.18 (0.54,2.50)	⊕⊕◯◯ LOW [c,d]	5.55 (0.11,275.93)	⊕⊕⊕◯ MODERATE [¶]	0.78 (0.16,3.83)	⊕⊕⊕◯ MODERATE
CONTROL vs. APX			0.38 (0.02,5.66)	⊕⊕◯◯ LOW [++]	0.38 (0.02,5.66)	⊕⊕◯◯ LOW
DBG-110MG vs. APX			1.79 (0.19,17.10)	⊕⊕⊕◯ MODERATE [++]	1.79 (0.19,17.10)	⊕⊕⊕◯ MODERATE
DBG-150MG vs. APX			2.42 (0.25,23.12)	⊕⊕⊕◯ MODERATE [++]	2.42 (0.25,23.12)	⊕⊕⊕◯ MODERATE
EDX-30MG vs. APX			1.09 (0.11,10.44)	⊕⊕⊕◯ MODERATE [++]	1.09 (0.11,10.44)	⊕⊕⊕◯ MODERATE
EDX-60MG vs. APX			1.97 (0.21,18.72)	⊕⊕⊕◯ MODERATE [++]	1.97 (0.21,18.72)	⊕⊕⊕◯ MODERATE
RVX vs. APX			2.20 (0.30,16.04)	⊕⊕◯◯ LOW [++]	2.20 (0.30,16.04)	⊕⊕◯◯ LOW
UC vs. APX	1.14 (0.88,1.47)	⊕⊕⊕◯ MODERATE [d]	5.77 (0.12,286.98)	⊕⊕◯◯ LOW [§]	1.61 (0.35,7.41)	⊕⊕⊕◯ MODERATE
UC+GNT vs. APX			1.19 (0.11,12.68)	⊕⊕◯◯ LOW [++]	1.19 (0.11,12.68)	⊕⊕◯◯ LOW
CONTROL vs. ASA			0.48 (0.03,9.22)	⊕⊕◯◯ LOW [++]	0.48 (0.03,9.22)	⊕⊕◯◯ LOW
DBG-110MG vs. ASA			2.30 (0.18,29.06)	⊕⊕⊕◯ MODERATE [++]	2.30 (0.18,29.06)	⊕⊕⊕◯ MODERATE
DBG-150MG vs. ASA			3.11 (0.25,39.30)	⊕⊕⊕◯ MODERATE [++]	3.11 (0.25,39.30)	⊕⊕⊕◯ MODERATE
EDX-30MG vs. ASA			1.41 (0.11,17.75)	⊕⊕⊕◯ MODERATE [++]	1.41 (0.11,17.75)	⊕⊕⊕◯ MODERATE
EDX-60MG vs. ASA			2.53 (0.20,31.83)	⊕⊕⊕◯ MODERATE [++]	2.53 (0.20,31.83)	⊕⊕⊕◯ MODERATE

RVX vs. ASA			2.83 (0.27,29.15)	⊕⊕◯◯ LOW [++]	2.83 (0.27,29.15)	⊕⊕◯◯ LOW
UC vs. ASA	5.00 (0.61,50.00)	⊕⊕⊕◯ MODERATE [c,d]	0.97 (0.02,48.21)	⊕⊕◯◯ LOW [§]	2.07 (0.30,14.05)	⊕⊕⊕◯ MODERATE
UC+GNT vs. ASA			1.52 (0.11,21.29)	⊕⊕◯◯ LOW [++]	1.52 (0.11,21.29)	⊕⊕◯◯ LOW
DBG-110MG vs. CONTROL			4.75 (0.29,77.51)	⊕⊕◯◯ LOW [++]	4.75 (0.29,77.51)	⊕⊕◯◯ LOW
DBG-150MG vs. CONTROL			6.43 (0.39,104.86)	⊕⊕◯◯ LOW [++]	6.43 (0.39,104.86)	⊕⊕◯◯ LOW
EDX-30MG vs. CONTROL			2.91 (0.18,47.35)	⊕⊕◯◯ LOW [++]	2.91 (0.18,47.35)	⊕⊕◯◯ LOW
EDX-60MG vs. CONTROL			5.23 (0.32,84.94)	⊕⊕◯◯ LOW [++]	5.23 (0.32,84.94)	⊕⊕◯◯ LOW
RVX vs. CONTROL			5.85 (0.47,73.58)	⊕⊕◯◯ LOW [++]	5.85 (0.47,73.58)	⊕⊕◯◯ LOW
UC vs. CONTROL	4.35 (0.93,20.00)	⊕⊕◯◯ LOW [b,c,d]	Not Estimable	Not estimable *	4.28 (0.45,40.27)	⊕⊕◯◯ LOW
UC+GNT vs. CONTROL			3.15 (0.18,56.31)	⊕⊕◯◯ LOW [++]	3.15 (0.18,56.31)	⊕⊕◯◯ LOW
DBG-150MG vs. DBG-110MG	1.35 (1.09,1.69)	⊕⊕⊕◯ MODERATE [a]	1.35 (0.26,7.14)	⊕⊕⊕◯ MODERATE [¶]	1.35 (0.26,7.14)	⊕⊕⊕◯ MODERATE
EDX-30MG vs. DBG-110MG			0.61 (0.06,6.43)	⊕⊕⊕◯ MODERATE [++]	0.61 (0.06,6.43)	⊕⊕⊕◯ MODERATE
EDX-60MG vs. DBG-110MG			1.10 (0.10,11.54)	⊕⊕⊕◯ MODERATE [++]	1.10 (0.10,11.54)	⊕⊕⊕◯ MODERATE
RVX vs. DBG-110MG			1.23 (0.16,9.46)	⊕⊕◯◯ LOW [++]	1.23 (0.16,9.46)	⊕⊕◯◯ LOW
UC vs. DBG-110MG	0.90 (0.70,1.15)	⊕⊕⊕◯ MODERATE [a]	0.90 (0.17,4.76)	⊕⊕⊕◯ MODERATE [¶]	0.90 (0.17,4.76)	⊕⊕⊕◯ MODERATE
UC+GNT vs. DBG-110MG			0.66 (0.06,7.78)	⊕⊕◯◯ LOW [++]	0.66 (0.06,7.78)	⊕⊕◯◯ LOW
EDX-30MG vs. DBG-150MG			0.45 (0.04,4.74)	⊕⊕⊕◯ MODERATE [++]	0.45 (0.04,4.74)	⊕⊕⊕◯ MODERATE
EDX-60MG vs. DBG-150MG			0.81 (0.08,8.50)	⊕⊕⊕◯ MODERATE [++]	0.81 (0.08,8.50)	⊕⊕⊕◯ MODERATE
RVX vs. DBG-150MG			0.91 (0.12,6.97)	⊕⊕◯◯ LOW [++]	0.91 (0.12,6.97)	⊕⊕◯◯ LOW
UC vs. DBG-150MG	0.67 (0.53,0.83)	⊕⊕⊕◯ MODERATE [a]	0.67 (0.13,3.51)	⊕⊕⊕◯ MODERATE [¶]	0.67 (0.13,3.51)	⊕⊕⊕◯ MODERATE
UC+GNT vs. DBG-150MG			0.49 (0.04,5.73)	⊕⊕◯◯ LOW [++]	0.49 (0.04,5.73)	⊕⊕◯◯ LOW
EDX-60MG vs. EDX-30MG	1.79 (1.45,2.22)	⊕⊕⊕⊕ HIGH	1.80 (0.34,9.46)	⊕⊕⊕⊕ HIGH [¶]	1.80 (0.34,9.46)	⊕⊕⊕⊕ HIGH
RVX vs. EDX-30MG			2.01 (0.26,15.42)	⊕⊕◯◯ LOW [++]	2.01 (0.26,15.42)	⊕⊕◯◯ LOW
UC vs. EDX-30MG	1.47 (1.18,1.85)	⊕⊕⊕⊕ HIGH	1.47 (0.28,7.76)	⊕⊕⊕⊕ HIGH [¶]	1.47 (0.28,7.76)	⊕⊕⊕⊕ HIGH
UC+GNT vs. EDX-30MG			1.08 (0.09,12.68)	⊕⊕◯◯ LOW [++]	1.08 (0.09,12.68)	⊕⊕◯◯ LOW
RVX vs. EDX-60MG			1.12 (0.15,8.55)	⊕⊕◯◯ LOW [++]	1.12 (0.15,8.55)	⊕⊕◯◯ LOW
UC vs. EDX-60MG	0.82 (0.68,0.99)	⊕⊕⊕⊕ HIGH	0.82 (0.16,4.30)	⊕⊕⊕⊕ HIGH [¶]	0.82 (0.16,4.30)	⊕⊕⊕⊕ HIGH
UC+GNT vs. EDX-60MG			0.60 (0.05,7.03)	⊕⊕◯◯ LOW [++]	0.60 (0.05,7.03)	⊕⊕◯◯ LOW
UC vs. RVX	0.70 (0.57,0.85)	⊕⊕◯◯ LOW [a,e]	Not estimable	Not estimable *	0.73 (0.23,2.37)	⊕⊕◯◯ LOW
UC+GNT vs. RVX			0.54 (0.06,4.67)	⊕⊕◯◯ LOW [++]	0.54 (0.06,4.67)	⊕⊕◯◯ LOW
UC+GNT vs. UC	0.74 (0.35,1.56)	⊕⊕◯◯ LOW [a,h]	Not estimable	Not estimable *	0.74 (0.12,4.51)	⊕⊕◯◯ LOW
**Myocardial Infarction**						

ASA vs. APX	1.18 (0.68,2.00)	⊕⊕⊕◯ MODERATE [c]	1.20 (0.63,2.31)	⊕⊕◯◯ LOW [§]	1.19 (0.78,1.80)	⊕⊕⊕◯ MODERATE
ASA+CLP vs. APX			1.76 (0.98,3.16)	⊕⊕◯◯ LOW [++]	1.76 (0.98,3.16)	⊕⊕◯◯ LOW
CONTROL vs. APX			1.13 (0.16,8.11)	⊕⊕◯◯ LOW [++]	1.13 (0.16,8.11)	⊕⊕◯◯ LOW
DBG-110MG vs. APX			1.55 (1.02,2.36)	⊕⊕⊕◯ MODERATE [++]	1.55 (1.02,2.36)	⊕⊕⊕◯ MODERATE
DBG-150MG vs. APX			1.59 (1.05,2.41)	⊕⊕⊕◯ MODERATE [++]	1.59 (1.05,2.41)	⊕⊕⊕◯ MODERATE
EDX-30MG vs. APX			1.37 (0.97,1.94)	⊕⊕⊕◯ MODERATE [++]	1.37 (0.97,1.94)	⊕⊕⊕◯ MODERATE
EDX-60MG vs. APX			1.08 (0.76,1.54)	⊕⊕⊕◯ MODERATE [++]	1.08 (0.76,1.54)	⊕⊕⊕◯ MODERATE
RVX vs. APX			0.93 (0.64,1.35)	⊕⊕⊕◯ MODERATE [++]	0.93 (0.64,1.35)	⊕⊕⊕◯ MODERATE
UC vs. APX	1.14 (0.85,1.51)	⊕⊕⊕⊕ HIGH [b]	1.11 (0.50,2.48)	⊕⊕◯◯ LOW [§]	1.14 (0.87,1.48)	⊕⊕⊕⊕ HIGH
UC+ST vs. APX			1.70 (0.89,3.25)	⊕⊕◯◯ LOW [++]	1.70 (0.89,3.25)	⊕⊕◯◯ LOW
ASA+CLP vs. ASA			1.49 (0.76,2.91)	⊕⊕◯◯ LOW [++]	1.49 (0.76,2.91)	⊕⊕◯◯ LOW
CONTROL vs. ASA			0.95 (0.13,7.03)	⊕⊕◯◯ LOW [++]	0.95 (0.13,7.03)	⊕⊕◯◯ LOW
DBG-110MG vs. ASA			1.31 (0.77,2.23)	⊕⊕◯◯ LOW [++]	1.31 (0.77,2.23)	⊕⊕◯◯ LOW
DBG-150MG vs. ASA			1.34 (0.79,2.28)	⊕⊕◯◯ LOW [++]	1.34 (0.79,2.28)	⊕⊕◯◯ LOW
EDX-30MG vs. ASA			1.16 (0.72,1.87)	⊕⊕◯◯ LOW [++]	1.16 (0.72,1.87)	⊕⊕◯◯ LOW
EDX-60MG vs. ASA			0.91 (0.56,1.48)	⊕⊕◯◯ LOW [++]	0.91 (0.56,1.48)	⊕⊕◯◯ LOW
RVX vs. ASA			0.79 (0.48,1.29)	⊕⊕◯◯ LOW [++]	0.79 (0.48,1.29)	⊕⊕◯◯ LOW
UC vs. ASA	0.94 (0.53,1.69)	⊕⊕◯◯ LOW [a,f,g]	0.97 (0.53,1.79)	⊕⊕⊕◯ MODERATE [¶]	0.96 (0.63,1.47)	⊕⊕⊕◯ MODERATE
UC+ST vs. ASA			1.43 (0.69,2.97)	⊕⊕◯◯ LOW [++]	1.43 (0.69,2.97)	⊕⊕◯◯ LOW
CONTROL vs. ASA+CLP			0.64 (0.09,4.84)	⊕⊕◯◯ LOW [++]	0.64 (0.09,4.84)	⊕⊕◯◯ LOW
DBG-110MG vs. ASA+CLP			0.88 (0.48,1.63)	⊕⊕◯◯ LOW [++]	0.88 (0.48,1.63)	⊕⊕◯◯ LOW
DBG-150MG vs. ASA+CLP			0.90 (0.49,1.66)	⊕⊕◯◯ LOW [++]	0.90 (0.49,1.66)	⊕⊕◯◯ LOW
EDX-30MG vs. ASA+CLP			0.78 (0.44,1.37)	⊕⊕◯◯ LOW [++]	0.78 (0.44,1.37)	⊕⊕◯◯ LOW
EDX-60MG vs. ASA+CLP			0.62 (0.35,1.09)	⊕⊕◯◯ LOW [++]	0.62 (0.35,1.09)	⊕⊕◯◯ LOW
RVX vs. ASA+CLP			0.53 (0.30,0.95)	⊕⊕◯◯ LOW [++]	0.53 (0.30,0.95)	⊕⊕◯◯ LOW
UC vs. ASA+CLP	0.65 (0.38,1.09)	⊕⊕◯◯ LOW [a,c]	Not estimable	Not estimable *	0.65 (0.38,1.09)	⊕⊕◯◯ LOW
UC+ST vs. ASA+CLP			0.96 (0.44,2.12)	⊕⊕◯◯ LOW [++]	0.96 (0.44,2.12)	⊕⊕◯◯ LOW
DBG-110MG vs. CONTROL			1.37 (0.19,9.90)	⊕⊕◯◯ LOW [++]	1.37 (0.19,9.90)	⊕⊕◯◯ LOW
DBG-150MG vs. CONTROL			1.40 (0.19,10.13)	⊕⊕◯◯ LOW [++]	1.40 (0.19,10.13)	⊕⊕◯◯ LOW
EDX-30MG vs. CONTROL			1.21 (0.17,8.62)	⊕⊕◯◯ LOW [++]	1.21 (0.17,8.62)	⊕⊕◯◯ LOW
EDX-60MG vs. CONTROL			0.96 (0.13,6.83)	⊕⊕◯◯ LOW [++]	0.96 (0.13,6.83)	⊕⊕◯◯ LOW
RVX vs. CONTROL			0.82	⊕⊕◯◯	0.82 (0.12,5.89)	⊕⊕◯◯

			(0.12,5.89)	LOW [++]		LOW
UC vs. CONTROL	1.00 (0.14, 7.14)	⊕⊕◯◯ LOW [a,d]	Not estimable	Not estimable [*]	1.00 (0.14,7.07)	⊕⊕◯◯ LOW
UC+ST vs. CONTROL			1.50 (0.20,11.51)	⊕⊕◯◯ LOW [++]	1.50 (0.20,11.51)	⊕⊕◯◯ LOW
DBG-150MG vs. DBG-110MG	1.02 (0.76,1.37)	⊕⊕⊕◯ MODERATE [a]	1.02 (0.76,1.37)	⊕⊕⊕◯ MODERATE [¶]	1.02 (0.76,1.37)	⊕⊕⊕◯ MODERATE
EDX-30MG vs. DBG-110MG			0.88 (0.60,1.30)	⊕⊕⊕◯ MODERATE [++]	0.88 (0.60,1.30)	⊕⊕⊕◯ MODERATE
EDX-60MG vs. DBG-110MG			0.70 (0.47,1.04)	⊕⊕⊕◯ MODERATE [++]	0.70 (0.47,1.04)	⊕⊕⊕◯ MODERATE
RVX vs. DBG-110MG			0.60 (0.40,0.91)	⊕⊕⊕◯ MODERATE [++]	0.60 (0.40,0.91)	⊕⊕⊕◯ MODERATE
UC vs. DBG-110MG	0.73 (0.53,1.01)	⊕⊕⊕◯ MODERATE [a]	0.73 (0.53,1.01)	⊕⊕⊕◯ MODERATE [¶]	0.73 (0.53,1.01)	⊕⊕⊕◯ MODERATE
UC+ST vs. DBG-110MG			1.09 (0.56,2.14)	⊕⊕◯◯ LOW [++]	1.09 (0.56,2.14)	⊕⊕◯◯ LOW
EDX-30MG vs. DBG-150MG			0.86 (0.59,1.27)	⊕⊕⊕◯ MODERATE [++]	0.86 (0.59,1.27)	⊕⊕⊕◯ MODERATE
EDX-60MG vs. DBG-150MG			0.68 (0.46,1.01)	⊕⊕⊕◯ MODERATE [++]	0.68 (0.46,1.01)	⊕⊕⊕◯ MODERATE
RVX vs. DBG-150MG			0.59 (0.39,0.88)	⊕⊕⊕◯ MODERATE [++]	0.59 (0.39,0.88)	⊕⊕⊕◯ MODERATE
UC vs. DBG-150MG	0.71 (0.52,0.98)	⊕⊕⊕◯ MODERATE [a]	0.72 (0.52,0.99)	⊕⊕⊕◯ MODERATE [¶]	0.72 (0.52,0.99)	⊕⊕⊕◯ MODERATE
UC+ST vs. DBG-150MG			1.07 (0.55,2.09)	⊕⊕◯◯ LOW [++]	1.07 (0.55,2.09)	⊕⊕◯◯ LOW
EDX-60MG vs. EDX-30MG	0.78 (0.63,0.99)	⊕⊕⊕◯ MODERATE [a]	0.79 (0.63,0.99)	⊕⊕⊕◯ MODERATE [¶]	0.79 (0.63,0.99)	⊕⊕⊕◯ MODERATE
RVX vs. EDX-30MG			0.68 (0.48,0.95)	⊕⊕⊕◯ MODERATE [++]	0.68 (0.48,0.95)	⊕⊕⊕◯ MODERATE
UC vs. EDX-30MG	0.83 (0.66,1.03)	⊕⊕⊕◯ MODERATE [a]	0.83 (0.67,1.03)	⊕⊕⊕◯ MODERATE [a]	0.83 (0.67,1.03)	⊕⊕⊕◯ MODERATE
UC+ST vs. EDX-30MG			1.24 (0.66,2.33)	⊕⊕◯◯ LOW [++]	1.24 (0.66,2.33)	⊕⊕◯◯ LOW
RVX vs. EDX-60MG			0.86 (0.61,1.22)	⊕⊕⊕◯ MODERATE [++]	0.86 (0.61,1.22)	⊕⊕⊕◯ MODERATE
UC vs. EDX-60MG	1.04 (0.83,1.32)	⊕⊕⊕◯ MODERATE [a]	1.05 (0.83,1.33)	⊕⊕⊕◯ MODERATE [a]	1.05 (0.83,1.33)	⊕⊕⊕◯ MODERATE
UC+ST vs. EDX-60MG			1.57 (0.83,2.96)	⊕⊕◯◯ LOW [++]	=1.57 (0.83,2.96)	⊕⊕◯◯ LOW
UC vs. RVX	1.22 (0.94,1.56)	⊕⊕⊕◯ MODERATE [a]	Not estimable	Not estimable [*]	1.22 (0.94,1.58)	⊕⊕⊕◯ MODERATE
UC+ST vs. RVX			1.82 (0.95,3.47)	⊕⊕◯◯ LOW [++]	1.82 (0.95,3.47)	⊕⊕◯◯ LOW
UC+ST vs. UC	1.49 (0.83,2.70)	⊕⊕◯◯ LOW [a,h]	Not estimable	Not estimable [*]	1.49 (0.83,2.70)	⊕⊕◯◯ LOW

[a] Unblinded participants and personnel
[b] Confidence interval included potential for important harm or benefits
[c] Imprecise due to low number of events
[d] Wide confidence interval
[e] Large I_2 (≥50%)
[f] Unblinded outcome assessment
[g] Heterogeneity on the age eligibility among trials
[h] Mixed population (inclusive of AF, DVT, PE, etc)
[§]Contributing direct evidence of low/very low quality
[¶]Contributing direct evidence of moderate quality
[++]Indirect estimate from order loops higher than first order
[*]Cannot be estimated because drug was not connected in a loop in the evidence network

References of included studies

1 Pink, J., Pirmohamed, M., Lane, S. & Hughes, D. A. Cost-effectiveness of pharmacogenetics-guided warfarin therapy vs. alternative anticoagulation in atrial fibrillation. *Clinical pharmacology and therapeutics* **95**, 199-207, doi:10.1038/clpt.2013.190 (2014).

2 Sharma, P. *et al.* Is self-monitoring an effective option for people receiving long-term vitamin K antagonist therapy? A systematic review and economic evaluation. *BMJ open* **5**, e007758, doi:10.1136/bmjopen-2015-007758 (2015).

3 Reddy, V. Y. *et al.* Percutaneous left atrial appendage closure vs warfarin for atrial fibrillation: a randomized clinical trial. *Jama* **312**, 1988-1998, doi:10.1001/jama.2014.15192 (2014).

4 Holmes, D. R., Jr. *et al.* Prospective randomized evaluation of the Watchman Left Atrial Appendage Closure device in patients with atrial fibrillation versus long-term warfarin therapy: the PREVAIL trial. *Journal of the American College of Cardiology* **64**, 1-12, doi:10.1016/j.jacc.2014.04.029 (2014).

5 Matchar, D. B. *et al.* Effect of home testing of international normalized ratio on clinical events. *The New England journal of medicine* **363**, 1608-1620, doi:10.1056/NEJMoa1002617 (2010).

6 Khan, T. I., Kamali, F., Kesteven, P., Avery, P. & Wynne, H. The value of education and self-monitoring in the management of warfarin therapy in older patients with unstable control of anticoagulation. *British journal of haematology* **126**, 557-564, doi:10.1111/j.1365-2141.2004.05074.x (2004).

7 Voller, H. *et al.* Self-management of oral anticoagulation in nonvalvular atrial fibrillation (SMAAF study). *Zeitschrift fur Kardiologie* **94**, 182-186, doi:10.1007/s00392-005-0199-0 (2005).

8 Menendez-Jandula, B. *et al.* Comparing self-management of oral anticoagulant therapy with clinic management: a randomized trial. *Annals of internal medicine* **142**, 1-10 (2005).

9 Verret, L. *et al.* Impact of a pharmacist-led warfarin self-management program on quality of life and anticoagulation control: a randomized trial. *Pharmacotherapy* **32**, 871-879, doi:10.1002/j.1875-9114.2012.01116 (2012).

10 Pirmohamed, M. *et al.* A randomized trial of genotype-guided dosing of warfarin. *The New England journal of medicine* **369**, 2294-2303, doi:10.1056/NEJMoa1311386 (2013).

11 Connolly, S. *et al.* Clopidogrel plus aspirin versus oral anticoagulation for atrial fibrillation in the Atrial fibrillation Clopidogrel Trial with Irbesartan for prevention of Vascular Events (ACTIVE W): a randomised controlled trial. *Lancet (London, England)* **367**, 1903-1912, doi:10.1016/s0140-6736(06)68845-4 (2006).

12 Liu, X. *et al.* Warfarin compared with aspirin for older Chinese patients with stable coronary heart diseases and atrial fibrillation complications. *International journal of clinical pharmacology and therapeutics* **52**, 454-459, doi:10.5414/cp201996 (2014).

13 Warfarin versus aspirin for prevention of thromboembolism in atrial fibrillation: Stroke Prevention in Atrial Fibrillation II Study. *Lancet (London, England)* **343**, 687-691 (1994).

14 Secondary prevention in non-rheumatic atrial fibrillation after transient ischaemic attack or minor stroke. EAFT (European Atrial Fibrillation Trial) Study Group. *Lancet (London, England)* **342**, 1255-1262 (1993).

15 Rash, A. *et al.* A randomised controlled trial of warfarin versus aspirin for stroke prevention in octogenarians with atrial fibrillation (WASPO). *Age and ageing* **36**, 151-156, doi:10.1093/ageing/afl129 (2007).

16 Lavitola Pde, L. *et al.* Warfarin or aspirin in embolism prevention in patients with mitral valvulopathy and atrial fibrillation. *Arquivos brasileiros de cardiologia* **95**, 749-755 (2010).

17 Mant, J. *et al.* Warfarin versus aspirin for stroke prevention in an elderly community population with atrial fibrillation (the Birmingham Atrial Fibrillation Treatment of the Aged Study, BAFTA): a randomised controlled trial. *Lancet (London, England)* **370**, 493-503, doi:10.1016/s0140-6736(07)61233-1 (2007).

18 Petersen, P., Boysen, G., Godtfredsen, J., Andersen, E. D. & Andersen, B. Placebo-controlled, randomised trial of warfarin and aspirin for prevention of thromboembolic complications in chronic atrial fibrillation. The Copenhagen AFASAK study. *Lancet (London, England)* **1**, 175-179 (1989).
19 Gullov, A. L. *et al.* Fixed minidose warfarin and aspirin alone and in combination vs adjusted-dose warfarin for stroke prevention in atrial fibrillation: Second Copenhagen Atrial Fibrillation, Aspirin, and Anticoagulation Study. *Archives of internal medicine* **158**, 1513-1521 (1998).
20 Singer, D. E. *et al.* The effect of low-dose warfarin on the risk of stroke in patients with nonrheumatic atrial fibrillation. *The New England journal of medicine* **323**, 1505-1511, doi:10.1056/nejm199011293232201 (1990).
21 Connolly, S. J. *et al.* Canadian Atrial Fibrillation Anticoagulation (CAFA) Study. *Journal of the American College of Cardiology* **18**, 349-355 (1991).
22 Sato, H. *et al.* Low-dose aspirin for prevention of stroke in low-risk patients with atrial fibrillation: Japan Atrial Fibrillation Stroke Trial. *Stroke* **37**, 447-451, doi:10.1161/01.STR.0000198839.61112.ee (2006).
23 Chen, K. P. *et al.* Anticoagulation therapy in Chinese patients with non-valvular atrial fibrillation: a prospective, multi-center, randomized, controlled study. *Chinese medical journal* **125**, 4355-4360 (2012).
24 Stroke Prevention in Atrial Fibrillation Study. Final results. *Circulation* **84**, 527-539 (1991).
25 Connolly, S. J. *et al.* Dabigatran versus warfarin in patients with atrial fibrillation. *The New England journal of medicine* **361**, 1139-1151, doi:10.1056/NEJMoa0905561 (2009).
26 Patel, M. R. *et al.* Rivaroxaban versus warfarin in nonvalvular atrial fibrillation. *The New England journal of medicine* **365**, 883-891, doi:10.1056/NEJMoa1009638 (2011).
27 Ogawa, S., Shinohara, Y. & Kanmuri, K. Safety and efficacy of the oral direct factor xa inhibitor apixaban in Japanese patients with non-valvular atrial fibrillation. -The ARISTOTLE-J study. *Circulation journal : official journal of the Japanese Circulation Society* **75**, 1852-1859 (2011).
28 Giugliano, R. P. *et al.* Edoxaban versus warfarin in patients with atrial fibrillation. *The New England journal of medicine* **369**, 2093-2104, doi:10.1056/NEJMoa1310907 (2013).
29 Granger, C. B. *et al.* Apixaban versus warfarin in patients with atrial fibrillation. *The New England journal of medicine* **365**, 981-992, doi:10.1056/NEJMoa1107039 (2011).
30 Weitz, J. I. *et al.* Randomised, parallel-group, multicentre, multinational phase 2 study comparing edoxaban, an oral factor Xa inhibitor, with warfarin for stroke prevention in patients with atrial fibrillation. *Thrombosis and haemostasis* **104**, 633-641, doi:10.1160/th10-01-0066 (2010).
31 Hori, M. *et al.* Rivaroxaban vs. warfarin in Japanese patients with atrial fibrillation - the J-ROCKET AF study. *Circulation journal : official journal of the Japanese Circulation Society* **76**, 2104-2111 (2012).
32 Hong, K. S. *et al.* Rivaroxaban vs Warfarin Sodium in the Ultra-Early Period After Atrial Fibrillation-Related Mild Ischemic Stroke: A Randomized Clinical Trial. *JAMA neurology* **74**, 1206-1215, doi:10.1001/jamaneurol.2017.2161 (2017).
33 Chung, N. *et al.* Safety of edoxaban, an oral factor Xa inhibitor, in Asian patients with non-valvular atrial fibrillation. *Thrombosis and haemostasis* **105**, 535-544, doi:10.1160/th10-07-0451 (2011).
34 Ezekowitz, M. D. *et al.* Dabigatran with or without concomitant aspirin compared with warfarin alone in patients with nonvalvular atrial fibrillation (PETRO Study). *The American journal of cardiology* **100**, 1419-1426, doi:10.1016/j.amjcard.2007.06.034 (2007).
35 Mao, L., Li, C., Li, T. & Yuan, K. Prevention of stroke and systemic embolism with rivaroxaban compared with warfarin in Chinese patients with atrial fibrillation. *Vascular* **22**, 252-258, doi:10.1177/1708538113490423 (2014).

36 Yamashita, T. *et al.* Randomized, multicenter, warfarin-controlled phase II study of edoxaban in Japanese patients with non-valvular atrial fibrillation. *Circulation journal : official journal of the Japanese Circulation Society* **76**, 1840-1847 (2012).
37 Connolly, S. J. *et al.* Apixaban in patients with atrial fibrillation. *The New England journal of medicine* **364**, 806-817, doi:10.1056/NEJMoa1007432 (2011).
38 Shosha, R. I., Ibrahim, O. M., Setiha, M. E. & Abdelwahab, A. A. The Efficacy and Safety of Rivaroxaban as an Alternative to Warfarin for the Prevention of Thromboembolism in Patients with Atrial Fibrillation. *Int J Pharm Sci Rev Res* **43**, 38-48 (2017).
39 Mehran, R. *et al.* Standardized bleeding definitions for cardiovascular clinical trials: a consensus report from the Bleeding Academic Research Consortium. *Circulation* **123**, 2736-2747, doi:10.1161/circulationaha.110.009449 (2011).
